# Identity of major sulfur-cycle prokaryotes in freshwater lake ecosystems revealed by a comprehensive phylogenetic study of the dissimilatory adenylylsulfate reductase

**DOI:** 10.1038/srep36262

**Published:** 2016-11-08

**Authors:** Tomohiro Watanabe, Hisaya Kojima, Manabu Fukui

**Affiliations:** 1 The Institute of Low Temperature Science, Hokkaido University, Sapporo, Japan

## Abstract

Adenylylsulfate reductase is a heterodimeric complex of two subunits, AprB and AprA, and is a key enzyme in dissimilatory sulfate reduction and sulfur oxidation. Common use of *aprA* as a functional marker gene has revealed the diversity of sulfur-cycle prokaryotes in diverse environments. In this study, we established a comprehensive sequence set of *apr* genes and employed it to reanalyze *apr* phylogeny, evaluate the coverage of a widely used primer set (AprA-1-FW/AprA-5-RV), and categorize environmental *aprA* sequences. Phylogenetic tree construction revealed new members of Apr lineage II and several previously unrecognized lateral gene transfer events. Using the established phylogenetic tree, we classified all previously reported *aprA* sequences amplified from freshwater lakes with the primer pair AprA-1-FW/AprA-5-RV in addition to the *aprA* sequences newly retrieved from freshwater lakes; the obtained results were complemented by 16S rRNA clone library analysis. Apr-based classifications of some of operational taxonomic units were supported by 16S rRNA-based analysis. This study updates our knowledge on the phylogeny of *aprBA* and shows the identities of several sulfur-cycle bacteria, which could not be classified to a known taxa until now. The established *apr* sequence set is publicly available and can be applied to assign environmental sequences to known lineages.

Dissimilatory adenylylsulfate reductase catalyzes the conversion of adenosine-5′-phosphosulfate (APS) to sulfite and adenosine monophosphate (AMP) in sulfate-reducing prokaryotes and is also postulated to catalyze the reverse reactions in sulfur-oxidizing prokaryotes. The functional unit of this protein is suggested as a heterodimeric complex of a two [4Fe-4S] clusters containing beta-subunit and a FAD-containing alpha-subunit^1^,^2^,^3^, and these subunits are encoded by *aprB* and *aprA*, respectively. The *aprBA* gene locus is conserved in the cultured sulfate reducers of six phyla, *Euryarchaeota*, *Crenarchaeota*, *Thermodesulfobacteria*, *Firmicutes*, *Nitrospirae*, *Proteobacteria*, and patchily distributed in sulfur oxidizers belonging to archaeal (*Crenarchaeota*) and bacterial phyla (*Chlorobi* and *Proteobacteria*). The high conservation of the *aprBA* gene locus in both sulfate reducers and sulfur oxidizers led Meyer and Kuever to design the primer set AprA-1-FW/AprA-5-RV to track microorganisms involved in sulfur cycling in diverse environments^4^. Despite the fact that there are sulfur oxidation pathways independent of APS and some sulfur oxidizers lack the *aprBA* genes, the common use of this primer set has revealed a hidden diversity of adenylylsulfate reductase and provided a more complete picture of the dissimilatory microbial sulfur cycle in comparison with when applying universal primers targeting the 16S rRNA gene.

To obtain an accurate description of the phylogenetic affiliation of *aprA* sequences derived from environmental samples, several problems should be considered. The first problem is the phylogenetic complexity caused by lateral gene transfer (LGT) events. Earlier studies revealed that the *aprBA* gene locus has frequently experienced LGT events across distantly related phylogenetic taxa^5^,^6^. The resulting phylogenetic complexity hampers the assignment of environmentally derived *aprA* to specific phylogenetic taxa. For example, two previous *aprA*-cloning studies recovered a large number of sequences related to both the sulfur-oxidizing genus *Chlorobi* and the sulfate-reducing genus *Thermodesulfovibrio*, and these sequences could not be assigned to either the reductive or oxidative lineage of AprA due to their close phylogenetic relationship^7^,^8^. The second problem is the low phylogenetic resolution of environmentally derived *aprA* sequences, which typically have a small number of comparable amino acid positions. Earlier studies indicated that phylogenetic trees based on partial AprA sequences showed very low bootstrap values^4^,^8^,^9^,^10^,^11^,^12^. To overcome these two problems, the following approaches are conceivable: (1) comparison of the phylogeny of the *aprA* gene with that of other marker genes such as 16S rRNA gene, *dsrAB*, sqr and *soxB* and (2) classification of environmentally derived *aprA* sequences based on the robust phylogenetic tree constructed with full sequences of adenylylsulfate reductase. As the third problem it should be considered that some sulfur oxidizers possess two *aprBA* gene loci, which are phylogenetically divided to distinct groups: *apr* lineage I and II^5^. The fourth problem is the limited number of available reference sequences of characterized species. This problem not only exists for *aprA* but also for other functional marker genes. The recent development of high-throughput technologies has provided new *aprBA* sequences from a number of prokaryotes, and these sequences may provide a clue to determine taxonomic affiliation of unclassified environmentally derived sequences.

In this study, we provided new *aprBA* sequences obtained from isolated organisms and established a comprehensive sequence set using publicly available *apr* sequences. Based on nearly full-length manually aligned AprBA sequences, we created a robust phylogenetic tree, which has enlarged species coverage in comparison with earlier studies. The constructed AprBA tree advanced our understanding of its phylogeny and was applied for the classification of environmental *aprA* sequences obtained from freshwater lakes. Previous studies have shown that freshwater lakes sustain diverse microorganisms harboring *aprA*^7^,^8^,^10^,^11^, although the taxonomic identities of most of these microorganisms remain unclear. In this study, clone libraries of PCR-amplified *aprA* fragments from seven freshwater lakes were analyzed along with those of the 16S rRNA gene. The combined analysis of these genes revealed the taxonomic identities of some of the sulfur-cycle bacteria in freshwater lake ecosystems.

## Results and Discussion

### Sequence collection

Sequences of *aprBA* were newly obtained from several strains maintained in our laboratory (Supplementary Table S1). Bacterial and archaeal *apr* sequences with defined taxonomic attribution (at least to the phylum level) were retrieved from NCBI. A partial *aprA* sequence (named “Mizugaki enrichment phylotype-AprA”) from a thiosulfate-disproportionating enrichment culture was also included in this study (see “Methods” for details). The obtained sequence assemblage is represented by a core sequence set of 320 sequences that cover the nearly full length of AprB (>101 amino acid residues) and AprA (>554 amino acid residues) and 154 shorter sequences containing more than 118 amino acid residues of AprA. The constructed sequence set is available in Excel files (Supplementary Tables S2 and S3).

### Comparative phylogenetic analysis of AprB, AprA and AprBA

To provide a comprehensive phylogenetic framework for adenylylsulfate reductase, 320 AprB and AprA sequences in the core sequence set were aligned separately, and both phylogenies were calculated using maximum-likelihood (ML) and neighbor-joining (NJ) methods (Supplementary Figs S1 and S2). Overall, the phylogenetic trees for both subunits showed a consistent separation into 12 major clusters: *Desulfovibrionales*, *Desulfobulbaceae*, *Desulfobacteraceae*, *Thermodesulfobacteriaceae*, *Thermodesulfovibrio*, “*Candidatus* Magnetobacterium” & “*Candidatus* Magnetoglobus”, *Chlorobi*, Gram-positive bacteria & LGT-affected deltaproteobacteria, *Archaeoglobus*, *Thermoproteaceae*, and sulfur-oxidizing proteobacterial lineages I and II. The formation of these clusters was consistent irrespective of the tree inference methods. The positions of the three clusters and four branches with a star in Supplementary Figs S1 and S2 varied between the AprB and AprA trees. One of the most likely explanations for the observed topological discrepancies is the low phylogenetic resolution of AprB sequences, which had a small number of comparable amino acid positions (82 amino acids). Nevertheless, phylogenetic congruence between AprB and AprA was apparent in the branching order of the other clusters, indicating the coevolution of two subunits of both sulfate-reducing and sulfur-oxidizing prokaryotes.

**Figure 1:**
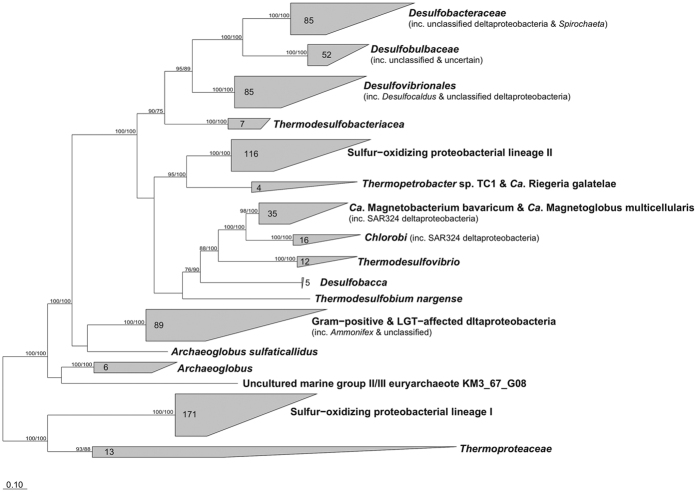
Consensus phylogenetic tree of AprBA sequences. The displayed tree is a maximum likelihood tree constructed based on 320 AprBA sequences of the core database. Remaining 154 sequences of sorter AprA or AprBA and 225 AprA sequences of OTUs obtained in this study were added to the consensus tree by the parsimony interactive tool in ARB, and were included in the clusters. Bootstrap values ≥50% for ML (first) and MP (second) based on 100 resamplings are indicated near the branches. Uncultured marine group II/III euryarchaeote KM3_67_G08 was obtained from a metagenomic library of deep-Mediterranean water^40^.

To improve the phylogenetic resolution, the AprB and AprA alignments were concatenated and a consensus AprBA tree was constructed (see “Methods” for details). For a comprehensive understanding of the AprBA phylogeny, 154 shorter sequences were inserted into the AprBA consensus tree without changing the overall tree topology (Fig. 1). The uncollapsed version of the AprBA consensus tree is provided as supporting data (Supplementary Fig. S3). As shown in Fig. 1, the bootstrap values at the nodes separating the basal clusters of the AprBA consensus tree were improved from those of the phylogenetic tree based on only AprA sequences (Supplementary Figs S1 and S2).

### Phylogeny of adenylylsulfate reductase

The AprBA sequences of sulfur oxidizers were phylogenetically divided into two groups: Apr lineages I and II. Previously, lineage II consisted of the sulfur-oxidizing bacteria belonging to the *Chlorobi* and *Gamma-* and *Beta-proteobacteria*^5^. Herein, we propose the inclusion of several members of *Alphaproteobacteria*, *Nitrospiraceae* and *Deltaproteobacteria* in the Apr lineage II. In the AprBA consensus tree, two alphaproteobacteria—*Thermopetrobacter* sp. TC1 and “*Candidatus* Riegeria galatelae”—formed a cluster out of the cluster of sulfur-oxidizing proteobacterial lineage II (Fig. 1). Although their sulfur-oxidizing capabilities have not been reported, the draft genome of *Thermopetrobacter* sp. TC1 harbors other suites of genes for sulfur oxidation, i.e., reverse-acting dissimilatory sulfite reductase (EF16DRAFT_1183−1196: *dsrABEFHCMKLJOP*x*N*) and periplasmic thiosulfate-oxidizing Sox enzyme complex (EF16DRAFT_2290−2286: *soxXYZAB*). A unique insertion conserved in proteobacterial lineage II-AprB sequences was also found in its AprB sequence^5^ (Supplementary Fig. S4). Sulfur storage in “*Ca*. Riegeria galatelae” was previously reported^13^. Therefore, it is reasonable to group their AprBA sequences into sulfur-oxidizing proteobacterial lineage II. With respect to *Nitrospiraceae*, the AprBA sequences of “*Candidatus* Magnetobacterium bavaricum” and “*Candidatus* Magnetoglobus multicellularis” formed a distinct cluster with the members of the phylum *Chlorobi* (Fig. 1). Their AprBA sequences shared a unique deletion in each subunit (Supplementary Fig. S5). A previous study proposed that the *aprBA* gene locus of the sulfate-reducing genus *Thermodesulfovibrio* was laterally transferred to the *Chlorobi*^5^, although these deletions were absent in the AprBA sequences of all *Thermodesulfovibrio* species (Supplementary Fig. S5). With respect to dissimilatory sulfur metabolism of “*Ca*. M. bavaricum,” electron shuttling between sulfate and sulfide depending on redox conditions was postulated^14^. Although the actual function of adenylylsulfate reductases of “*Ca*. M. bavaricum” and “*Ca*. M. multi cellularis” is not yet known, their AprBA sequences can be phylogenetically regarded as sulfur-oxidizing bacterial lineage II based on the above-mentioned factors. Shorter AprA sequences retrieved from multiple single-cell samples of deltaproteobacterial cluster SAR324 were also considered to belong to Apr lineage II, because these sequences were placed within the clusters of *Chlorobi* and *Nitrospiraceae*^15^ (Figs 1 and S3). The involvement of the SAR324 group in sulfur oxidation has recently been implied by metagenomic, metatranscriptomic^16^ and single-cell genomic analyses^15^.

The consensus AprBA tree in Fig. 1 confirmed the previously proposed major LGT events (e.g., the transfer of *aprBA* from donor strains of Gram-positive sulfate reducers to some of deltaproteobacteria and *Archaeoglobus* species^6^). On the other hand, the enlarged species coverage of this study revealed discrepancies between 16S rRNA- and AprBA-based phylogenies that have not been recognized previously. For instance, *Archaeoglobus sulfaticallidus* branched separately from the other *Archaeoglobus* species in the tree of AprBA (Fig. 1). This branching appears to be the result of at least two independent LGT events within the genus *Archaeoglobus*. One of the other newly discovered deviations from the 16S rRNA gene-based phylogeny was the classification of *Thioalkalivibrio* sp. HK1 into the sulfur-oxidizing proteobacterial lineage II (Supplementary Fig. S3). All of the other *Thioalkalivibrio* species have a lineage I-type AprBA. This finding suggests a putative xenologous replacement of lineage I with lineage II, supporting the proposed evolutionary scenario of sulfate reducer-related adenylylsulfate reductase in sulfur-oxidizing bacteria^5^.

A more notable finding was LGT event involving a bacterial phylum which has not been taken into consideration. The *aprBA* gene locus was identified in the draft genome of *Spirochaeta odontotermitis* JC202, as the first case in the phylum *Spirochaetes*. Although spirochetes have been found in sulfidic environments such as the enrichment cultures of sulfate reducers and sulfur ‘Thiodendron’ mat^17^, their genes involved in dissimilatory sulfur metabolism have not been identified. The genome of *Spirochaeta odontotermitis* JC202 also encodes other key enzymes involved in dissimilatory sulfate reduction (Supplementary Fig. S6), i.e., sulfate adenylyltransferase (Sat) and heterodisulfide reductase (Hdr) complex which is considered to serve as the electron donor of AprBA^18^,^19^. The amino acid sequences of Apr, Sat and Hdr were consistently showed close phylogenetic relationship with the members of the family *Desulfobacteraceae* (Fig. 1, Supplementary Figs S1, S2 and S6), suggesting a recent homologous recombination of the spirochete genome with the genomic DNA of a member of the *Desulfobacteraceae*. Especially, the AprA sequence of *Spirochaeta odontotermitis* JC202 was identical to the partial AprA sequences of Delta 1 symbionts of the *Olavius* species. Previously, the co-occurrence of Delta 1 and spirochete symbionts was observed in geographically distant gutless oligochaetes populations^20^. Stable coexistence as symbionts of same host may enhance the chance of interspecies gene transfer. Another characteristic of *Spirochaeta odontotermitis* JC202 may also be related to the incorporation of the external *apr* genes. For this strain, capability of the natural competence was suggested by its genome analysis^21^.

The very close phylogenetic relationship of *aprBA* between *Spirochaeta* and *Desulfobacteraceae* emphasizes the occurrence of a recent lateral transfer. As another case of recent LGT event, transfer of the *aprBA* from *Thermodesulfovibrio* species to *Thermacetogenium phaeum* was postulated in a previous study^6^, on the basis of the Apr-based close phylogenetic relationship. However, the gene locus which had been regarded as *aprBA* of *Thermacetogenium* (accession number EF442974) has been identified as that of *Thermodesulfovibrio* sp., a sulfate-reducing contaminant found in the deposited culture. *Thermacetogenium phaeum* was originally described as a sulfate reducer^22^, but a subsequent study demonstrated that this bacterium lacks the ability of sulfate reduction and *aprBA* genes were not found in its genome^23^.

### *In silico* evaluation of species coverage of AprA-1-FW and AprA-5-RV

For the investigation of functional gene diversity in the environment, PCR biases must be carefully evaluated for the primer combination used. We evaluated the *in silico* coverage of the primer set used in this study (AprA-1-FW/AprA-5-RV) by applying the core dataset to complement the previous work, which assessed the species coverage of AprA-1-FW/AprA-5-RV based on the PCR assays using DNA templates^4^. The species coverage of this primer set is shown in Supplementary Fig. S7. A previous study reported the inability to yield amplicons from the *Desulfosporosinus* spp., and we confirmed the presence of 4 and 0–3 mismatches in the respective forward and reverse primer-binding sites of *Desulfosporosinus* spp. Additionally, our analysis revealed the presence of more than 5 and 4 mismatches of the respective forward and reverse primers with *aprA* sequences of the crenarchaeotal genera *Thermodesulfobium*, *Thermoproteus* and *Pyrobaculum*. The above-mentioned mismatches of the *aprA* sequences of the *Desulfosporosinus* and *Crenarchaeota* appear to result in their avoiding detection in environmental samples. In fact, their close relatives were not detected in the clone libraries analyzed in the present study as described below.

### Effectiveness of the AprA consensus tree for the classification of environmental *aprA*

The phylogenetic framework of adenylylsulfate reductase was applied to reveal the diversity of sulfur-cycle prokaryotes in the freshwater lake ecosystem. We analyzed all previously published clone libraries of *aprA* fragments amplified from freshwater lakes with the primer pair AprA-1-FW/AprA-5-RV, and we also analyzed newly constructed *aprA* libraries (Table 1). A total of 1068 AprA sequences were grouped into 263 distinct operational taxonomic units (OTUs) with a cutoff value of 0.02, and representative clones of the OTUs were placed into the AprBA consensus tree without changing its topology. For comparison, an ML tree was also constructed based on the partial AprA sequences (tree not shown). The taxonomic affiliations of all OTUs were inferred on the basis of two phylogenetic trees and were then compared. For more than half of the OTUs, their affiliations varied depending on trees used for taxonomic inferences (Supplementary Table S4). For example, three OTUs (FTR70-OTU3, FTR90-OTU21, Miz-OTU17) were classified into *Desulfovibrionales* by the AprBA consensus tree based-analysis, although their positions in the partial AprA tree were near the *Thermodesulfovibrio* cluster (FTR70-OTU3) and within the cluster of sulfur-oxidizing proteobacterial lineage II (FTR90-OTU21 and Miz-OTU17). Blastp analysis confirmed that these OTUs are most closely related to *Desulfovibrionales*, supporting the classification based on the AprBA consensus tree. These results indicated that adding partial AprA sequences to the consensus tree is a superior approach to tree construction of a partial AprA tree for the classification of environmental *aprA*.

**Figure 2:**
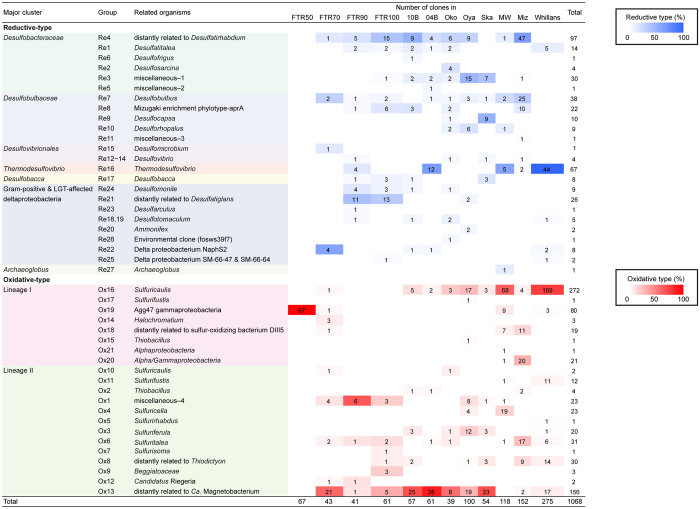
Distribution of clone number per each phylogenetic group defined according to the AprBA consensus tree. Backgrounds of clone numbers are colored according to relative abundances of sulfate reducer-related clones (blue) and sulfur oxidizer-related clones (red) per respective total clone numbers in each library. Group numbers are corresponding to dashed-line boxes in Figure S3. The groups Ox3 and Ox6 contain the clones named as “uncultured *Thiobacillus* spp.” (according to the GenBank record), but evidence to assign these clones to *Thiobacillus* have not been reported. Therefore, Ox3 and Ox6 are most likely to be *Sulfuriferula* and *Sulfuritalea*, respectively. Miscellaneous: 1, partial AprA of *Desulfofaba gelida* DSM 12344, *Desulfonema ishimotonii* DSM 9680 and symbiont of *Olavius algarvensis*; 2, full-length AprBA of *Desulfococcus oleovorans* Hxd3 and partial AprA of *Desulfofaba fastidiosa*; 3, full-length AprBA of *Desulfotalea psychrophila* LSv54 and partial AprBA of *Desulfobulbus* sp. LB2 and *Desulforhopalus vacuolatus* DSM 9700; 4, full-length AprBA (lineage II) of *Thiobacillus thioparus* DSM 505, *T. denitrificans* DSM 12475, *T. denitrificans* DSM 739 and partial AprA (lineage II) of uncultured *Thiobacillus* sp. clone and endosymbionts of *Oligobrachia mashikoi*.

The phylogenetic affiliation of each OTU was defined according to the AprBA consensus tree. OTUs in the same cluster were grouped together as indicated in Supplementary Fig. S3 (groups Re1−27 and Ox1−21), and the distribution of the clone number per each phylogenetic group is shown in Fig. 2. To complement the results of the Apr-based phylogenetic affiliation, we also analyzed 16S rRNA gene clone libraries constructed from DNA samples used for the construction of *aprA* libraries (Supplementary Table S5 and Fig. S8). Although the phylogeny of *aprA* and 16S rRNA genes could not be directly linked, the congruent phylogenies of these genes were shown in seven phylogenetic groups, suggesting their presence in the same bacteria. We discuss these bacteria below.

### Identification of sulfate-reducing bacteria in freshwater lakes

Reductive-type AprA sequences were affiliated with 27 distinct phylogenetic groups. The most frequently detected group was Re4, which was detected from 6 lakes (Fig. 2). The microorganisms detected as this AprA phylotype are considered to be one of the common sulfate-reducing bacteria in freshwater lakes. The clones of Re4 were distantly related to *Desulfatirhabdium*, and most frequently detected in the oxygen-depleted water of Lake Mizugaki (Fig. S3). From the same water samples, the 16S rRNA gene sequences distantly related to *Desulfatirhabdium butyrativorans* were also frequently detected (Supplementary Fig. S8a). It is possible that these 16S rRNA gene sequences correspond to Re4, although there is no solid evidence at present.

The AprA clones in Re8 were related to “Mizugaki enrichment phylotype-AprA,” which is derived from a thiosulfate-disproportionating culture exclusively dominated by “Mizugaki enrichment phylotype-16S” (please see “Methods” for details). The 16S rRNA gene-based analysis confirmed that three 16S rRNA gene clones from Lake Mizugaki shared 98% sequence similarity with “Mizugaki enrichment phylotype-16S” (Supplementary Fig. S8a). The microorganisms corresponding to the Re8 may disproportionate thiosulfate in freshwater lakes.

Only one 16S rRNA gene clone in r-Miz-OTU51 was affiliated with the genus *Desulfobulbus* (Supplementary Fig. S8a), and AprA sequences related to this genus were frequently detected in Lake Mizugaki (Fig. 2). In the genus *Desulfobulbus*, the NaCl requirement varied among species^24^ and r-Miz-OTU51 was clustered with the freshwater species, *D. propionicus* and *D. elongatus*. As discussed previously, the ability of the genus *Desulfobulbus* to use various electron acceptors other than sulfate is considered to be advantageous for survival in low-sulfate environments such as freshwater lakes^8^. Additionally, reduced sulfur compounds might be disproportinated by the putative members of *Desulfobulbus*, because *Desulfobulbus propionicus* is able to disproportionate elemental sulfur and *Desulfobulbus*-related genus, *Desulfocapsa*, consists of only sulfur compounds-disproportionating bacteria^25^,^26^.

### Identification of sulfur-oxidizing bacteria in freshwater lakes

Oxidative-type AprA sequences were grouped into 21 different phylogenetic groups. As the most frequently detected phylotype, a total of 272 clones were affiliated with a single group (Ox16). These clones were found in all freshwater lakes investigated in this study and accounted for more than a half of the AprA clones retrieved from the sediments of Lake Maruwan O-Ike and Lake Whillans. Purcell and co-workers previously classified the AprA sequences of Ox16 (corresponding to “Whillans-OTU21−34 in Supplementary Table S4) into the genus “*Sideroxydans*”^7^. These sequences were related to this betaproteobacterial genus, although the AprA sequences of Ox16 formed a monophyletic cluster with the AprA sequence of *Sulfuricaulis limicola* (Supplementary Fig. S3), which is a recently isolated freshwater sulfur-oxidizing gammaproteobacterium^27^. The affiliation of the AprA sequences of Ox16 with the genus *Sulfuricaulis* was also supported by the 16S rRNA gene-based analysis, i.e., seven 16S rRNA gene clones retrieved from Lake Maruwan O-Ike formed a monophyletic cluster with *Sulfuricaulis limicola* (16S rRNA gene similarities were 98%) (Supplementary Fig. S8b). The genus *Sulfuricaulis* might be a ubiquitous group of a sulfur-oxidizing bacterial community in freshwater lakes.

As the second most frequently detected AprA phylotype, a total of 156 clones were affiliated with a single group (Ox13). This group was found in all freshwater lakes except for Maruwan O-Ike and was detected as the most dominant *aprA* phylotype in Lake Biwa and Skallen O-Ike. In the sediments of Lake Biwa, the group Ox13 was detected in two sediment samples collected in different years (2004 and 2010), suggesting that this group has existed in this lake for many years. Ox13 included a number of environmental *aprA* sequences reported from freshwater lakes. Previously, whether microorganisms represented by these sequences are involved in sulfate reduction or sulfur oxidation could not be evaluated due to the close phylogenetic distances between the AprA sequences of the sulfur-oxidizing and sulfate-reducing genera *Chlorobi* and *Thermodesulfovibrio*, respectively^7^,^8^,^9^. However, in the AprBA consensus tree, the group Ox13 formed a bootstrap-supported cluster with deltaproteobacterial SAR324 clade bacteria and “*Ca.* Magnetobacterium” and their cluster was completely separated from the *Thermodesulfovibrio* cluster with bootstrap confidence (Supplementary Figs 1 and S3). In a water column of the Feitsui Reservoir, where the ratio of the putative sulfate-reducing and sulfur-oxidizing population was changed depending on the water depth, approximately 80% of the *aprA* sequences of group Ox13 were detected in oxygen-containing water at a depth of 70 m, and the others were detected in oxygen-depleted water at 90 and 100 m as a minor component (Fig. 2). Such vertical distribution supports the idea that Ox13 corresponds to a sulfur oxidizer. The 16S rRNA gene-based analysis indicated that eight clones from Lake Biwa and Lake Skallen O-Ike formed a cluster distantly related to “*Ca.* Magnetobacterium” (Supplementary Fig. S8c), which possesses lineage II Apr, but none of the retrieved 16S rRNA gene sequences were related to the SAR324 clade deltaproteobacteria (Supplementary Fig. S8a). Therefore, we concluded that the microorganisms represented by AprA sequences of Ox13 are sulfur-oxidizing bacteria of the *Nitrospiraceae*, although oxidation of reduced sulfur compounds as an energy source has never been reported from this family.

Among the 16S rRNA gene clones affiliated with *Betaproteobacteria*, 18 clones from Lake Maruwan O-Ike were closely related to the members of a freshwater sulfur-oxidizing genus *Sulfuricella* (Supplementary Fig. S8d), which was one of the dominant AprA phylotypes in this lake (Fig. 2), as indicated by a previous study^8^. These 16S rRNA gene clones shared 99% sequence identity with *Sulfuricella denitrificans* skB26, supporting the dominance of *Sulfuricella* in this lake. Therefore, this study revealed that the genera *Sulfuricaulis* and *Sulfuricella* dominate the sulfur-oxidizing bacterial community in the sediment of Lake Maruwan O-Ike. This is the first report of the dominance of both beta- and gamma-proteobacterial sulfur oxidizers in freshwater lake sediment.

Previously, sulfur-oxidizing betaproteobacteria of the genus *Sulfuritalea* were reported as one of the major constituents in Lake Mizugaki^11^. Our comprehensive AprA-based analysis showed evidence for the wide distribution of these sulfur oxidizers across geographically distant freshwater lakes (Fig. 2).

## Conclusion

In this study, we constructed a comprehensive *aprBA* sequence set that contains newly determined and previously published sequences. The detailed phylogenetic analysis of AprBA sequences revealed new members affiliated with Apr lineage II and previously unrecognized LGT events. We further applied the established phylogenetic framework to the PCR-based analyses of environmental *aprA*, and provided a more complete picture of the dissimilatory microbial sulfur cycle in the freshwater lake ecosystem. A portion of the main constituents was identified with the support of a 16S rRNA-based analysis. The sequence set established in this study is provided in Excel files (Supplementary Tables S2 and S3) and can be applied to assign environmental *apr* sequences to known taxa.

## Methods

### Construction of a sequence set of adenylylsulfate reductase

The genomes of *Sulfurirhabdus autotrophica* BiS0^T^, *Sulfuriferula multivorans* TTN^T^, and *Sulfurisoma sediminicola* BSN1^T^ were sequenced using next-generation sequencing technologies. The *aprBA* genes of these genomes were identified using blastn search and have been deposited in public databases. The nucleotide and amino acid sequences of *aprA* and *aprB* genes were also retrieved from the NCBI nucleotide and genome databases using keywords “aprA,” “adenylyl (−) sulfate reductase,” “adenylyl (−) sulphate reductase” and “adenosine-5′-phosphosulfate reductase”. From the obtained *aprA* sequences, 89 representative sequences encompassing the known *aprA* diversity were manually selected (Supplementary Table S6), and similarity searches were performed by blastn search against the NCBI non-redundant database excluding uncultured/environmental sample sequences. The sequence collection using the public databases was finished on 28 Nov 2014. Only unique *aprBA* sequences were selected, and the sequences derived from microorganisms with defined taxonomic attribution (at least phylum level) were used for further analysis.

Bacterial 16S rRNA gene sequences and *aprA* gene sequences were also obtained from an enrichment culture maintained in our laboratory. Briefly, this enrichment culture was established from anoxic water of Lake Mizugaki, a meromictic freshwater lake, using a defined medium without NaCl under thiosulfate disproportionation conditions (see “Supplementary methods” for details). Bacterial 16S rRNA gene sequences and *aprA* gene sequences were amplified from an enrichment culture using the primer pairs 27F/1492R and AprA-1-FW/AprA-5-RV, respectively^4^,^28^. The resulting amplicons were used for the clone library construction, as described below. In total 20 and 12 clones of 16S rRNA and *aprA*, respectively, were sequenced. All 16S rRNA gene clones shared >99.5% nucleotide sequence identity (1484 comparable sites), and a representative clone was named “Mizugaki enrichment phylotype-16S” (LC124812). In pairwise comparisons of the all *aprA* gene clones, only 2 or fewer amino acid differences were found in 119 positions of deduced amino acid sequences. A representative of these *aprA* clones was named “Mizugaki enrichment phylotype-AprA” (LC124794). It is likely that both phylotypes were derived from a single species, which is a freshwater sulfur-disproportionating species. Therefore, the representative AprA sequence of the enrichment culture was included in the database.

### Construction of an AprBA tree

AprBA phylogeny was determined using a core sequence set including 320 AprBA sequences. The AprB and AprA sequences were aligned separately using the web-server-based ClustalW alignment tool^29^ (http://www.genome.jp/tools/clustalw/), and the resulting alignments were refined manually by visual inspection. Concatenating the AprB and AprA alignments allowed for the construction of an AprBA alignment. The AprB, AprA and AprBA alignments were analyzed separately with the phylogeny inference methods implemented in the ARB and MEGA software packages^30^,^31^. All positions containing gaps and missing data were eliminated. The final numbers of comparable amino acid positions of AprB and AprA were 82 and 503, respectively. MEGA software was used to construct the phylogenetic trees of AprB and AprA according to the NJ and ML methods based on the JTT matrix model^32^. For the construction of the AprBA consensus tree, the ML (RAxML) and maximum parsimony (MP; PHYLIP PROTPARS) trees were inferred using ARB software with 100 bootstrap analysis. The bootstrap values of the MP analysis were overlaid onto the ML tree, and the resulting tree was defined as a consensus tree. The 154 shorter AprBA sequences were inserted into the consensus AprBA tree using the ARB parsimony-interactive method.

### Cloning

DNA samples obtained from freshwater lakes were used for the construction of 16S rRNA gene and *aprA* clone libraries (Table 1). DNA samples from Lake Biwa (at site A on Sep 2004 and Oct 2010), the Feitsui Reservoir (Dec 2013), Okotanpe, Maruwan O-Ike, Oyako Ike, and Sukallen O-Ike were extracted in previous studies^8^,^33^,^34^,^35^; see each reference for descriptions of the sample collection and DNA extraction procedures. The primer set AprA-1-FW/AprA-5-RV was used to amplify the *aprA* gene fragments. PCR amplification was initiated with 3 min of denaturation at 94 °C, followed by 34 cycles of 45 s at 94 °C, 45 s at 55 °C and 45 s at 72 °C with a final elongation at 72 °C for 7 min. The 16S rRNA gene fragments were amplified with the primer set 27F/1492R. The amplification was initiated with 2 min of denaturation at 94 °C. Each thermal cycle consisted of 30 s of denaturation at 94 °C, 30 s of annealing at 45 °C (for Lake Biwa sample collected in 2004) or 55 °C (the others), and 90 s of elongation at 72 °C. The total cycle number was 34 for the Lake Okotanpe sample; 25 for the Lake Maruwan O-Ike, Lake Biwa (2004) and Lake Oyako samples; and 32 for the Lake Skallen O-Ike sample. An additional extension was performed at 72 °C for 10 min. The amplification of the 16S rRNA gene was also performed with DNA extracted from water samples collected at 50, 70 and 100 m of the Feitsui Reservoir under various thermal cycle conditions, although no PCR products were obtained. The amplified fragments were ligated into the pCR2.1-TOPO vector (Invitrogen, Carlsbad, CA), and the obtained vectors were transformed into TOP10 cells (Invitrogen). The cloned inserts were amplified using the vector primers M13 forward and reverse (30 cycles of 94 °C for 1 min, 55 °C for 1 min, 72 °C for 2 min and an additional extension of 72 °C for 7 min), and the resulting PCR products with the expected size of the insert were sequenced.

### Phylogenetic analysis of environmentally derived *aprA*

We analyzed 5 newly constructed *aprA* gene clone libraries and previously published clone libraries (Supplementary Tables 1 and S5). The obtained *aprA* sequences were translated into amino acid sequences using EMBOSS Transeq (http://www.ebi.ac.uk/Tools/st/emboss_transeq/). The pairwise distance matrices were calculated based on a Poisson model using MEGA. The distance matrices were imported into Mothur^36^, and the AprA sequences were grouped into OTUs with a cutoff value of 0.02. The representative sequences were randomly selected from each OTU and were manually aligned according to the alignment based on the core dataset. The alignment was phylogenetically placed into the AprBA consensus tree according to the ARB parsimony interactive method. For comparison, a partial AprA-based tree was constructed using the same dataset. Because 48 partial AprA sequences including Whillans-OTU1−7 did not fully cover the region amplified by AprA-1-FW/AprA-5-RV, these sequences were excluded for further analysis. A total of 689 AprA sequences were aligned using ClustalW. The resulting alignment was imported into ARB, and an ML tree was constructed using the maximum-likelihood method RAxML with 109 comparable amino acid positions. The taxonomic affiliations of all OTUs were inferred from the positions of their representative clones on the AprBA consensus tree and partial AprA-based tree.

### Phylogenetic analysis of environmentally derived 16S rRNA gene sequences

We analyzed 5 newly constructed 16S rRNA gene clone libraries and 3 previously published clone libraries (Supplementary Tables 1 and S5). The sequences from the newly constructed libraries were analyzed using Mallard software version 1.02^37^, and putative chimeras and other anomalous sequences were excluded from further analysis. A total of 681 clones were grouped into 365 OTUs using Mothur software at a cutoff value 0.02 or 0.01 (Supplementary Table S5). The clone sequences representing the respective OTUs were randomly selected and were screened for candidate sulfur-cycle bacteria among all bacterial phyla. The representative sequences were automatically aligned with the SINA web aligner^38^, and the resulting alignment was imported into the ARB and placed on the guide tree of the SILVA SSU Reference database release 123^39^. As a result, 9 and 297 clones were affiliated with the phyla *Nitrospirae* and *Proteobacteria* (including 70 delta-, 7 epsilon-, 64 gamma- and 156 beta-proteobacterial clones), respectively. None of the retrieved sequences were closely related to the known sulfur-cycle bacteria of the phyla *Chlorobi*, *Thermodesulfobacteria* and *Firmicutes*. With respect to the classes *Nitrospira* and *Delta-, Gamma-* and *Beta-proteobacteria*, the 16S rRNA gene sequences of representative clones, their close relatives and appropriate out-group bacteria were aligned using ClustalW, and the resulting alignments were manually curated. The phylogenetic reconstructions for each class were calculated with ARB software. The final numbers of comparable sites of *Nitrospira* and *Delta-, Gamma-* and *Beta-proteobacteria* were 495, 1007, 1263 and 1257, respectively. Trees were constructed using the maximum-likelihood method RAxML. Although 7 clones were affiliated with *Epsilonproteobacteria*, these sequences were not included in the further analysis because epsilonproteobacteria lack an *aprBA* gene locus^40^.

### Nucleotide sequence accession numbers

The sequence data determined in this study have been submitted to the DDBJ/EMBL/GenBank databases under accession numbers: LC124209–LC124211 and LC124215–LC124217, *aprBA* sequences of the isolates; LC124223−LC124495, environmental 16S rRNA sequences; LC124496–LC124793, environmental *aprA* sequences; LC124794–LC124825, *aprA* and 16S rRNA sequences from the thiosulfate-disproportionating enrichment culture.

## Additional Information

**How to cite this article**: Watanabe, T. *et al*. Identity of major sulfur-cycle prokaryotes in freshwater lake ecosystems revealed by a comprehensive phylogenetic study of the dissimilatory adenylylsulfate reductase. *Sci. Rep.* **6**, 36262; doi: 10.1038/srep36262 (2016).

**Publisher’s note:** Springer Nature remains neutral with regard to jurisdictional claims in published maps and institutional affiliations.

## Supplementary Material

Supplementary Information

Supplementary Table S1

Supplementary Table S2

## Figures and Tables

**Table 1:** Descriptions of sampling sites and clone libraries analyzed in this study.

Sampling site (location)	Sample	Date of sampling	Library name	Reference for sample
Lake Biwa (Japan)	sediment (0–2 cm)	Sep. 2004	**a-04B**	34
	sediment (0–2 cm)	Oct. 2010	a-10B	8
Lake Okotanpe (Japan)	sediment (0–1 cm)	Jun. 2007	a-Oko	8
Lake Mizugaki (Japan)	water (25, 35, 43 m)	Oct. 2006	a-Miz	11
Feitsui Reservoir (Taiwan)	water (50 m)	Dec. 2013	**a-FTR50**	33
	water (70 m)	Dec. 2013	**a-FTR70**	33
	water (90 m)	Dec. 2013	**a-FTR90**	33
	water (100 m)	Dec. 2013	**a-FTR100**	33
Lake Maruwan O-Ike (Antarctica)	sediment (0–4 cm)	Dec. 2005	a-MW	8
Lake Oyako Ike (Antarctica)	sediment (0–5 cm)	Jan. 2006	a-Oya	8
Lake Skallen O-Ike (Antarctica)	sediment (0–10 cm)	Dec. 2005	a-Ska	8
Lake Whillans (Antarctica)	sediment	Jan. 2013	a-Whillans	7

Clone library constructed by this study was indicated in bold type. a-Miz was constructed by combining three *aprA* clone libraries generated from water samples from depths of 25, 35 and 43 m^11^. a-MW was constructed by combining two *aprA* clone libraries generated from 0–2 and 2–4 cm sediment cores^8^. a-Whillans was constructed by combing six *aprA* clone libraries described in a previous study^7^.
