# Supplementary material for: Identity of major sulfur-cycle prokaryotes in freshwater lake ecosystems revealed by a comprehensive phylogenetic study of the dissimilatory adenylylsulfate reductase
Source: Sci Rep. 2016 Nov 8;6:36262. doi: 10.1038/srep36262 (PMC5099947; doi:10.1038/srep36262)
Supplement: Supplementary Information [file srep36262-s1.pdf]

Supporting Information

Identity of major sulfur-cycle prokaryotes in freshwater lake ecosystem revealed by a comprehensive phylogenetic study of the dissimilatory adenylylsulfate reductase

Tomohiro Watanabe*, Hisaya Kojima*, Manabu Fukui

The Institute of Low Temperature Science, Hokkaido University, Sapporo, Japan

Running title: Major sulfur-cycle prokaryotes in freshwater lake ecosystem

---

*Corresponding author.

Postal address: The Institute of Low Temperature Science, Kita-19, Nishi-8, Kita-ku, Sapporo 060-0819, Japan

Tel: +81-11-706-6884

Fax: +81-11-706-6884

E-mail: watanabe1986@ pop.lowtem.hokudai.ac.jp

kojimah@pop.lowtem.hokudai.ac.jp

Supplementary methods

Enrichment of thiosulfate-disproportionating bacteria

Enrichment culture was established from anoxic water sample (42 m) of Lake Mizugaki collected in June 2014. The water sample was inoculated into bicarbonate-buffered low-salt defined medium supplemented with 5 mM sodium thiosulfate. The composition of the basal medium was as follows (l^{-1}): 0.2 g $MgCl_2 \cdot 6H_2O$, 0.1 g $CaCl_2 \cdot 2H_2O$, 0.1 g NH_4Cl, 0.1 g KH_2PO_4, 0.1 g KCl, 1 ml trace element solution, 1 ml selenite-tungstate solution, 1 ml vitamin mixture solution, 1 ml vitamin B_{12} solution, 1 ml thiamine solution, 30 ml $NaHCO_3$ solution. All stock solutions were prepared as described previously[1]. A portion of the resulting culture was transferred to the same medium. Subsequent subcultures were carried out in the basal medium containing 10−20 mM sodium thiosulfate and 10−20 mM slurry of poorly crystalline Fe(III) oxide as a scavenger of sulfide generated by thiosulfate disproportionation. The slurry was prepared as described previously[2]. To purify the enriched thiosulfate-disproportionating bacteria, serial dilution was performed two times. From the resulting culture, genomic DNA was extracted using a cetyltrimethylammonium bromide method, and was used for the PCR amplification with gene-specific primer pairs.

Captions for additional separate files: Table S2 and S3.

Table S2. Sequence set of core AprBA sequences, established in this study.

Table S3. Sequence set of shorter AprBA sequences derived from prokaryotes with defined taxonomic attribution at least phylum level, established in this study.

Table S1. Accession numbers of *aprBA* sequences newly determined in this study.

Organism	Accession number	
	aprB	*aprA*
Sulfurirhabdus autotrophica BiS0	LC124209	LC124215
Sulfuriferula multivorans TTN	LC124210	LC124216
Sulfurisoma sediminicola BSN1	LC124211	LC124217

Table S4. Putative phylogenetic affiliations of all OTUs, Putative taxonomic affiliations of environmentally derived *aprA* sequences, inferred from the AprBA consensus tree and the partial AprA tree. The AprBA tree-based inference was performed by using the consensus tree constructed with the sequence set established in this study, whereas the partial AprA tree-based analysis was performed with more conventional methods (see text for details). The differences in results of 2 methods are highlighted by red color. Related organisms of putative affiliation from AprBA consensus tree correspond to those in Figure 2.

OTU	Accession for representative clone	Clone no.	Putative affiliation based on AprBA consensus tree		Putative affiliation based on partial AprA tree	
			Major cluster	Related organisms	Major cluster	Related organisms
04B-OTU1	LC124225	1	Lineage II	Thiobacillus	Lineage II	unknown
04B-OTU2	LC124259	1	Lineage II	Sulfuritalea	Lineage II	suspicious Thiobacillus clone
04B-OTU3	LC124261	4	Desulfobacteraceae	distantly related to Desulfatirhabdium	Desulfobacteraceae	distantly related to Desulfatirhabdium
04B-OTU4	LC124238	1	Desulfobacteraceae	Miscellaneous group 2	Desulfobacteraceae	Desulfofaba
04B-OTU5	LC124270	1	Desulfobacteraceae	Miscellaneous group 1	Desulfobacteraceae	unknown
04B-OTU6	LC124249	1	Desulfobacteraceae	Desulfatitalea	Desulfobacteraceae	distantly related to Desulfatitalea
04B-OTU7	LC124240	1	Desulfobacteraceae	Miscellaneous group 1	Desulfobacteraceae	unknown
04B-OTU8	LC124268	1	Desulfobulbaceae	Desulfobulbus	Desulfobulbaceae	unknown
04B-OTU9	LC124246	12	Lineage II	Candidatus Magnetobacterium	Thermodesulfovibrio	distantly related to Thermodesulfovibrio
04B-OTU10	LC124267	6	Lineage II	Candidatus Magnetobacterium	Thermodesulfovibrio	distantly related to Thermodesulfovibrio
04B-OTU11	LC124227	1	Lineage II	Candidatus Magnetobacterium	Thermodesulfovibrio	distantly related to Thermodesulfovibrio
04B-OTU12	LC124262	9	Thermodesulfovibrio	Thermodesulfovibrio	Thermodesulfovibrio	distantly related to Thermodesulfovibrio

04B-OTU13	LC124231	10	Lineage II	Candidatus Magnetobacterium	Thermodesulfovibrio	distantly related to Thermodesulfovibrio
04B-OTU14	LC124223	1	Lineage II	Candidatus Magnetobacterium	Thermodesulfovibrio	distantly related to Thermodesulfovibrio
04B-OTU15	LC124279	4	Lineage II	Candidatus Magnetobacterium	Thermodesulfovibrio	distantly related to Thermodesulfovibrio
04B-OTU16	LC124250	1	Lineage II	Candidatus Magnetobacterium	Thermodesulfovibrio	distantly related to Thermodesulfovibrio
04B-OTU17	LC124282	2	Thermodesulfovibrio	Thermodesulfovibrio	Thermodesulfovibrio	distantly related to Thermodesulfovibrio
04B-OTU18	LC124276	1	Thermodesulfovibrio	Thermodesulfovibrio	Thermodesulfovibrio	distantly related to Thermodesulfovibrio
04B-OTU19	LC124230	1	Gram-positive & LGT-affected deltaproteobacteria	Delta proteobacterium NaphS2	Gram-positive & LGT-affected deltaproteobacteria	unknown
04B-OTU20	LC124254	2	Lineage I	Sulfuricaulis	Lineage I	Sulfuricaulis
FTR50-OTU1	LC124326	67	Lineage I	Agg47 gammaproteobacteria	Lineage I	unknown
FTR70-OTU1	LC124376	2	Desulfobulbaceae	Desulfobulbus	Desulfobulbaceae	unknown
FTR70-OTU2	LC124359	1	Desulfobacteraceae	distantly related to Desulfatirhabdium	Desulfobacteraceae	distantly related to Desulfatirhabdium
FTR70-OTU3	LC124360	1	Desulfovibrionales	Desulfomicrobium	Thermodesulfovibrio	distantly related to Thermodesulfovibrio
FTR70-OTU4	LC124369	2	Lineage II	Sulfuritalea	Lineage II	distantly related to Sulfuritalea
FTR70-OTU5	LC124363	4	Lineage II	Miscellaneous group 4	Lineage II	Miscellaneous group 4
FTR70-OTU6	LC124371	1	Lineage II	Sulfuricaulis	Lineage II	distantly related to Thioalkalivibrio
FTR70-OTU7	LC124384	1	Lineage II	Candidatus Riegeria	Lineage II	unknown
FTR70-OTU8	LC124387	4	Gram-positive & LGT-affected deltaproteobacteria	Delta proteobacterium NaphS2	Gram-positive & LGT-affected deltaproteobacteria	unknown
FTR70-OTU9	LC124389	13	Lineage II	Candidatus Magnetobacterium	Thermodesulfovibrio	distantly related to Thermodesulfovibrio
FTR70-OTU10	LC124392	2	Lineage II	Candidatus Magnetobacterium	Thermodesulfovibrio	distantly related to Thermodesulfovibrio

FTR70-OTU11	LC124368	5	Lineage II	Candidatus Magnetobacterium	Thermodesulfovibrio	distantly related to Thermodesulfovibrio
FTR70-OTU12	LC124364	1	Lineage II	Candidatus Magnetobacterium	Thermodesulfovibrio	distantly related to Thermodesulfovibrio
FTR70-OTU13	LC124365	3	Lineage I	Halochromatium	Lineage I	Sulfuricaulis
FTR70-OTU14	LC124355	1	Lineage I	Sulfur-oxidizing bacterium DIII5	Lineage I	Sulfuricaulis
FTR70-OTU15	LC124375	1	Lineage I	Sulfuricaulis	Lineage I	Sulfuricaulis
FTR70-OTU16	LC124367	1	Lineage I	Agg47 gammaproteobacteria	Lineage I	unknown
FTR90-OTU1	LC124397	3	Gram-positive & LGT-affected deltaproteobacteria	Desulfomonile	Gram-positive & LGT-affected deltaproteobacteria	Desulfomonile
FTR90-OTU2	LC124411	1	Gram-positive & LGT-affected deltaproteobacteria	Desulfomonile	Gram-positive & LGT-affected deltaproteobacteria	Desulfomonile
FTR90-OTU3	LC124394	5	Gram-positive & LGT-affected deltaproteobacteria	distantly related to Desulfatiglans	Gram-positive & LGT-affected deltaproteobacteria	distantly related to Desulfarculus
FTR90-OTU4	LC124422	6	Gram-positive & LGT-affected deltaproteobacteria	distantly related to Desulfatiglans	Gram-positive & LGT-affected deltaproteobacteria	distantly related to Desulfarculus
FTR90-OTU5	LC124430	1	Gram-positive & LGT-affected deltaproteobacteria	Desulfarculus	Gram-positive & LGT-affected deltaproteobacteria	distantly related to Desulfarculus
FTR90-OTU6	LC124427	1	Gram-positive & LGT-affected deltaproteobacteria	Desulfotomaculum	Gram-positive & LGT-affected deltaproteobacteria	unknown
FTR90-OTU7	LC124399	2	Thermodesulfovibrio	Thermodesulfovibrio	Thermodesulfovibrio	distantly related to Thermodesulfovibrio
FTR90-OTU8	LC124423	2	Thermodesulfovibrio	Thermodesulfovibrio	Thermodesulfovibrio	distantly related to Thermodesulfovibrio
FTR90-OTU9	LC124400	1	Lineage II	Candidatus Magnetobacterium	Thermodesulfovibrio	distantly related to Thermodesulfovibrio
FTR90-OTU10	LC124432	3	Desulfobacteraceae	distantly related to	Desulfobacteraceae	distantly related to Desulfatirhabdium

				Desulfatirhabdium		
FTR90-OTU11	LC124418	1	Desulfobacteraceae	distantly related to Desulfatirhabdium	Desulfobacteraceae	distantly related to Desulfatirhabdium
FTR90-OTU12	LC124421	1	Desulfobacteraceae	distantly related to Desulfatirhabdium	Desulfobacteraceae	distantly related to Desulfatirhabdium
FTR90-OTU13	LC124408	1	Desulfobacteraceae	Desulfatitalea	Desulfobacteraceae	distantly related to Desulfatitalea
FTR90-OTU14	LC124425	1	Desulfobacteraceae	Desulfatitalea	Desulfobacteraceae	distantly related to Desulfatitalea
FTR90-OTU15	LC124405	1	Desulfobulbaceae	Mizugaki enrichment phylotype-aprA	Desulfobulbaceae	unknown
FTR90-OTU16	LC124420	1	Desulfobulbaceae	Desulfobulbus	Desulfobulbaceae	unknown
FTR90-OTU17	LC124429	4	Lineage II	Miscellaneous group 4	Lineage II	distantly related to symbiont
FTR90-OTU18	LC124416	1	Lineage II	Miscellaneous group 4	Lineage II	distantly related to symbiont
FTR90-OTU19	LC124403	1	Lineage II	Miscellaneous group 4	Lineage II	distantly related to symbiont
FTR90-OTU20	LC124413	1	Lineage II	Sulfuritalea	Lineage II	unknown
FTR90-OTU21	LC124404	1	Desulfovibrionales	Desulfovibrio	Lineage II	unknown
FTR90-OTU22	LC124412	1	Lineage II	Candidatus Riegeria	Lineage II	Candidatus Riegeria
FTR90-OTU23	LC124414	1	Desulfobacca	Desulfobacca	Desulfobacca	Desulfobacca
FTR100-OTU1	LC124465	4	Lineage II	Candidatus Magnetobacterium	Thermodesulfovibrio	distantly related to Thermodesulfovibrio
FTR100-OTU2	LC124470	1	Lineage II	Candidatus Magnetobacterium	Thermodesulfovibrio	distantly related to Thermodesulfovibrio
FTR100-OTU3	LC124473	1	Lineage II	Sulfuritalea	Lineage II	Sulfurisoma/ suspicious Thiobacillus clone
FTR100-OTU4	LC124488	1	Lineage II	Sulfurisoma	Lineage II	distantly related to Sulfurisoma
FTR100-OTU5	LC124454	1	Lineage II	Sulfuritalea	Lineage II	distantly related to Sulfuritalea

FTR100-OTU6	LC124435	1	Lineage II	distantly related to Thiodictyon	Lineage II	distantly related to Thiodictyon
FTR100-OTU7	LC124441	3	Lineage II	Miscellaneous group 4	Lineage II	distantly related to symbiont
FTR100-OTU8	LC124461	3	Lineage II	Beggiatoaceae	Lineage II	unknown
FTR100-OTU9	LC124471	14	Desulfobacteraceae	distantly related to Desulfatirhabdium	Desulfobacteraceae	distantly related to Desulfatirhabdium
FTR100-OTU10	LC124453	1	Desulfobacteraceae	distantly related to Desulfatirhabdium	Desulfobacteraceae	distantly related to Desulfatirhabdium
FTR100-OTU11	LC124492	2	Desulfobacteraceae	Desulfatitalea	Desulfobacteraceae	distantly related to Desulfatitalea
FTR100-OTU12	LC124466	1	Desulfobacteraceae	Miscellaneous group 1	Desulfobacteraceae	distantly related to Desulfatitalea
FTR100-OTU13	LC124456	5	Desulfobulbaceae	Mizugaki enrichment phylotype-aprA	Desulfobulbaceae	unknown
FTR100-OTU14	LC124460	1	Desulfobulbaceae	Mizugaki enrichment phylotype-aprA	Desulfobulbaceae	distantly related to Mizugaki enrichment phylotype-aprA and Desulfurivibrio
FTR100-OTU15	LC124446	1	Desulfobulbaceae	Desulfobulbus	Desulfobulbaceae	distantly related to Desulfobulbus
FTR100-OTU16	LC124493	1	Desulfobulbaceae	Desulfobulbus	Desulfobulbaceae	distantly related to Desulfobulbus
FTR100-OTU17	LC124475	3	Desulfobacca	Desulfobacca	Desulfobacca	Desulfobacca
FTR100-OTU18	LC124442	2	Gram-positive & LGT-affected deltaproteobacteria	distantly related to Desulfatiglans	Gram-positive & LGT-affected deltaproteobacteria	distantly related to Desulfarculus
FTR100-OTU19	LC124472	6	Gram-positive & LGT-affected deltaproteobacteria	distantly related to Desulfatiglans	Gram-positive & LGT-affected deltaproteobacteria	distantly related to Desulfarculus
FTR100-OTU20	LC124476	3	Gram-positive & LGT-affected deltaproteobacteria	distantly related to Desulfatiglans	Gram-positive & LGT-affected deltaproteobacteria	distantly related to Desulfarculus

FTR100-OTU21	LC124464	1	Gram-positive & LGT-affected deltaproteobacteria	distantly related to Desulfatiglans	Gram-positive & LGT-affected deltaproteobacteria	distantly related to Desulfarculus
FTR100-OTU22	LC124459	1	Gram-positive & LGT-affected deltaproteobacteria	distantly related to Desulfatiglans	Gram-positive & LGT-affected deltaproteobacteria	distantly related to Desulfarculus
FTR100-OTU23	LC124462	1	Gram-positive & LGT-affected deltaproteobacteria	Delta proteobacterium SM-66-47 and SM-66-64	Gram-positive & LGT-affected deltaproteobacteria	Delta proteobacterium SM-66-47
FTR100-OTU24	LC124483	2	Gram-positive & LGT-affected deltaproteobacteria	Desulfomonile	Gram-positive & LGT-affected deltaproteobacteria	Desulfomonile
FTR100-OTU25	LC124489	1	Gram-positive & LGT-affected deltaproteobacteria	Desulfomonile	Gram-positive & LGT-affected deltaproteobacteria	Desulfomonile
10B-OTU1	AB749927	1	Gram-positive & LGT-affected deltaproteobacteria	Desulfomonile	Gram-positive & LGT-affected deltaproteobacteria	Desulfomonile
10B-OTU2	AB749928	1	Gram-positive & LGT-affected deltaproteobacteria	Desulfotomaculum	Gram-positive & LGT-affected deltaproteobacteria	unknown
10B-OTU3	AB749926	1	Gram-positive & LGT-affected deltaproteobacteria	Delta proteobacterium NaphS2	Gram-positive & LGT-affected deltaproteobacteria	distantly related to Delta proteobacterium NaphS2 and other unclassified spp.
10B-OTU4	AB749961	8	Lineage II	Candidatus Magnetobacterium	Thermodesulfovibrio	distantly related to Thermodesulfovibrio
10B-OTU5	AB749956	1	Lineage II	Candidatus Magnetobacterium	Thermodesulfovibrio	distantly related to Thermodesulfovibrio
10B-OTU6	AB749955	2	Lineage II	Candidatus Magnetobacterium	Thermodesulfovibrio	distantly related to Thermodesulfovibrio
10B-OTU7	AB749953	1	Lineage II	Candidatus Magnetobacterium	Thermodesulfovibrio	distantly related to Thermodesulfovibrio
10B-OTU8	AB749966	2	Lineage II	Candidatus Magnetobacterium	Thermodesulfovibrio	distantly related to Thermodesulfovibrio
10B-OTU9	AB749967	4	Lineage II	Candidatus Magnetobacterium	Thermodesulfovibrio	distantly related to Thermodesulfovibrio

10B-OTU10	AB749974	5	Lineage II	Candidatus Magnetobacterium	Thermodesulfovibrio	distantly related to Thermodesulfovibrio
10B-OTU11	AB749971	2	Lineage II	Candidatus Magnetobacterium	Thermodesulfovibrio	distantly related to Thermodesulfovibrio
10B-OTU12	AB749938	8	Desulfobacteraceae	distantly related to Desulfatirhabdium	Desulfobacteraceae	distantly related to Desulfatirhabdium
10B-OTU13	AB749930	1	Desulfobacteraceae	distantly related to Desulfatirhabdium	Desulfobacteraceae	distantly related to Desulfatirhabdium
10B-OTU14	AB749929	1	Desulfobacteraceae	Desulfofrigus	Desulfobacteraceae	Desulfofaba
10B-OTU15	AB749940	2	Desulfobacteraceae	Desulfatitalea	Desulfobacteraceae	distantly related to Desulfatitalea
10B-OTU16	AB749942	1	Desulfobacteraceae	Miscellaneous group 1	Desulfobacteraceae	unknown
10B-OTU17	AB749941	1	Desulfobacteraceae	Miscellaneous group 1	Desulfobacteraceae	unknown
10B-OTU18	AB749945	2	Desulfobulbaceae	Mizugaki enrichment phylotype-aprA	Desulfobulbaceae	unknown
10B-OTU19	AB749943	1	Desulfobulbaceae	Mizugaki enrichment phylotype-aprA	Desulfobulbaceae	unknown
10B-OTU20	AB749946	3	Lineage II	Sulfuriferula	Lineage II	unknown
10B-OTU21	AB749949	1	Lineage II	distantly related to Thiodictyon	Lineage II	distantly related to Thiodictyon
10B-OTU22	AB749950	1	Lineage II	distantly related to Thiodictyon	Lineage II	distantly related to Thiodictyon
10B-OTU23	AB749951	1	Lineage II	Thiobacillus	Lineage II	unknown
10B-OTU24	AB749952	1	Desulfobacca	Desulfobacca	Desulfobacca	Desulfobacca
10B-OTU25	AB749979	2	Lineage I	Sulfuricaulis	Lineage I	Sulfuricaulis
10B-OTU26	AB749982	2	Lineage I	Sulfuricaulis	Lineage I	Sulfuricaulis
10B-OTU27	AB749980	1	Lineage I	Sulfuricaulis	Lineage I	Sulfuricaulis

Oko-OTU1	AB749985	2	Gram-positive & LGT-affected deltaproteobacteria	Desulfotomaculum	Gram-positive & LGT-affected deltaproteobacteria	unknown
Oko-OTU2	AB749986	1	Gram-positive & LGT-affected deltaproteobacteria	Environmental clone (fosws39f7)	Gram-positive & LGT-affected deltaproteobacteria	unknown
Oko-OTU3	AB749983	1	Gram-positive & LGT-affected deltaproteobacteria	Desulfomonile	Gram-positive & LGT-affected deltaproteobacteria	Desulfomonile
Oko-OTU4	AB749991	6	Desulfobacteraceae	distantly related to Desulfatirhabdium	Desulfobacteraceae	distantly related to Desulfatirhabdium
Oko-OTU5	AB749988	2	Desulfobacteraceae	Desulfosarcina	Desulfobacteraceae	Desulfofaba
Oko-OTU6	AB749990	2	Desulfobacteraceae	Desulfosarcina	Desulfobacteraceae	Desulfofaba
Oko-OTU7	AB749997	1	Desulfobacteraceae	Desulfatitalea	Desulfobacteraceae	distantly related to Desulfatitalea
Oko-OTU8	AB749998	1	Desulfobacteraceae	Desulfatitalea	Desulfobacteraceae	distantly related to Desulfatitalea
Oko-OTU9	AB750000	2	Desulfobacteraceae	Miscellaneous group 1	Desulfobacteraceae	unknown
Oko-OTU10	AB750003	2	Desulfobulbaceae	Mizugaki enrichment phylotype-aprA	Desulfobulbaceae	Mizugaki enrichment phylotype-aprA and Desulfurivibrio
Oko-OTU11	AB750001	1	Desulfobulbaceae	Desulfocapsa	Desulfobulbaceae	distantly related to Desulfocapsa
Oko-OTU12	AB750006	2	Desulfobulbaceae	Desulforhopalus	Desulfobulbaceae	unknown
Oko-OTU13	AB750004	1	Desulfobulbaceae	Desulfobulbus	Desulfobulbaceae	unknown
Oko-OTU14	AB750007	1	Desulfovibrionales	Desulfovibrio	unknown	unknown
Oko-OTU15	AB750009	1	Lineage II	Sulfuritalea	Lineage II	distantly related to Sulfuritalea
Oko-OTU16	AB750010	1	Lineage II	Sulfuricaulis	Lineage II	distantly related to Thioalkalivibrio
Oko-OTU17	AB750008	1	Lineage II	Sulfuriferula	Lineage II	unknown

Oko-OTU18	AB750012	6	Lineage II	Candidatus Magnetobacterium	Thermodesulfovibrio	distantly related to Thermodesulfovibrio
Oko-OTU19	AB750013	1	Lineage II	Candidatus Magnetobacterium	Thermodesulfovibrio	distantly related to Thermodesulfovibrio
Oko-OTU20	AB750011	1	Lineage II	Candidatus Magnetobacterium	Thermodesulfovibrio	distantly related to Thermodesulfovibrio
Oko-OTU21	AB750020	2	Lineage I	Sulfuricaulis	Lineage I	Sulfuricaulis
Oko-OTU22	AB750021	1	Lineage I	Sulfuricaulis	Lineage I	Sulfuricaulis
Oya-OTU1	AB750077	2	Gram-positive & LGT-affected deltaproteobacteria	distantly related to Desulfatiglans	Gram-positive & LGT-affected deltaproteobacteria	distantly related to Desulfarculus
Oya-OTU2	AB750079	2	Gram-positive & LGT-affected deltaproteobacteria	Ammonifex	Gram-positive & LGT-affected deltaproteobacteria	unknown
Oya-OTU3	AB750096	11	Desulfobacteraceae	Miscellaneous group 1	Desulfobacteraceae	unknown
Oya-OTU4	AB750082	3	Desulfobacteraceae	Miscellaneous group 1	Desulfobacteraceae	unknown
Oya-OTU5	AB750080	1	Desulfobacteraceae	Miscellaneous group 1	Desulfobacteraceae	unknown
Oya-OTU6	AB750092	9	Desulfobacteraceae	distantly related to Desulfatirhabdium	Desulfobacteraceae	distantly related to Desulfatirhabdium
Oya-OTU7	AB750104	3	Desulfobulbaceae	Desulfobulbus	Desulfobulbaceae	distantly related to Desulfobulbus
Oya-OTU8	AB750107	5	Desulfobulbaceae	Desulforhopalus	Desulfobulbaceae	distantly related to Desulfotalea
Oya-OTU9	AB750112	1	Desulfobulbaceae	Desulforhopalus	Desulfobulbaceae	distantly related to Desulfotalea
Oya-OTU10	AB750113	1	Lineage II	Sulfuriferula	Lineage II	unknown
Oya-OTU11	AB750115	3	Lineage II	Sulfuriferula	Lineage II	unknown
Oya-OTU12	AB750117	4	Lineage II	Sulfuricella	Lineage II	Sulfuricella
Oya-OTU13	AB750121	8	Lineage II	Sulfuriferula	Lineage II	unknown
Oya-OTU14	AB750131	2	Lineage II	Miscellaneous group 4	Lineage II	Thiobacillus

Oya-OTU15	AB750129	1	Lineage II	Miscellaneous group 4	Lineage II	unknown
Oya-OTU16	AB750137	1	Lineage II	distantly related to Thiodictyon	Lineage II	distantly related to Thiodictyon
Oya-OTU17	AB750133	4	Lineage II	Miscellaneous group 4	Lineage II	distantly related to symbiont
Oya-OTU18	AB750132	1	Lineage II	Miscellaneous group 4	Lineage II	distantly related to symbiont
Oya-OTU19	AB750139	19	Lineage II	Candidatus Magnetobacterium	Thermodesulfovibrio	distantly related to Thermodesulfovibrio
Oya-OTU20	AB750157	1	Lineage I	Sulfuricaulis	Lineage I	Sideroxydans
Oya-OTU21	AB750160	1	Lineage I	Sulfuricaulis	Lineage I	Sulfuricaulis
Oya-OTU22	AB750159	1	Lineage I	Sulfuricaulis	Lineage I	Sulfuricaulis
Oya-OTU23	AB750158	1	Lineage I	Thiobacillus	Lineage I	Thiobacillus
Oya-OTU24	AB750161	3	Lineage I	Sulfuricaulis	Lineage I	Sulfuricaulis
Oya-OTU25	AB750173	10	Lineage I	Sulfuricaulis	Lineage I	Sulfuricaulis
Oya-OTU26	AB750164	1	Lineage I	Sulfuricaulis	Lineage I	Sulfuricaulis
Oya-OTU27	AB750175	1	Lineage I	Sulfurifustis	Lineage I	Sulfurifustis
Ska-OTU1	AB750022	7	Desulfobacteraceae	Miscellaneous group 1	Desulfobacteraceae	unknown
Ska-OTU2	AB750029	9	Desulfobulbaceae	Desulfocapsa	Desulfobulbaceae	distantly related to Desulfocapsa
Ska-OTU3	AB750038	1	Desulfobulbaceae	Desulfobulbus	Desulfobulbaceae	distantly related to Desulfobulbus
Ska-OTU4	AB750039	1	Desulfovibrionales	Desulfovibrio	Desulfovibrionales	Desulfovibrio
Ska-OTU5	AB750042	3	Lineage II	Sulfuriferula	Lineage II	unknown
Ska-OTU6	AB750045	3	Lineage II	distantly related to Thiodictyon	Lineage II	Thiodictyon
Ska-OTU7	AB750043	1	Lineage II	Miscellaneous group 4	Lineage II	distantly related to symbiont
Ska-OTU8	AB750049	3	Desulfobacca	Desulfobacca	Desulfobacca	Desulfobacca
Ska-OTU9	AB750051	23	Lineage II	Candidatus Magnetobacterium	Thermodesulfovibrio	distantly related to Thermodesulfovibrio

Ska-OTU10	AB750075	3	Lineage I	Sulfuricaulis	Lineage I	Sulfuricaulis
MW-OTU1	AB750219	9	Lineage I	Agg47 gammaproteobacteria	Lineage I	unknown
MW-OTU2	AB750228	1	Lineage I	Alpha/Gammaproteobacteria	Lineage I	unknown
MW-OTU3	AB750229	1	Lineage I	Alphaproteobacteria	Lineage I	unknown
MW-OTU4	AB750190	5	Lineage I	Sulfur-oxidizing bacterium DIII5	Lineage I	Sulfuricaulis
MW-OTU5	AB750203	5	Lineage I	Sulfuricaulis	Lineage I	Sulfuricaulis
MW-OTU6	AB750211	7	Lineage I	Sulfuricaulis	Lineage I	Sulfuricaulis
MW-OTU7	AB750202	2	Lineage I	Sulfur-oxidizing bacterium DIII5	Lineage I	Sulfuricaulis
MW-OTU8	AB750215	4	Lineage I	Sulfuricaulis	Lineage I	Sulfuricaulis
MW-OTU9	AB750288	6	Lineage I	Sulfuricaulis	Lineage I	Sulfuricaulis
MW-OTU10	AB750200	7	Lineage I	Sulfuricaulis	Lineage I	Sulfuricaulis
MW-OTU11	AB750260	38	Lineage I	Sulfuricaulis	Lineage I	Sulfuricaulis
MW-OTU12	AB750188	1	Lineage I	Sulfuricaulis	Lineage I	Sulfuricaulis
MW-OTU13	AB750178	2	Desulfobulbaceae	Desulfobulbus	Desulfobulbaceae	unknown
MW-OTU14	AB750179	1	Desulfobulbaceae	Desulforhopalus	Desulfobulbaceae	distantly related to Desulfotalea
MW-OTU15	AB750176	1	Desulfobacteraceae	distantly related to Desulfatirhabdium	Desulfobacteraceae	distantly related to Desulfatirhabdium
MW-OTU16	AB750230	19	Lineage II	Sulfuricella	Lineage II	Sulfuricella
MW-OTU17	AB750180	1	Lineage II	Miscellaneous group 4	Lineage II	unknown
MW-OTU18	AB750249	1	Lineage II	Sulfuritalea	Lineage II	distantly related to Sulfuritalea
MW-OTU19	AB750250	1	Lineage II	Sulfurifustis	Lineage II	Sulfurifustis
MW-OTU20	AB750251	1	Thermodesulfovibrio	Thermodesulfovibrio	Lineage II (probably)	unknown

MW-OTU21	AB750252	4	Thermodesulfovibrio	Thermodesulfovibrio	Lineage II (probably)	unknown
MW-OTU22	AB750293	1	Archaeoglobus	Archaeoglobus	Euryarchaeota	distantly related to Archaeoglobus
Miz-OTU1	AB754200	11	Lineage I	Sulfur-oxidizing bacterium DIII5	Lineage I	Sulfuricaulis
Miz-OTU2	AB754336	3	Lineage I	Sulfuricaulis	Lineage I	Sulfuricaulis
Miz-OTU3	AB754246	1	Lineage I	Sulfuricaulis	Lineage I	Sulfuricaulis
Miz-OTU4	AB754234	2	Lineage II	Candidatus Magnetobacterium	Thermodesulfovibrio	distantly related to Thermodesulfovibrio
Miz-OTU5	AB754267	1	Thermodesulfovibrio	Thermodesulfovibrio	Thermodesulfovibrio	distantly related to Thermodesulfovibrio
Miz-OTU6	AB754328	1	Thermodesulfovibrio	Thermodesulfovibrio	Thermodesulfovibrio	distantly related to Thermodesulfovibrio
Miz-OTU7	AB754231	4	Lineage II	Sulfuritalea	Lineage II	distantly related to Sulfuritalea
Miz-OTU8	AB754213	11	Lineage II	Sulfuritalea	Lineage II	Sulfuritalea
Miz-OTU9	AB754274	2	Lineage II	distantly related to Thiodictyon	Lineage II	distantly related to Thiodictyon
Miz-OTU10	AB754245	1	Lineage II	distantly related to Thiodictyon	Lineage II	distantly related to Thiodictyon
Miz-OTU11	AB754258	2	Lineage II	distantly related to Thiodictyon	Lineage II	distantly related to Thiodictyon
Miz-OTU12	AB754254	2	Lineage II	Sulfuritalea	Lineage II	distantly related to Thiodictyon
Miz-OTU13	AB754334	2	Lineage II	distantly related to Thiodictyon	Lineage II	distantly related to Thiodictyon
Miz-OTU14	AB754237	2	Lineage II	distantly related to Thiodictyon	Lineage II	distantly related to Thiodictyon
Miz-OTU15	AB754293	1	Lineage II	Thiobacillus	Lineage II	unknown
Miz-OTU16	AB754259	1	Lineage II	Thiobacillus	Lineage II	unknown
Miz-OTU17	AB754284	1	Desulfovibrionales	Desulfovibrio	Lineage II	unknown
Miz-OTU18	AB754229	3	Desulfobulbaceae	Desulfobulbus	Desulfobulbaceae	Desulfobulbus
Miz-OTU19	AB754207	4	Desulfobulbaceae	Desulfobulbus	Desulfobulbaceae	Desulfobulbus
Miz-OTU20	AB754196	1	Desulfobulbaceae	Desulfobulbus	Desulfobulbaceae	Desulfobulbus

Miz-OTU21	AB754312	15	Desulfobulbaceae	Desulfobulbus	Desulfobulbaceae	Desulfobulbus
Miz-OTU22	AB754288	1	Desulfobulbaceae	Desulfobulbus	Desulfobulbaceae	Desulfobulbus
Miz-OTU23	AB754263	1	Desulfobulbaceae	Desulfobulbus	Desulfobulbaceae	Desulfobulbus
Miz-OTU24	AB754281	8	Desulfobulbaceae	Mizugaki enrichment phylotype-aprA	Desulfobulbaceae	distantly related to Mizugaki enrichment phylotype-aprA and Desulfurivibrio
Miz-OTU25	AB754291	1	Desulfobulbaceae	Mizugaki enrichment phylotype-aprA	Desulfobulbaceae	distantly related to Mizugaki enrichment phylotype-aprA and Desulfurivibrio
Miz-OTU26	AB754310	1	Desulfobulbaceae	Mizugaki enrichment phylotype-aprA	Desulfobulbaceae	distantly related to Mizugaki enrichment phylotype-aprA and Desulfurivibrio
Miz-OTU27	AB754260	1	Desulfobulbaceae	Miscellaneous group 3	Desulfobulbaceae	unknown
Miz-OTU28	AB754305	46	Desulfobacteraceae	distantly related to Desulfatirhabdium	Desulfobacteraceae	distantly related to Desulfatirhabdium
Miz-OTU29	AB754195	1	Desulfobacteraceae	distantly related to Desulfatirhabdium	Desulfobacteraceae	distantly related to Desulfatirhabdium
Miz-OTU30	AB754255	1	Desulfobacteraceae	Miscellaneous group 1	Desulfobacteraceae	unknown
Miz-OTU31	AB754227	19	Lineage I	Alpha/Gammaproteobacteria	Lineage I	unknown
Miz-OTU32	AB754226	1	Lineage I	Alpha/Gammaproteobacteria	Lineage I	unknown
Whillans-OTU1	KM590034	28	Thermodesulfovibrio	Thermodesulfovibrio	ND	ND
Whillans-OTU2	KM589972	2	Thermodesulfovibrio	Thermodesulfovibrio	ND	ND
Whillans-OTU3	KM590040	10	Lineage II	Candidatus Magnetobacterium	ND	ND
Whillans-OTU4	KM589998	2	Lineage II	Candidatus Magnetobacterium	ND	ND
Whillans-OTU5	KM589967	5	Lineage II	Candidatus Magnetobacterium	ND	ND

Whillans-OTU6	KM590042	6	Thermodesulfovibrio	Thermodesulfovibrio	ND	ND
Whillans-OTU7	KM590039	8	Thermodesulfovibrio	Thermodesulfovibrio	ND	ND
Whillans-OTU8	KM589951	11	Lineage II	distantly related to Thiodictyon	Lineage II	distantly related to Thiodictyon
Whillans-OTU9	KM589966	1	Lineage II	distantly related to Thiodictyon	Lineage II	distantly related to Thiodictyon
Whillans-OTU10	KM589890	1	Lineage II	distantly related to Thiodictyon	Lineage II	distantly related to Thiodictyon
Whillans-OTU11	KM590098	1	Lineage II	distantly related to Thiodictyon	Lineage II	distantly related to Thiodictyon
Whillans-OTU12	KM589995	1	Lineage II	Sulfurirhabdus	Lineage II	Sulfuricella
Whillans-OTU13	KM590097	1	Lineage II	Sulfuriferula	Lineage II	distantly related to Sulfuritalea
Whillans-OTU14	KM590031	6	Lineage II	Sulfuritalea	Lineage II	unknown
Whillans-OTU15	KM590015	11	Lineage II	Sulfurifustis	Lineage II	Thioalkalivibrio
Whillans-OTU16	KM589928	2	Desulfobacteraceae	Desulfatitalea	Desulfobacteraceae	distantly related to Desulfatitalea
Whillans-OTU17	KM589927	3	Desulfobacteraceae	Desulfatitalea	Desulfobacteraceae	distantly related to Desulfatitalea
Whillans-OTU18	KM590035	1	Lineage I	Agg47 gammaproteobacteria	Lineage I	unknown
Whillans-OTU19	KM590109	1	Lineage I	Agg47 gammaproteobacteria	Lineage I	unknown
Whillans-OTU20	KM589971	1	Lineage I	Agg47 gammaproteobacteria	Lineage I	unknown
Whillans-OTU21	KM589978	5	Lineage I	Sulfuricaulis	Lineage I	Sulfuricaulis
Whillans-OTU22	KM589869	1	Lineage I	Sulfuricaulis	Lineage I	Sulfuricaulis
Whillans-OTU23	KM590116	6	Lineage I	Sulfuricaulis	Lineage I	Sulfuricaulis
Whillans-OTU24	KM590011	2	Lineage I	Sulfuricaulis	Lineage I	Sulfuricaulis
Whillans-OTU25	KM590045	28	Lineage I	Sulfuricaulis	Lineage I	Sulfuricaulis
Whillans-OTU26	KM590102	8	Lineage I	Sulfuricaulis	Lineage I	Sulfuricaulis
Whillans-OTU27	KM590075	19	Lineage I	Sulfuricaulis	Lineage I	Sulfuricaulis

Whillans-OTU28	KM590074	39	Lineage I	Sulfuricaulis	Lineage I	Sulfuricaulis
Whillans-OTU29	KM590037	47	Lineage I	Sulfuricaulis	Lineage I	Sulfuricaulis
Whillans-OTU30	KM589974	1	Lineage I	Sulfuricaulis	Lineage I	Sulfuricaulis
Whillans-OTU31	KM590126	8	Lineage I	Sulfuricaulis	Lineage I	Sulfuricaulis
Whillans-OTU32	KM589894	1	Lineage I	Sulfuricaulis	Lineage I	Sulfuricaulis
Whillans-OTU33	KM590101	2	Lineage I	Sulfuricaulis	Lineage I	Sulfuricaulis
Whillans-OTU34	KM590104	2	Lineage I	Sulfuricaulis	Lineage I	Sulfuricaulis
Whillans-OTU35	KM589911	1	Gram-positive & LGT-affected deltaproteobacteria	Delta proteobacterium NaphS2	Gram-positive & LGT-affected deltaproteobacteria	unknown
Whillans-OTU36	KM590032	1	Gram-positive & LGT-affected deltaproteobacteria	Delta proteobacterium NaphS2	Gram-positive & LGT-affected deltaproteobacteria	unknown
Whillans-OTU37	KM590026	1	Gram-positive & LGT-affected deltaproteobacteria	Delta proteobacterium SM-66-47 and SM-66-64	Gram-positive & LGT-affected deltaproteobacteria	unknown
Whillans-OTU38	KM589921	1	Gram-positive & LGT-affected deltaproteobacteria	Desulfotomaculum	Gram-positive & LGT-affected deltaproteobacteria	unknown

Table S5. Descriptions of 16S rRNA gene clone libraries analyzed in this study.

Clone library	Total clone no.	Unique clone no.	Cut off value	OTU no.	Data source
r-04B	77	69	0.02	60	this study
r-10B	83	78	0.02	69	Reference 3
r-Oko	48	44	0.02	37	this study
r-Miz	256	209	0.02	81	Reference 4
r-FTR90	44	38	0.02	29	Reference 5
r-MW24	56	51	0.01	15	this study
r-Oya	64	59	0.02	46	this study
r-Ska	53	52	0.02	28	this study

Table S6. Representative sequences selected for blastn search.

Accession number	Organism
NZ_ATTF01000016.1	Candidatus Pelagibacter ubique HIMB058
AP014879	Sulfuricaulis limicola HA5
CP007029.1	Thioalkalivibrio paradoxus ARh 1
NZ_KB891317.1	Thiobacillus thioparus DSM 505
CP000852.1	Caldivirga maquilingensis IC-167
CP002590.1	Thermoproteus uzoniensis 768-20
NZ_AEVP00000000.2	Desulfotomaculum nigrificans DSM 574
CP002085.1	Desulfarculus baarsii DSM 2075
CP001785.1	Ammonifex degensii KC4
CP001857.1	Archaeoglobus profundus DSM 5631
NC_002932	Chlorobium tepidum TLS
CP001147.1	Thermodesulfovibrio yellowstonii DSM 11347
NC_007404.1	Thiobacillus denitrificans ATCC 25259
AP012273.1	Thiolapillus brandeum Hiromi 1
AP012547.1	Sulfuritalea hydrogenivorans sk43H
CP003154.1	Thiocystis violascens DSM 198
AP014936	Sulfurifustis variabilis skN76
NZ_JQKX01000002.1	Thermopetrobacter sp. TC1
CP002683.1	Thermodesulfatator indicus DSM 15286
CP002829.1	Thermodesulfobacterium geofontis OPF15
CP001322.1	Desulfatibacillum alkenivorans AK-01
NC_012108.1	Desulfobacterium autotrophicum DSM 3382
CP001940.1	Desulfurivibrio alkaliphilus AHT2
NZ_JPIK01000013.1	Desulfonatronum thiodismutans MLF-1
NZ_AVAG01000038.1	Desulfonauticus sp. A7A
NZ_JYNP01000016.1	Desulfobulbus alkaliphilus APS1
NC_011769.1	Desulfovibrio vulgaris 'Miyazaki F'
NZ_DS996360.1	Desulfovibrio piger ATCC 29098
CP001629.1	Desulfomicrobium baculatum DSM 4028
NC_016629.1	Desulfovibrio africanus Walvis Bay
NC_012796.1	Desulfovibrio magneticus RS-1
NZ_AUMA01000009.1	Desulfovibrio aminophilus DSM 12254

NC_020409.1	Desulfovibrio piezophilus C1TLV30
AM402962.1	Bacterial endosymbiont of Idas sp.
HG513080.1	Bacterium endosymbiont of Phreagena soyoae
EU439004.1	Candidatus Thiobios zoothamnicoli Calvi
FR865040.1	Endosymbiont of Bathymodiolus puteoserpentis
HE680083.1	Gamma proteobacterium symbiont of Isorropodon bigoti
AM503928.1	Mytilidae sp. morphotype BC 1007 gill symbiont
AM234051.1	Olavius algarvensis Gamma 3 endosymbiont
EF641961.1	Thiocapsa rosea DSM 235
EF641929.1	symbiont of Ifremeria nautilei
FN869546.1	Gloverina rectangularis gill endosymbiont
FN869534.1	Myrtea tanimbarensis gill endosymbiont
HQ675107.1	Delta proteobacterium SCGC AAA240-I05
HE575211.1	Delta proteobacterium SM-66-47
AF418120.1	Desulfomicrobium baculatum DSM 1743
AF418135.1	Desulfonema ishimotonii DSM 9680
DQ780255.1	Endoriftia persephone 'Hot96_1+Hot96_2' clone rif_2415_009
FN908119.1	Codakia orbicularis gill endosymbiont
FN908118.1	Anodontia alba gill endosymbiont
AM883193.1	endosymbiont of Oligobrachia haakonmosbiensis
AF418106.1	Desulfovibrio intestinalis DSM 11275
AF418145.1	Desulfofrigus oceanense DSM 12341
AF418118.1	Desulfofaba gelida DSM 12344
EF442974.1	Thermodesulfovibrio sp. (ex DSM 12270)
AF418112.1	Thermodesulfobacterium thermophilum DSM 1276
AF418117.1	Desulfocella halophila DSM 11763
HQ675110.1	Delta proteobacterium SCGC AAA240-J23
HQ675105.1	Delta proteobacterium SCGC AAA240-D19
EF442892.1	Desulfovibrio sp. LB1
EF641914.1	Thiocapsa pendens DSM 236
FN869550.1	Lucinidae gen. sp. TB-2010 gill endosymbiont
AM228902.1	Gamma proteobacterium AB-2006-IL
HE974483.1	Bacterium endosymbiont of Lamellibrachia anaximandri
FR715573.1	Gamma proteobacterium symbiont of Thyasira sp. 'Eastern Mediterranean'
FM165460.1	Lamellibrachia sp. endosymbiont, clone APSV2.2

AM883195.1	endosymbiont of Sclerolinum contortum, clone Scon
EF641962.1	Sulfur-oxidizing symbiont of Oasisa sp.
KC832743.1	Gamma proteobacterium endosymbiont of Lamellibrachia anaximandri type A clone Tw2_31
AF418110.1	Desulfobulbus rhabdoformis DSM 8777
EF442937.1	Desulfobulbus sp. LB2
HE863803.1	Bacterium symbiont of Acharax sp. Guiness
DQ890382.1	Gamma proteobacterium endosymbiont of Astomonema sp.
AB425029.1	Oligobrachia mashikoi endosymbiont A
AF418163.1	Desulforhopalus singaporensis DSM 12130
AB425031.1	Oligobrachia mashikoi endosymbiont C
AF418151.1	Desulfococcus biacutus DSM 5651
AF418107.1	Desulfobacter curvatus DSM 3379
AF418160.1	Desulforhopalus sp. LSv20
AF418124.1	Desulfobacterium vacuolatum DSM 3385
AM234053.1	Olavius algarvensis Delta 4 endosymbiont, clone Oalg8899AB_APS3b
HE575209.1	Delta proteobacterium SM-66-13
BDFE00000000.1	Desulfoplanes formicivorans Pf12B
AB425035.1	Oligobrachia mashikoi endosymbiont G
AB425032.1	Oligobrachia mashikoi endosymbiont D
FR715574.1	Gamma proteobacterium symbiont of Thyasira sp. REGAB
HQ675093.1	Delta proteobacterium SCGC AAA007-D22
HQ675112.1	Delta proteobacterium SCGC AAA240-N20

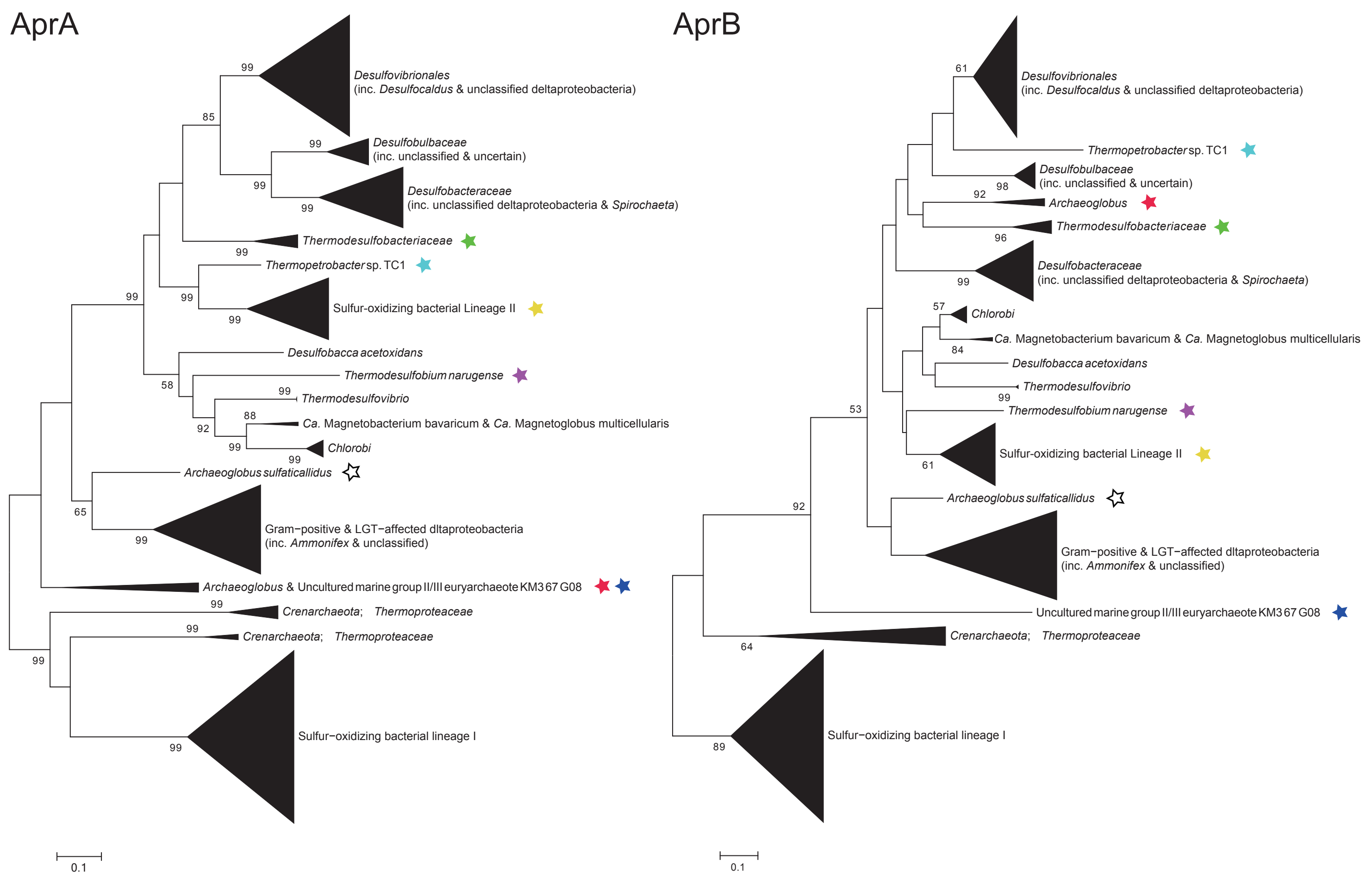

Fig. S1. ML trees based on nearly full-length 320 AprA and AprB sequences in the core sequence set. The trees were inferred using the JTT matrix-based model. Numbers at the nodes indicate confidence values of 1000 bootstrap analysis (values ≥ 50% are shown). There were a total of 503 and 82 comparable sites of AprA and AprB, respectively. The positions of clusters and branches with a star were varied between AprB and AprA trees.

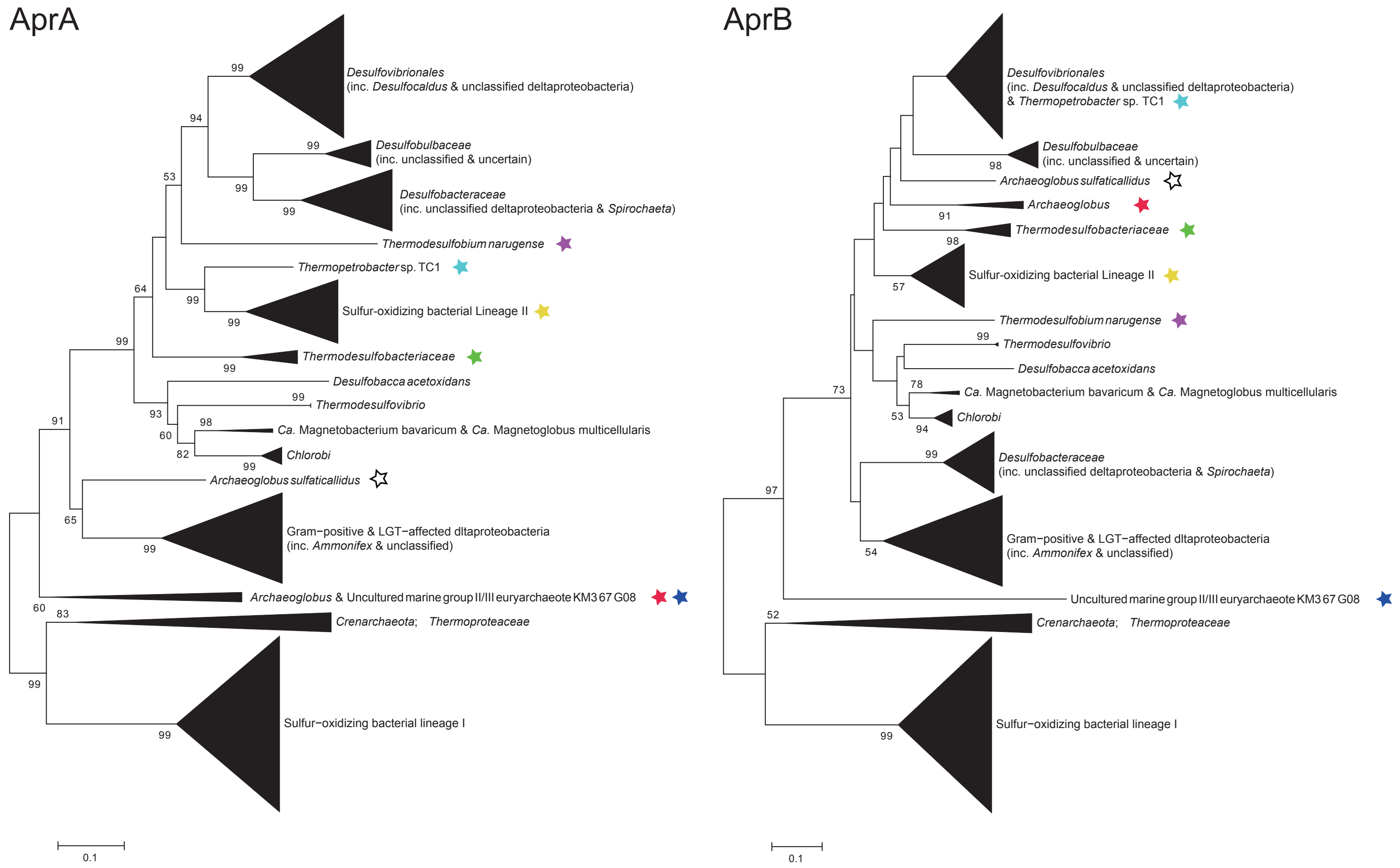

Fig. S2. NJ trees based on nearly full-length 320 AprA and AprB sequences in the core sequence set. The trees were inferred using the JTT matrix-based model. Numbers at the nodes indicate confidence values of 1000 bootstrap analysis (values ≥ 50% are shown). There were a total of 503 and 82 comparable sites of AprA and AprB, respectively. The positions of clusters and branches with a star were varied between AprB and AprA trees.

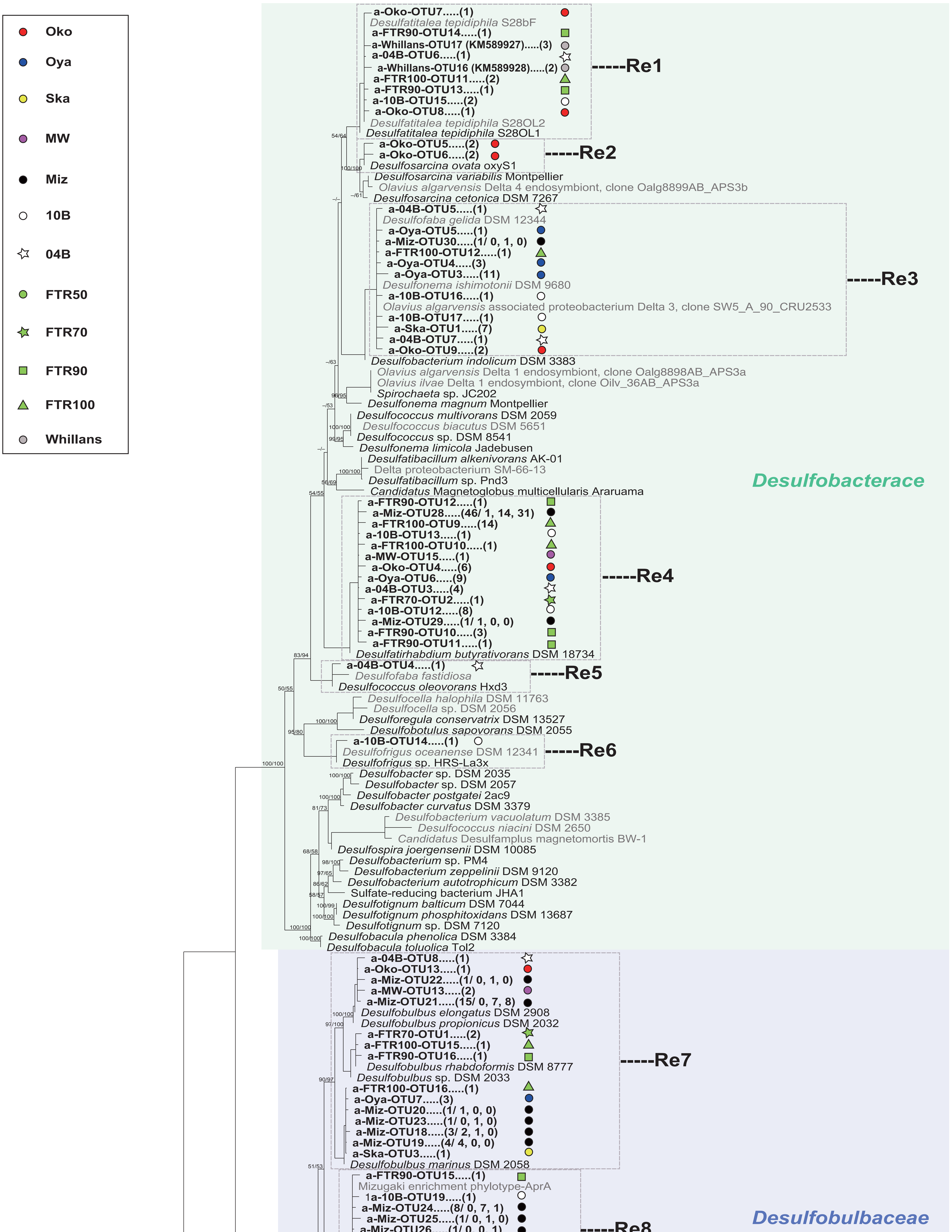
Oko
Oya
Ska
MW
Miz
10B
04B
FTR50
FTR70
FTR90
FTR100
Whillans
a-Oko-OTU7.....(1)
Desulfatitalea tepidiphila S28bF
a-FTR90-OTU14.....(1)
a-Whillans-OTU17 (KM589927).....(3)
a-04B-OTU6.....(1)
a-Whillans-OTU16 (KM589928).....(2)
a-FTR100-OTU11.....(2)
a-FTR90-OTU13.....(1)
a-10B-OTU15.....(2)
a-Oko-OTU8.....(1)
Desulfatitalea tepidiphila S28OL2
Desulfatitalea tepidiphila S28OL1
-----Re1
a-Oko-OTU5.....(2)
a-Oko-OTU6.....(2)
Desulfosarcina ovata oxyS1
-----Re2
Desulfosarcina variabilis Montpellier
Olavius algarvensis Delta 4 endosymbiont, clone Oalg8899AB_APS3b
Desulfosarcina cetonica DSM 7267
a-04B-OTU5.....(1)
Desulfofaba gelida DSM 12344
a-Oya-OTU5.....(1)
a-Miz-OTU30.....(1/ 0, 1, 0)
a-FTR100-OTU12.....(1)
a-Oya-OTU4.....(3)
a-Oya-OTU3.....(11)
Desulfonema ishimotonii DSM 9680
a-10B-OTU16.....(1)
Olavius algarvensis associated proteobacterium Delta 3, clone SW5_A_90_CRU2533
a-10B-OTU17.....(1)
a-Ska-OTU1.....(7)
a-04B-OTU7.....(1)
a-Oko-OTU9.....(2)
-----Re3
Desulfobacterium indolicum DSM 3383
Olavius algarvensis Delta 1 endosymbiont, clone Oalg8898AB_APS3a
Olavius ilvae Delta 1 endosymbiont, clone Oilv_36AB_APS3a
Spirochaeta sp. JC202
Desulfonema magnum Montpellier
Desulfococcus multivorans DSM 2059
Desulfococcus biacutus DSM 5651
Desulfococcus sp. DSM 8541
Desulfonema limicola Jadebusen
Desulfatibacillum alkenivorans AK-01
Delta proteobacterium SM-66-13
Desulfatibacillum sp. Pnd3
Candidatus Magnetoglobus multicellularis Araruama
Desulfobacterace
a-FTR90-OTU12.....(1)
a-Miz-OTU28.....(46/ 1, 14, 31)
a-FTR100-OTU9.....(14)
a-10B-OTU13.....(1)
a-FTR100-OTU10.....(1)
a-MW-OTU15.....(1)
a-Oko-OTU4.....(6)
a-Oya-OTU6.....(9)
a-04B-OTU3.....(4)
a-FTR70-OTU2.....(1)
a-10B-OTU12.....(8)
a-Miz-OTU29.....(1/ 1, 0, 0)
a-FTR90-OTU10.....(3)
a-FTR90-OTU11.....(1)
Desulfatirhabdium butyrativorans DSM 18734
-----Re4
a-04B-OTU4.....(1)
Desulfofaba fastidiosa
Desulfococcus oleovorans Hxd3
-----Re5
Desulfocella halophila DSM 11763
Desulfocella sp. DSM 2056
Desulforegula conservatrix DSM 13527
Desulfobotulus sapovorans DSM 2055
a-10B-OTU14.....(1)
Desulfofrigus oceanense DSM 12341
Desulfofrigus sp. HRS-La3x
-----Re6
Desulfobacter sp. DSM 2035
Desulfobacter sp. DSM 2057
Desulfobacter postgatei 2ac9
Desulfobacter curvatus DSM 3379
Desulfobacterium vacuolatum DSM 3385
Desulfococcus niacini DSM 2650
Candidatus Desulfamplus magnetomortis BW-1
Desulfospira joergensenii DSM 10085
Desulfobacterium sp. PM4
Desulfobacterium zeppelinii DSM 9120
Desulfobacterium autotrophicum DSM 3382
Sulfate-reducing bacterium JHA1
Desulfotignum balticum DSM 7044
Desulfotignum phosphitoxidans DSM 13687
Desulfotignum sp. DSM 7120
Desulfobacula phenolica DSM 3384
Desulfobacula toluolica Tol2
a-04B-OTU8.....(1)
a-Oko-OTU13.....(1)
a-Miz-OTU22.....(1/ 0, 1, 0)
a-MW-OTU13.....(2)
a-Miz-OTU21.....(15/ 0, 7, 8)
Desulfobulbus elongatus DSM 2908
Desulfobulbus propionicus DSM 2032
a-FTR70-OTU1.....(2)
a-FTR100-OTU15.....(1)
a-FTR90-OTU16.....(1)
Desulfobulbus rhabdoformis DSM 8777
Desulfobulbus sp. DSM 2033
a-FTR100-OTU16.....(1)
a-Oya-OTU7.....(3)
a-Miz-OTU20.....(1/ 1, 0, 0)
a-Miz-OTU23.....(1/ 0, 1, 0)
a-Miz-OTU18.....(3/ 2, 1, 0)
a-Miz-OTU19.....(4/ 4, 0, 0)
a-Ska-OTU3.....(1)
Desulfobulbus marinus DSM 2058
-----Re7
a-FTR90-OTU15.....(1)
Mizugaki enrichment phylotype-AprA
1a-10B-OTU19.....(1)
a-Miz-OTU24.....(8/ 0, 7, 1)
a-Miz-OTU25.....(1/ 0, 1, 0)
-----Re8
Desulfobulbaceae

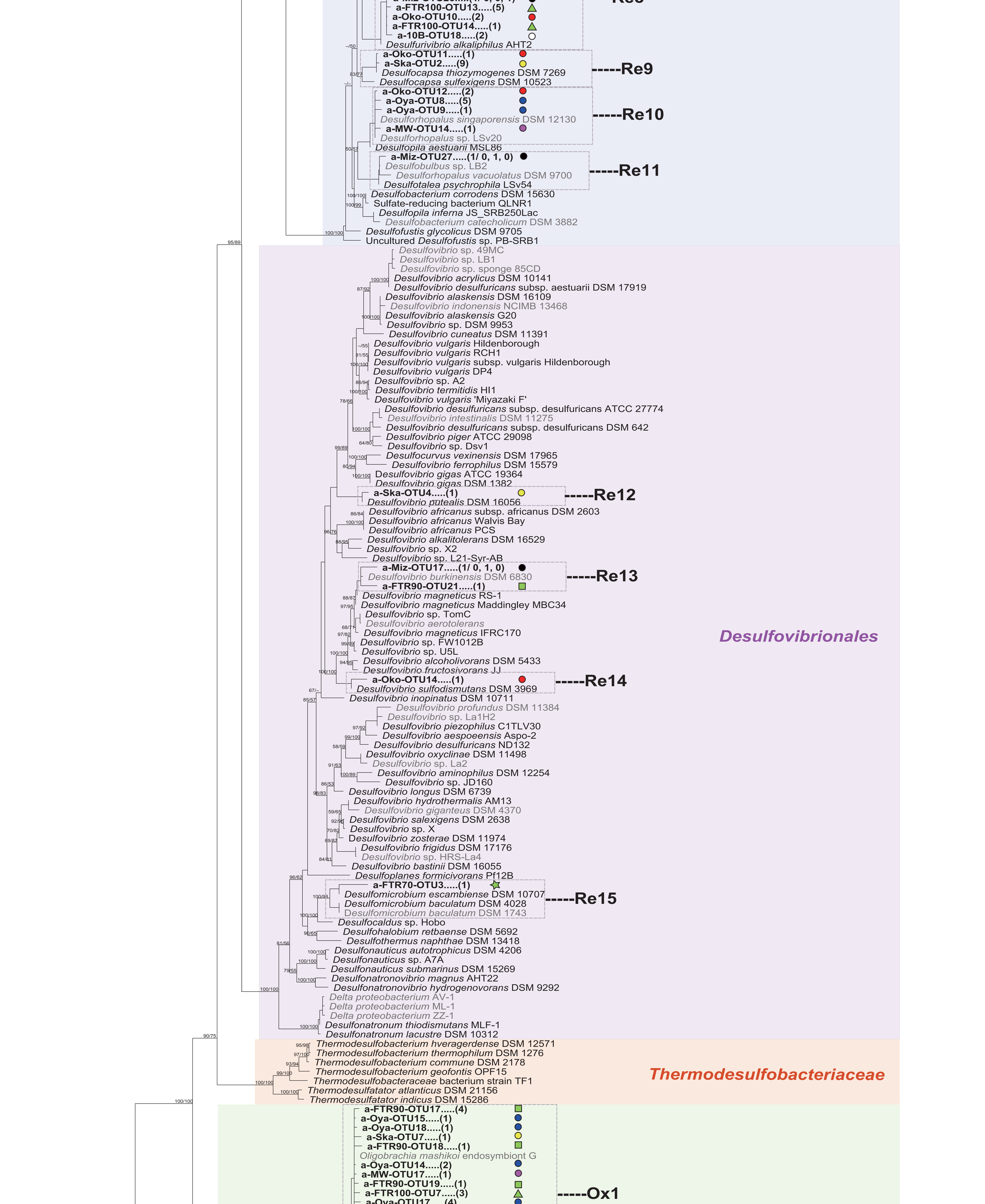

a-FTR100-OTU13.....(5)
a-Oko-OTU10.....(2)
a-FTR100-OTU14.....(1)
a-10B-OTU18.....(2)
Desulfurivibrio alkaliphilus AHT2
a-Oko-OTU11.....(1)
a-Ska-OTU2.....(9)
Desulfocapsa thiozymogenes DSM 7269
Desulfocapsa sulfexigens DSM 10523
-----Re9
a-Oko-OTU12.....(2)
a-Oya-OTU8.....(5)
a-Oya-OTU9.....(1)
Desulforhopalus singaporensis DSM 12130
a-MW-OTU14.....(1)
Desulforhopalus sp. LSv20
-----Re10
Desulfopila aestuarii MSL86
a-Miz-OTU27.....(1/ 0, 1, 0)
Desulfobulbus sp. LB2
Desulforhopalus vacuolatus DSM 9700
Desulfotalea psychrophila LSv54
-----Re11
Desulfobacterium corrodens DSM 15630
Sulfate-reducing bacterium QLNR1
Desulfopila inferna JS_SRB250Lac
Desulfobacterium catecholicum DSM 3882
Desulfofustis glycolicus DSM 9705
Uncultured Desulfofustis sp. PB-SRB1
Desulfovibrio sp. 49MC
Desulfovibrio sp. LB1
Desulfovibrio sp. sponge 85CD
Desulfovibrio acrylicus DSM 10141
Desulfovibrio desulfuricans subsp. aestuarii DSM 17919
Desulfovibrio alaskensis DSM 16109
Desulfovibrio indonensis NCIMB 13468
Desulfovibrio alaskensis G20
Desulfovibrio sp. DSM 9953
Desulfovibrio cuneatus DSM 11391
Desulfovibrio vulgaris Hildenborough
Desulfovibrio vulgaris RCH1
Desulfovibrio vulgaris subsp. vulgaris Hildenborough
Desulfovibrio vulgaris DP4
Desulfovibrio sp. A2
Desulfovibrio termitidis HI1
Desulfovibrio vulgaris 'Miyazaki F'
Desulfovibrio desulfuricans subsp. desulfuricans ATCC 27774
Desulfovibrio intestinalis DSM 11275
Desulfovibrio desulfuricans subsp. desulfuricans DSM 642
Desulfovibrio piger ATCC 29098
Desulfovibrio sp. Dsv1
Desulfocurvus vexinensis DSM 17965
Desulfovibrio ferrophilus DSM 15579
Desulfovibrio gigas ATCC 19364
Desulfovibrio gigas DSM 1382
a-Ska-OTU4.....(1)
Desulfovibrio putealis DSM 16056
-----Re12
Desulfovibrio africanus subsp. africanus DSM 2603
Desulfovibrio africanus Walvis Bay
Desulfovibrio africanus PCS
Desulfovibrio alkalitolerans DSM 16529
Desulfovibrio sp. X2
Desulfovibrio sp. L21-Syr-AB
a-Miz-OTU17.....(1/ 0, 1, 0)
Desulfovibrio burkinensis DSM 6830
a-FTR90-OTU21.....(1)
-----Re13
Desulfovibrio magneticus RS-1
Desulfovibrio magneticus Maddingley MBC34
Desulfovibrio sp. TomC
Desulfovibrio aerotolerans
Desulfovibrio magneticus IFRC170
Desulfovibrio sp. FW1012B
Desulfovibrio sp. U5L
Desulfovibrio alcoholivorans DSM 5433
Desulfovibrio fructosivorans JJ
Desulfovibrionales
a-Oko-OTU14.....(1)
Desulfovibrio sulfodismutans DSM 3969
-----Re14
Desulfovibrio inopinatus DSM 10711
Desulfovibrio profundus DSM 11384
Desulfovibrio sp. La1H2
Desulfovibrio piezophilus C1TLV30
Desulfovibrio aespoeensis Aspo-2
Desulfovibrio desulfuricans ND132
Desulfovibrio oxyclinae DSM 11498
Desulfovibrio sp. La2
Desulfovibrio aminophilus DSM 12254
Desulfovibrio sp. JD160
Desulfovibrio longus DSM 6739
Desulfovibrio hydrothermalis AM13
Desulfovibrio giganteus DSM 4370
Desulfovibrio salexigens DSM 2638
Desulfovibrio sp. X
Desulfovibrio zosterae DSM 11974
Desulfovibrio frigidus DSM 17176
Desulfovibrio sp. HRS-La4
Desulfovibrio bastinii DSM 16055
Desulfoplanes formicivorans Pf12B
a-FTR70-OTU3.....(1)
Desulfomicrobium escambiense DSM 10707
Desulfomicrobium baculatum DSM 4028
Desulfomicrobium baculatum DSM 1743
-----Re15
Desulfocaldus sp. Hobo
Desulfohalobium retbaense DSM 5692
Desulfothermus naphthae DSM 13418
Desulfonauticus autotrophicus DSM 4206
Desulfonauticus sp. A7A
Desulfonauticus submarinus DSM 15269
Desulfonatronovibrio magnus AHT22
Desulfonatronovibrio hydrogenovorans DSM 9292
Delta proteobacterium AV-1
Delta proteobacterium ML-1
Delta proteobacterium ZZ-1
Desulfonatronum thiodismutans MLF-1
Desulfonatronum lacustre DSM 10312
Thermodesulfobacterium hveragerdense DSM 12571
Thermodesulfobacterium thermophilum DSM 1276
Thermodesulfobacterium commune DSM 2178
Thermodesulfobacterium geofontis OPF15
Thermodesulfobacteraceae bacterium strain TF1
Thermodesulfatator atlanticus DSM 21156
Thermodesulfatator indicus DSM 15286
Thermodesulfobacteriaceae
a-FTR90-OTU17.....(4)
a-Oya-OTU15.....(1)
a-Oya-OTU18.....(1)
a-Ska-OTU7.....(1)
a-FTR90-OTU18.....(1)
Oligobrachia mashikoi endosymbiont G
a-Oya-OTU14.....(2)
a-MW-OTU17.....(1)
a-FTR90-OTU19.....(1)
a-FTR100-OTU7.....(3)
-----Ox1

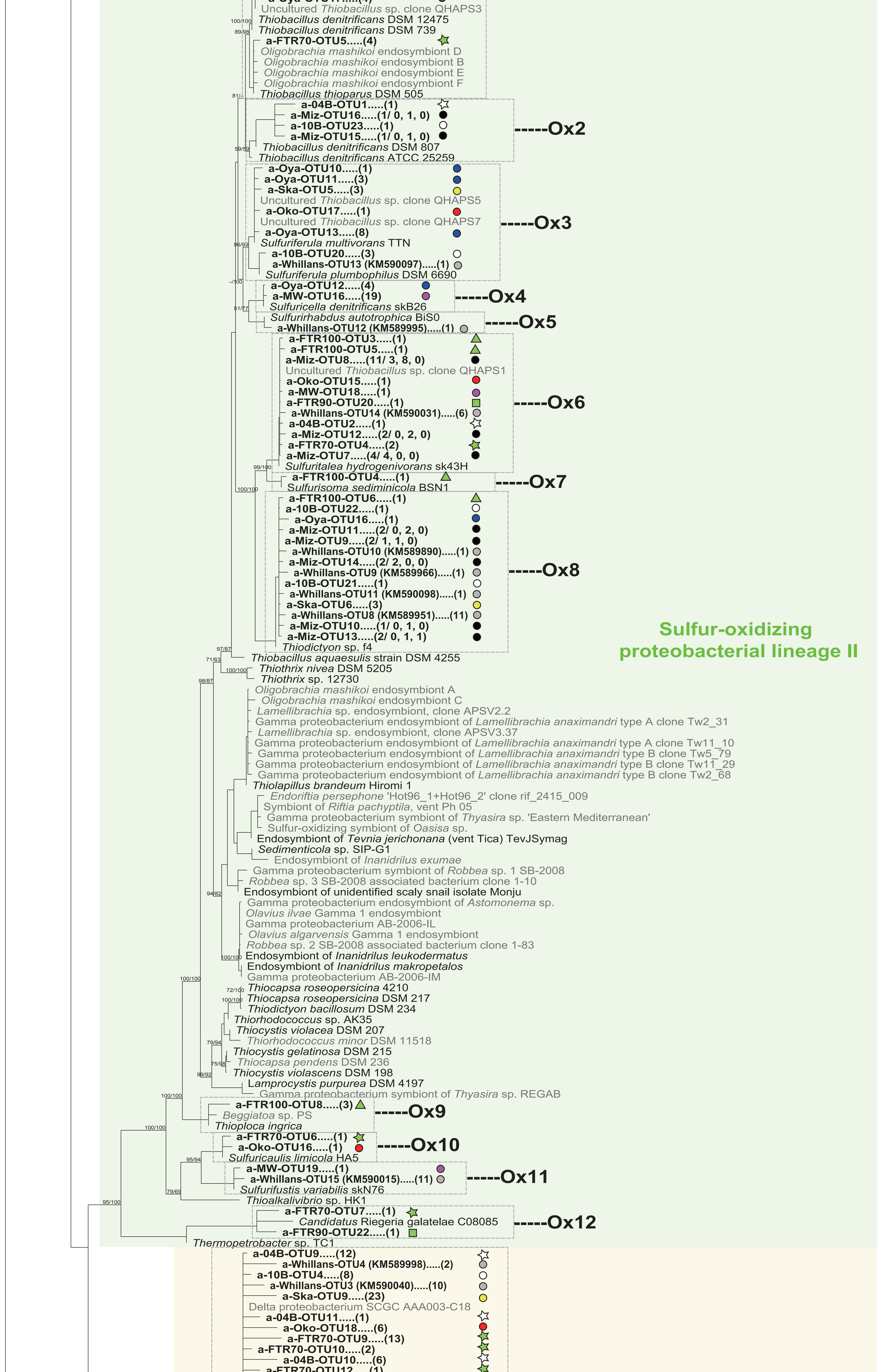

Uncultured Thiobacillus sp. clone QHAPS3
Thiobacillus denitrificans DSM 12475
Thiobacillus denitrificans DSM 739
a-FTR70-OTU5.....(4)
Oligobrachia mashikoi endosymbiont D
Oligobrachia mashikoi endosymbiont B
Oligobrachia mashikoi endosymbiont E
Oligobrachia mashikoi endosymbiont F
Thiobacillus thioparus DSM 505
a-04B-OTU1.....(1)
a-Miz-OTU16.....(1/ 0, 1, 0)
a-10B-OTU23.....(1)
a-Miz-OTU15.....(1/ 0, 1, 0)
Thiobacillus denitrificans DSM 807
Thiobacillus denitrificans ATCC 25259
-----Ox2
a-Oya-OTU10.....(1)
a-Oya-OTU11.....(3)
a-Ska-OTU5.....(3)
Uncultured Thiobacillus sp. clone QHAPS5
a-Oko-OTU17.....(1)
Uncultured Thiobacillus sp. clone QHAPS7
a-Oya-OTU13.....(8)
Sulfuriferula multivorans TTN
a-10B-OTU20.....(3)
a-Whillans-OTU13 (KM590097).....(1)
Sulfuriferula plumbophilus DSM 6690
-----Ox3
a-Oya-OTU12.....(4)
a-MW-OTU16.....(19)
Sulfuricella denitrificans skB26
-----Ox4
Sulfurirhabdus autotrophica BiS0
a-Whillans-OTU12 (KM589995).....(1)
-----Ox5
a-FTR100-OTU3.....(1)
a-FTR100-OTU5.....(1)
a-Miz-OTU8.....(11/ 3, 8, 0)
Uncultured Thiobacillus sp. clone QHAPS1
a-Oko-OTU15.....(1)
a-MW-OTU18.....(1)
a-FTR90-OTU20.....(1)
a-Whillans-OTU14 (KM590031).....(6)
a-04B-OTU2.....(1)
a-Miz-OTU12.....(2/ 0, 2, 0)
a-FTR70-OTU4.....(2)
a-Miz-OTU7.....(4/ 4, 0, 0)
Sulfuritalea hydrogenivorans sk43H
-----Ox6
a-FTR100-OTU4.....(1)
Sulfurisoma sediminicola BSN1
-----Ox7
a-FTR100-OTU6.....(1)
a-10B-OTU22.....(1)
a-Oya-OTU16.....(1)
a-Miz-OTU11.....(2/ 0, 2, 0)
a-Miz-OTU9.....(2/ 1, 1, 0)
a-Whillans-OTU10 (KM589890).....(1)
a-Miz-OTU14.....(2/ 2, 0, 0)
a-Whillans-OTU9 (KM589966).....(1)
a-10B-OTU21.....(1)
a-Whillans-OTU11 (KM590098).....(1)
a-Ska-OTU6.....(3)
a-Whillans-OTU8 (KM589951).....(11)
a-Miz-OTU10.....(1/ 0, 1, 0)
a-Miz-OTU13.....(2/ 0, 1, 1)
Thiodictyon sp. f4
-----Ox8
Sulfur-oxidizing proteobacterial lineage II
Thiobacillus aquaesulis strain DSM 4255
Thiothrix nivea DSM 5205
Thiothrix sp. 12730
Oligobrachia mashikoi endosymbiont A
Oligobrachia mashikoi endosymbiont C
Lamellibrachia sp. endosymbiont, clone APSV2.2
Gamma proteobacterium endosymbiont of Lamellibrachia anaximandri type A clone Tw2_31
Lamellibrachia sp. endosymbiont, clone APSV3.37
Gamma proteobacterium endosymbiont of Lamellibrachia anaximandri type A clone Tw11_10
Gamma proteobacterium endosymbiont of Lamellibrachia anaximandri type B clone Tw5_79
Gamma proteobacterium endosymbiont of Lamellibrachia anaximandri type B clone Tw11_29
Gamma proteobacterium endosymbiont of Lamellibrachia anaximandri type B clone Tw2_68
Thiolapillus brandeum Hiromi 1
Endoriftia persephone 'Hot96_1+Hot96_2' clone rif_2415_009
Symbiont of Riftia pachyptila, vent Ph 05
Gamma proteobacterium symbiont of Thyasira sp. 'Eastern Mediterranean'
Sulfur-oxidizing symbiont of Oasisa sp.
Endosymbiont of Tevnia jerichonana (vent Tica) TevJSymag
Sedimenticola sp. SIP-G1
Endosymbiont of Inanidrilus exumae
Gamma proteobacterium symbiont of Robbea sp. 1 SB-2008
Robbea sp. 3 SB-2008 associated bacterium clone 1-10
Endosymbiont of unidentified scaly snail isolate Monju
Gamma proteobacterium endosymbiont of Astomonema sp.
Olavius ilvae Gamma 1 endosymbiont
Gamma proteobacterium AB-2006-IL
Olavius algarvensis Gamma 1 endosymbiont
Robbea sp. 2 SB-2008 associated bacterium clone 1-83
Endosymbiont of Inanidrilus leukodermatus
Endosymbiont of Inanidrilus makropetalos
Gamma proteobacterium AB-2006-IM
Thiocapsa roseopersicina 4210
Thiocapsa roseopersicina DSM 217
Thiodictyon bacillosum DSM 234
Thiorhodococcus sp. AK35
Thiocystis violacea DSM 207
Thiorhodococcus minor DSM 11518
Thiocystis gelatinosa DSM 215
Thiocapsa pendens DSM 236
Thiocystis violascens DSM 198
Lamprocystis purpurea DSM 4197
Gamma proteobacterium symbiont of Thyasira sp. REGAB
a-FTR100-OTU8.....(3)
Beggiatoa sp. PS
Thioploca ingrica
-----Ox9
a-FTR70-OTU6.....(1)
a-Oko-OTU16.....(1)
Sulfuricaulis limicola HA5
-----Ox10
a-MW-OTU19.....(1)
a-Whillans-OTU15 (KM590015).....(11)
Sulfurifustis variabilis skN76
-----Ox11
Thioalkalivibrio sp. HK1
a-FTR70-OTU7.....(1)
Candidatus Riegeria galatelae C08085
a-FTR90-OTU22.....(1)
-----Ox12
Thermopetrobacter sp. TC1
a-04B-OTU9.....(12)
a-Whillans-OTU4 (KM589998).....(2)
a-10B-OTU4.....(8)
a-Whillans-OTU3 (KM590040).....(10)
a-Ska-OTU9.....(23)
Delta proteobacterium SCGC AAA003-C18
a-04B-OTU11.....(1)
a-Oko-OTU18.....(6)
a-FTR70-OTU9.....(13)
a-FTR70-OTU10.....(2)
a-04B-OTU10.....(6)

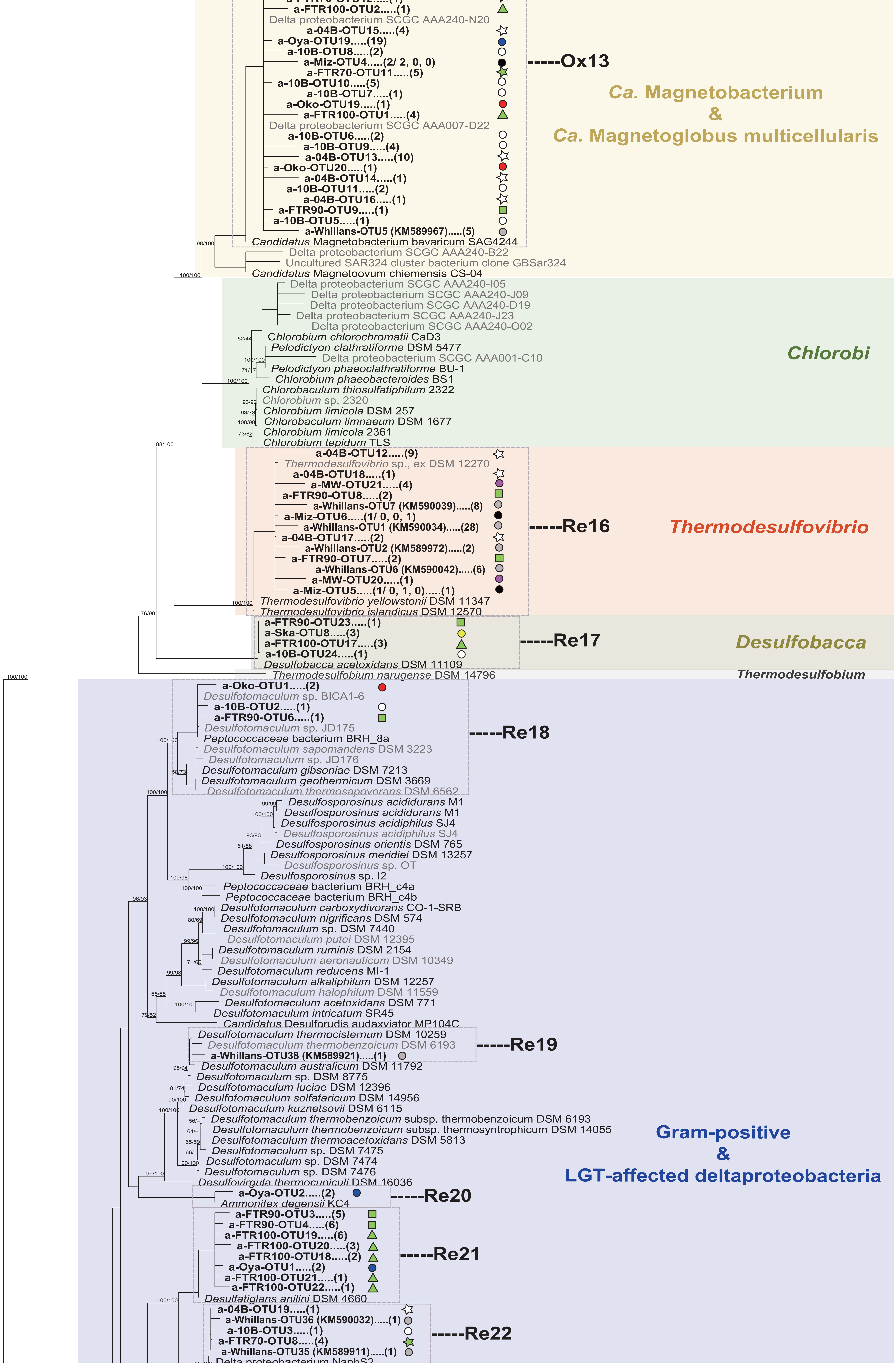

a-FTR100-OTU2.....(1)
Delta proteobacterium SCGC AAA240-N20
a-04B-OTU15.....(4)
a-Oya-OTU19.....(19)
a-10B-OTU8.....(2)
a-Miz-OTU4.....(2/ 2, 0, 0)
a-FTR70-OTU11.....(5)
a-10B-OTU10.....(5)
a-10B-OTU7.....(1)
a-Oko-OTU19.....(1)
a-FTR100-OTU1.....(4)
Delta proteobacterium SCGC AAA007-D22
a-10B-OTU6.....(2)
a-10B-OTU9.....(4)
a-04B-OTU13.....(10)
a-Oko-OTU20.....(1)
a-04B-OTU14.....(1)
a-10B-OTU11.....(2)
a-04B-OTU16.....(1)
a-FTR90-OTU9.....(1)
a-10B-OTU5.....(1)
a-Whillans-OTU5 (KM589967).....(5)
Candidatus Magnetobacterium bavaricum SAG4244
-----Ox13
Ca. Magnetobacterium
&
Ca. Magnetoglobus multicellularis
Delta proteobacterium SCGC AAA240-B22
Uncultured SAR324 cluster bacterium clone GBSar324
Candidatus Magnetoovum chiemensis CS-04
Delta proteobacterium SCGC AAA240-I05
Delta proteobacterium SCGC AAA240-J09
Delta proteobacterium SCGC AAA240-D19
Delta proteobacterium SCGC AAA240-J23
Delta proteobacterium SCGC AAA240-O02
Chlorobium chlorochromatii CaD3
Pelodictyon clathratiforme DSM 5477
Delta proteobacterium SCGC AAA001-C10
Pelodictyon phaeoclathratiforme BU-1
Chlorobium phaeobacteroides BS1
Chlorobaculum thiosulfatiphilum 2322
Chlorobium sp. 2320
Chlorobium limicola DSM 257
Chlorobaculum limnaeum DSM 1677
Chlorobium limicola 2361
Chlorobium tepidum TLS
Chlorobi
a-04B-OTU12.....(9)
Thermodesulfovibrio sp., ex DSM 12270
a-04B-OTU18.....(1)
a-MW-OTU21.....(4)
a-FTR90-OTU8.....(2)
a-Whillans-OTU7 (KM590039).....(8)
a-Miz-OTU6.....(1/ 0, 0, 1)
a-Whillans-OTU1 (KM590034).....(28)
a-04B-OTU17.....(2)
a-Whillans-OTU2 (KM589972).....(2)
a-FTR90-OTU7.....(2)
a-Whillans-OTU6 (KM590042).....(6)
a-MW-OTU20.....(1)
a-Miz-OTU5.....(1/ 0, 1, 0).....(1)
Thermodesulfovibrio yellowstonii DSM 11347
Thermodesulfovibrio islandicus DSM 12570
-----Re16
Thermodesulfovibrio
a-FTR90-OTU23.....(1)
a-Ska-OTU8.....(3)
a-FTR100-OTU17.....(3)
a-10B-OTU24.....(1)
Desulfobacca acetoxidans DSM 11109
-----Re17
Desulfobacca
Thermodesulfobium narugense DSM 14796
Thermodesulfobium
a-Oko-OTU1.....(2)
Desulfotomaculum sp. BICA1-6
a-10B-OTU2.....(1)
a-FTR90-OTU6.....(1)
Desulfotomaculum sp. JD175
Peptococcaceae bacterium BRH_8a
Desulfotomaculum sapomandens DSM 3223
Desulfotomaculum sp. JD176
Desulfotomaculum gibsoniae DSM 7213
Desulfotomaculum geothermicum DSM 3669
Desulfotomaculum thermosapovorans DSM 6562
-----Re18
Desulfosporosinus acididurans M1
Desulfosporosinus acididurans M1
Desulfosporosinus acidiphilus SJ4
Desulfosporosinus acidiphilus SJ4
Desulfosporosinus orientis DSM 765
Desulfosporosinus meridiei DSM 13257
Desulfosporosinus sp. OT
Desulfosporosinus sp. I2
Peptococcaceae bacterium BRH_c4a
Peptococcaceae bacterium BRH_c4b
Desulfotomaculum carboxydivorans CO-1-SRB
Desulfotomaculum nigrificans DSM 574
Desulfotomaculum sp. DSM 7440
Desulfotomaculum putei DSM 12395
Desulfotomaculum ruminis DSM 2154
Desulfotomaculum aeronauticum DSM 10349
Desulfotomaculum reducens MI-1
Desulfotomaculum alkaliphilum DSM 12257
Desulfotomaculum halophilum DSM 11559
Desulfotomaculum acetoxidans DSM 771
Desulfotomaculum intricatum SR45
Candidatus Desulforudis audaxviator MP104C
Desulfotomaculum thermocisternum DSM 10259
Desulfotomaculum thermobenzoicum DSM 6193
a-Whillans-OTU38 (KM589921).....(1)
-----Re19
Desulfotomaculum australicum DSM 11792
Desulfotomaculum sp. DSM 8775
Desulfotomaculum luciae DSM 12396
Desulfotomaculum solfataricum DSM 14956
Desulfotomaculum kuznetsovii DSM 6115
Desulfotomaculum thermobenzoicum subsp. thermobenzoicum DSM 6193
Desulfotomaculum thermobenzoicum subsp. thermosyntrophicum DSM 14055
Desulfotomaculum thermoacetoxidans DSM 5813
Desulfotomaculum sp. DSM 7475
Desulfotomaculum sp. DSM 7474
Desulfotomaculum sp. DSM 7476
Desulfovirgula thermocuniculi DSM 16036
Gram-positive
&
LGT-affected deltaproteobacteria
a-Oya-OTU2.....(2)
Ammonifex degensii KC4
-----Re20
a-FTR90-OTU3.....(5)
a-FTR90-OTU4.....(6)
a-FTR100-OTU19.....(6)
a-FTR100-OTU20.....(3)
a-FTR100-OTU18.....(2)
a-Oya-OTU1.....(2)
a-FTR100-OTU21.....(1)
a-FTR100-OTU22.....(1)
Desulfatiglans anilini DSM 4660
-----Re21
a-04B-OTU19.....(1)
a-Whillans-OTU36 (KM590032).....(1)
a-10B-OTU3.....(1)
a-FTR70-OTU8.....(4)
a-Whillans-OTU35 (KM589911).....(1)
-----Re22

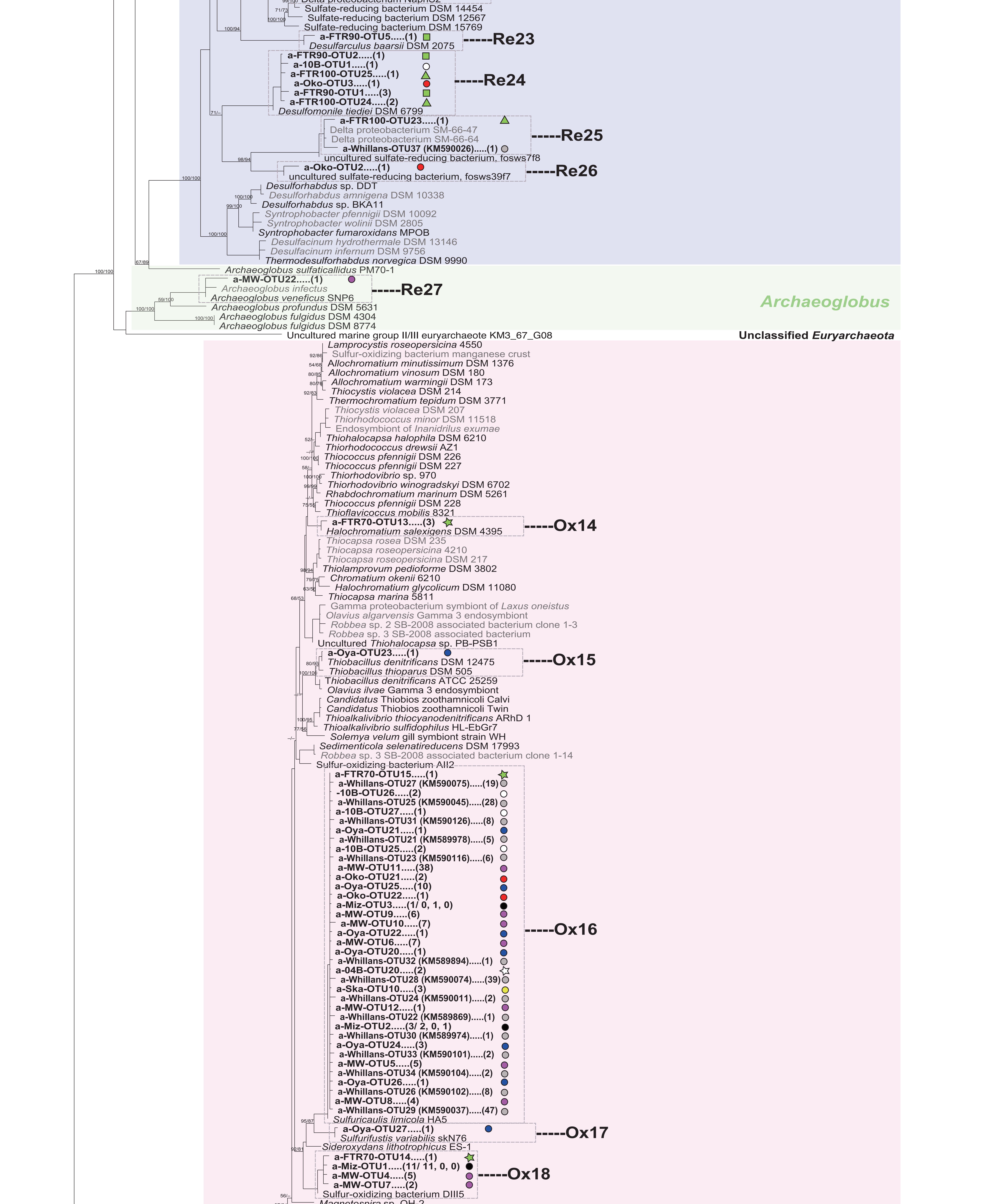

Sulfate-reducing bacterium DSM 14454
Sulfate-reducing bacterium DSM 12567
Sulfate-reducing bacterium DSM 15769
a-FTR90-OTU5.....(1)
Desulfarculus baarsii DSM 2075
-----Re23
a-FTR90-OTU2.....(1)
a-10B-OTU1.....(1)
a-FTR100-OTU25.....(1)
a-Oko-OTU3.....(1)
a-FTR90-OTU1.....(3)
a-FTR100-OTU24.....(2)
Desulfomonile tiedjei DSM 6799
-----Re24
a-FTR100-OTU23.....(1)
Delta proteobacterium SM-66-47
Delta proteobacterium SM-66-64
a-Whillans-OTU37 (KM590026).....(1)
uncultured sulfate-reducing bacterium, fosws7f8
-----Re25
a-Oko-OTU2.....(1)
uncultured sulfate-reducing bacterium, fosws39f7
-----Re26
Desulforhabdus sp. DDT
Desulforhabdus amnigena DSM 10338
Desulforhabdus sp. BKA11
Syntrophobacter pfennigii DSM 10092
Syntrophobacter wolinii DSM 2805
Syntrophobacter fumaroxidans MPOB
Desulfacinum hydrothermale DSM 13146
Desulfacinum infernum DSM 9756
Thermodesulforhabdus norvegica DSM 9990
Archaeoglobus sulfaticallidus PM70-1
a-MW-OTU22.....(1)
Archaeoglobus infectus
Archaeoglobus veneficus SNP6
-----Re27
Archaeoglobus profundus DSM 5631
Archaeoglobus fulgidus DSM 4304
Archaeoglobus fulgidus DSM 8774
Archaeoglobus
Uncultured marine group II/III euryarchaeote KM3_67_G08
Unclassified Euryarchaeota
Lamprocystis roseopersicina 4550
Sulfur-oxidizing bacterium manganese crust
Allochromatium minutissimum DSM 1376
Allochromatium vinosum DSM 180
Allochromatium warmingii DSM 173
Thiocystis violacea DSM 214
Thermochromatium tepidum DSM 3771
Thiocystis violacea DSM 207
Thiorhodococcus minor DSM 11518
Endosymbiont of Inanidrilus exumae
Thiohalocapsa halophila DSM 6210
Thiorhodococcus drewsii AZ1
Thiococcus pfennigii DSM 226
Thiococcus pfennigii DSM 227
Thiorhodovibrio sp. 970
Thiorhodovibrio winogradskyi DSM 6702
Rhabdochromatium marinum DSM 5261
Thiococcus pfennigii DSM 228
Thioflavicoccus mobilis 8321
a-FTR70-OTU13.....(3)
Halochromatium salexigens DSM 4395
-----Ox14
Thiocapsa rosea DSM 235
Thiocapsa roseopersicina 4210
Thiocapsa roseopersicina DSM 217
Thiolamprovum pedioforme DSM 3802
Chromatium okenii 6210
Halochromatium glycolicum DSM 11080
Thiocapsa marina 5811
Gamma proteobacterium symbiont of Laxus oneistus
Olavius algarvensis Gamma 3 endosymbiont
Robbea sp. 2 SB-2008 associated bacterium clone 1-3
Robbea sp. 3 SB-2008 associated bacterium
Uncultured Thiohalocapsa sp. PB-PSB1
a-Oya-OTU23.....(1)
Thiobacillus denitrificans DSM 12475
Thiobacillus thioparus DSM 505
-----Ox15
Thiobacillus denitrificans ATCC 25259
Olavius ilvae Gamma 3 endosymbiont
Candidatus Thiobios zoothamnicoli Calvi
Candidatus Thiobios zoothamnicoli Twin
Thioalkalivibrio thiocyanodenitrificans ARhD 1
Thioalkalivibrio sulfidophilus HL-EbGr7
Solemya velum gill symbiont strain WH
Sedimenticola selenatireducens DSM 17993
Robbea sp. 3 SB-2008 associated bacterium clone 1-14
Sulfur-oxidizing bacterium AII2
a-FTR70-OTU15.....(1)
a-Whillans-OTU27 (KM590075).....(19)
-10B-OTU26.....(2)
a-Whillans-OTU25 (KM590045).....(28)
a-10B-OTU27.....(1)
a-Whillans-OTU31 (KM590126).....(8)
a-Oya-OTU21.....(1)
a-Whillans-OTU21 (KM589978).....(5)
a-10B-OTU25.....(2)
a-Whillans-OTU23 (KM590116).....(6)
a-MW-OTU11.....(38)
a-Oko-OTU21.....(2)
a-Oya-OTU25.....(10)
a-Oko-OTU22.....(1)
a-Miz-OTU3.....(1/ 0, 1, 0)
a-MW-OTU9.....(6)
a-MW-OTU10.....(7)
a-Oya-OTU22.....(1)
a-MW-OTU6.....(7)
a-Oya-OTU20.....(1)
a-Whillans-OTU32 (KM589894).....(1)
a-04B-OTU20.....(2)
a-Whillans-OTU28 (KM590074).....(39)
a-Ska-OTU10.....(3)
a-Whillans-OTU24 (KM590011).....(2)
a-MW-OTU12.....(1)
a-Whillans-OTU22 (KM589869).....(1)
a-Miz-OTU2.....(3/ 2, 0, 1)
a-Whillans-OTU30 (KM589974).....(1)
a-Oya-OTU24.....(3)
a-Whillans-OTU33 (KM590101).....(2)
a-MW-OTU5.....(5)
a-Whillans-OTU34 (KM590104).....(2)
a-Oya-OTU26.....(1)
a-Whillans-OTU26 (KM590102).....(8)
a-MW-OTU8.....(4)
a-Whillans-OTU29 (KM590037).....(47)
Sulfuricaulis limicola HA5
-----Ox16
a-Oya-OTU27.....(1)
Sulfurifustis variabilis skN76
-----Ox17
Sideroxydans lithotrophicus ES-1
a-FTR70-OTU14.....(1)
a-Miz-OTU1.....(11/ 11, 0, 0)
a-MW-OTU4.....(5)
a-MW-OTU7.....(2)
Sulfur-oxidizing bacterium DIII5
-----Ox18
Magnetospira sp. QH-2

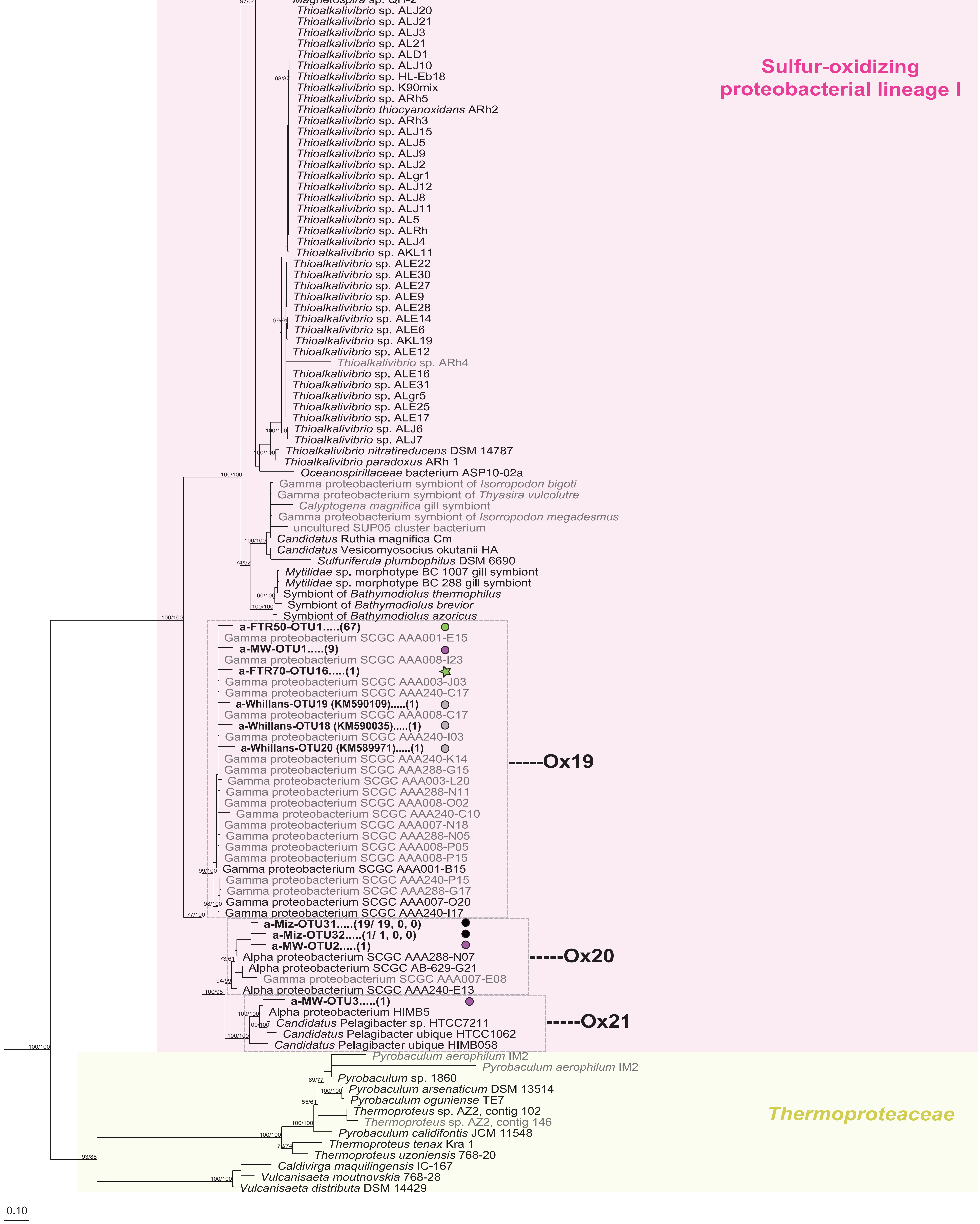
Magnetospira sp. QH-2
Thioalkalivibrio sp. ALJ20
Thioalkalivibrio sp. ALJ21
Thioalkalivibrio sp. ALJ3
Thioalkalivibrio sp. AL21
Thioalkalivibrio sp. ALD1
Thioalkalivibrio sp. ALJ10
Thioalkalivibrio sp. HL-Eb18
Thioalkalivibrio sp. K90mix
Thioalkalivibrio sp. ARh5
Thioalkalivibrio thiocyanoxidans ARh2
Thioalkalivibrio sp. ARh3
Thioalkalivibrio sp. ALJ15
Thioalkalivibrio sp. ALJ5
Thioalkalivibrio sp. ALJ9
Thioalkalivibrio sp. ALJ2
Thioalkalivibrio sp. ALgr1
Thioalkalivibrio sp. ALJ12
Thioalkalivibrio sp. ALJ8
Thioalkalivibrio sp. ALJ11
Thioalkalivibrio sp. AL5
Thioalkalivibrio sp. ALRh
Thioalkalivibrio sp. ALJ4
Thioalkalivibrio sp. AKL11
Thioalkalivibrio sp. ALE22
Thioalkalivibrio sp. ALE30
Thioalkalivibrio sp. ALE27
Thioalkalivibrio sp. ALE9
Thioalkalivibrio sp. ALE28
Thioalkalivibrio sp. ALE14
Thioalkalivibrio sp. ALE6
Thioalkalivibrio sp. AKL19
Thioalkalivibrio sp. ALE12
Thioalkalivibrio sp. ARh4
Thioalkalivibrio sp. ALE16
Thioalkalivibrio sp. ALE31
Thioalkalivibrio sp. ALgr5
Thioalkalivibrio sp. ALE25
Thioalkalivibrio sp. ALE17
Thioalkalivibrio sp. ALJ6
Thioalkalivibrio sp. ALJ7
Thioalkalivibrio nitratireducens DSM 14787
Thioalkalivibrio paradoxus ARh 1
Oceanospirillaceae bacterium ASP10-02a
Gamma proteobacterium symbiont of Isorropodon bigoti
Gamma proteobacterium symbiont of Thyasira vulcolutre
Calyptogena magnifica gill symbiont
Gamma proteobacterium symbiont of Isorropodon megadesmus
uncultured SUP05 cluster bacterium
Candidatus Ruthia magnifica Cm
Candidatus Vesicomyosocius okutanii HA
Sulfuriferula plumbophilus DSM 6690
Mytilidae sp. morphotype BC 1007 gill symbiont
Mytilidae sp. morphotype BC 288 gill symbiont
Symbiont of Bathymodiolus thermophilus
Symbiont of Bathymodiolus brevior
Symbiont of Bathymodiolus azoricus
a-FTR50-OTU1.....(67)
Gamma proteobacterium SCGC AAA001-E15
a-MW-OTU1.....(9)
Gamma proteobacterium SCGC AAA008-I23
a-FTR70-OTU16.....(1)
Gamma proteobacterium SCGC AAA003-J03
Gamma proteobacterium SCGC AAA240-C17
a-Whillans-OTU19 (KM590109).....(1)
Gamma proteobacterium SCGC AAA008-C17
a-Whillans-OTU18 (KM590035).....(1)
Gamma proteobacterium SCGC AAA240-I03
a-Whillans-OTU20 (KM589971).....(1)
Gamma proteobacterium SCGC AAA240-K14
Gamma proteobacterium SCGC AAA288-G15
Gamma proteobacterium SCGC AAA003-L20
Gamma proteobacterium SCGC AAA288-N11
Gamma proteobacterium SCGC AAA008-O02
Gamma proteobacterium SCGC AAA240-C10
Gamma proteobacterium SCGC AAA007-N18
Gamma proteobacterium SCGC AAA288-N05
Gamma proteobacterium SCGC AAA008-P05
Gamma proteobacterium SCGC AAA008-P15
Gamma proteobacterium SCGC AAA001-B15
Gamma proteobacterium SCGC AAA240-P15
Gamma proteobacterium SCGC AAA288-G17
Gamma proteobacterium SCGC AAA007-O20
Gamma proteobacterium SCGC AAA240-I17
a-Miz-OTU31.....(19/ 19, 0, 0)
a-Miz-OTU32.....(1/ 1, 0, 0)
a-MW-OTU2.....(1)
Alpha proteobacterium SCGC AAA288-N07
Alpha proteobacterium SCGC AB-629-G21
Gamma proteobacterium SCGC AAA007-E08
Alpha proteobacterium SCGC AAA240-E13
a-MW-OTU3.....(1)
Alpha proteobacterium HIMB5
Candidatus Pelagibacter sp. HTCC7211
Candidatus Pelagibacter ubique HTCC1062
Candidatus Pelagibacter ubique HIMB058
Pyrobaculum aerophilum IM2
Pyrobaculum aerophilum IM2
Pyrobaculum sp. 1860
Pyrobaculum arsenaticum DSM 13514
Pyrobaculum oguniense TE7
Thermoproteus sp. AZ2, contig 102
Thermoproteus sp. AZ2, contig 146
Pyrobaculum calidifontis JCM 11548
Thermoproteus tenax Kra 1
Thermoproteus uzoniensis 768-20
Caldivirga maquilingensis IC-167
Vulcanisaeta moutnovskia 768-28
Vulcanisaeta distributa DSM 14429
Sulfur-oxidizing proteobacterial lineage I
Thermoproteaceae
-----Ox19
-----Ox20
-----Ox21
0.10

Fig. S3. Consensus phylogeny of AprBA sequences. This figure is an un-collapsed version of the consensus tree shown in Figure 1. The 154 sequences of shorter AprA or AprBA and 225 AprA sequences of OTUs obtained in this study were added to the consensus tree (based on 320 AprBA sequences of core sequence set) by the parsimony interactive tool in ARB, and shown in gray and bold type, respectively. Circles on the tree indicate the clone libraries of respective OTUs with colors indicated in legend. Colored boxes indicate phylogenetic clusters or branches. Dashed-line boxes (I to XLV) indicate the detailed affiliations of OTUs shown in Table 2. Accession numbers of reference sequences are shown in Tables S2 and S3.

	34	35	36	37	38	39	40	41	42	43	44	45	46	47	48	49	50
Gram-positive & LGT-affected deltaproteobacteria																	
Desulfotomaculum sp. DSM 7475	M	V	L	D	K	E	-	-	-	-	-	-	R	M	K	S	Y
Desulfarculus baarsii DSM 2075	M	L	L	D	K	D	G	S	M	G	Y	G	A	M	K	A	F
Archaeoglobus																	
Archaeoglobus veneficus SNP6	M	K	L	N	E	E	-	-	-	-	-	-	L	M	K	A	Y
Desulfobacteraceae																	
Desulfosarcina variabilis Montpellier	M	V	L	D	P	N	-	-	-	-	-	-	A	M	K	A	Y
Thermodesulfobacteriaceae																	
Thermodesulfatator atlanticus DSM 21156	M	I	L	D	T	E	-	-	-	-	-	-	A	M	K	A	Y
Desulfovibrionales																	
Desulfovibrio sp. DSM 9953	M	I	L	D	P	E	-	-	-	-	-	-	E	M	K	A	Y
Desulfobulbaceae																	
Desulfobacterium corrodens DSM 15630	M	V	L	N	V	E	-	-	-	-	-	-	S	M	L	A	Y
Lineage II																	
Chlorobaculum thiosulfatiphilum 2322	M	K	L	D	V	E	-	-	-	-	-	-	R	M	K	A	W
Thermodesulfovibrio																	
Thermodesulfovibrio islandicus DSM 12570	M	T	L	D	R	E	-	-	-	-	-	-	K	M	K	A	F
Desulfobacca																	
Desulfobacca acetoxidans DSM 11109	M	V	L	N	A	E	-	-	-	-	-	-	I	K	K	A	Y
Proteobacterial lineage II																	
Thermopetrobacter sp. TC1	M	K	L	D	V	E	G	E	F	T	G	H	K	M	K	A	F
Endosymbiont of Inanidrilus leukodermatus	.	.	.	.	.	.	.	.	.	.	.	H	A	M	K	S	Y
Endosymbiont of Inanidrilus makropetalos	.	.	.	.	Q	D	G	S	A	T	G	H	A	M	K	S	Y
Endosymbiont of Tevnia jerichonana (vent Tica) TevJSymag	M	K	L	D	K	D	G	S	E	T	G	H	A	M	K	S	F
Endosymbiont of unidentified scaly snail isolate Monju	M	K	L	D	M	D	G	S	E	T	G	H	A	M	K	S	Y
Sedimenticola sp. SIP-G1	M	K	L	D	L	D	G	S	L	T	G	H	A	M	K	S	F
Thiolapillus brandeum Hiromi 1	M	K	L	D	M	D	G	S	M	T	G	H	A	M	K	S	F
Thiocapsa roseopersicina 4210	M	K	L	D	Q	D	G	S	E	T	G	H	A	M	K	A	F
Thiocapsa roseopersicina DSM 217	M	K	L	D	Q	D	G	S	E	T	G	H	A	M	K	A	F
Thiodictyon bacillosum DSM 234	.	.	.	.	.	.	.	.	.	.	.	H	A	M	K	A	F
Thiocystis gelatinosa DSM 215	M	K	L	D	Q	D	G	S	E	T	G	H	A	M	K	A	F
Thiocystis violacea DSM 207	M	K	L	D	Q	D	G	S	E	T	G	H	A	M	K	A	F
Thiocystis violascens DSM 198	M	K	L	D	Q	D	G	S	E	T	G	H	A	M	K	A	F
Thiorhodococcus sp. AK35	M	K	L	D	R	D	G	S	E	T	G	H	A	M	R	A	F
Thioalkalivibrio sp. HK1	M	K	L	D	M	D	G	S	E	T	G	H	A	M	K	A	W
Lamprocystis purpurea DSM 4197	M	K	L	D	Q	D	G	S	E	T	G	H	A	M	K	A	F
Thiobacillus denitrificans DSM 739	.	.	.	.	.	.	.	.	.	.	.	H	A	M	R	A	F
Thiobacillus denitrificans DSM 12475	M	K	L	D	K	D	G	S	E	T	G	H	A	M	R	A	F
Thiobacillus thioparus DSM 505	M	K	L	D	K	D	G	S	E	T	G	H	A	M	R	A	F
Thiodictyon sp. f4	.	.	.	.	K	D	G	S	E	T	G	H	A	M	R	A	Y
Thiobacillus denitrificans DSM 807	.	.	.	.	.	.	.	.	.	.	.	H	A	M	R	A	F
Thiobacillus aquaesulis strain DSM 4255	M	K	L	D	K	D	G	S	E	T	G	H	A	M	K	A	F
Thiobacillus denitrificans ATCC 25259	M	K	L	D	K	D	G	S	E	T	G	H	A	M	R	A	F
Sulfuritalea hydrogenivorans sk43H	M	K	L	D	K	D	G	S	E	T	G	H	A	M	K	A	F
Sulfuriferula plumbophilus DSM 6690	M	M	L	D	K	D	G	S	A	T	G	H	A	M	K	A	Y
Sulfuriferula multivorans TTN	M	K	L	D	K	D	G	S	E	T	G	H	A	M	K	A	F
Sulfuricella sp. BiS0	M	M	L	D	K	D	G	S	A	T	G	H	A	M	K	A	Y
Sulfuricella denitrificans skB26	M	K	L	D	K	D	G	S	E	T	G	H	A	M	K	A	F
Thiothrix sp. 12730	M	K	L	D	V	D	G	S	A	T	G	H	A	M	K	A	Y
Thiothrix nivea DSM 5205	M	K	L	D	V	D	G	S	A	T	G	H	A	M	R	A	Y
Sulfurisoma sediminicola BSN1	M	K	L	D	K	D	G	S	D	T	G	H	A	M	K	A	F
Sulfuricaulis limicola HA5	M	K	L	D	K	D	G	S	E	T	G	H	A	M	K	A	W
Sulfurifustis variabilis skN76	M	K	L	D	K	D	G	S	E	T	G	H	P	M	K	A	W
Thioploca ingrica	M	K	L	D	K	D	G	S	E	T	G	H	A	M	K	A	Y
Thermodesulfobium																	
Thermodesulfobium narugense DSM 14796	M	T	L	N	R	E	-	-	-	-	-	-	I	M	K	A	Y
Euryarchaeota																	
Uncultured marine group II/III euryarchaeote KM3_67_G08	M	K	L	K	G	D	-	-	-	-	-	-	-	-	K	G	F
Proteobacterial lineage I																	
Alpha proteobacterium SCGC AAA240-E13	.	.	.	.	.	T	-	-	-	-	-	-	I	R	R	A	V
Thermoproteaceae																	
Thermoproteus uzoniensis 768-20	M	Q	Y	N	P	K	-	-	-	-	-	-	T	R	R	A	Y
Vulcanisaeta distributa DSM 14429	M	R	F	T	K	S	G	-	-	-	I	F	G	R	K	A	Y

Fig. S4. Multiple alignment of AprB sequences showing a unique insertion conserved in proteobacterial lineage II-AprB sequences and *Desulfarculus baarsii* DSM 2075 (highlighted by boxes). Amino acid positions are numbered according to AprB sequence of *Vulcanisaeta moutnovskia* 768-28. A hyphen indicates a gap, and is shown in gray. A dot indicates no sequence information available for this position.

AprB

Lineage II	118	119	120	121	122	123	124	125	126	127	128	129	130	131	132	133
Chlorobaculum thiosulfatiphilum 2322	R	T	T	S	E	G	-	-	-	S	I	D	L	Y	S	G
Chlorobium limicola DSM 257	R	T	T	S	E	G	-	-	-	S	I	D	L	Y	S	G
Chlorobaculum limnaeum DSM 1677	R	T	T	S	E	G	-	-	-	S	I	D	L	Y	S	G
Chlorobium limicola 2361	R	T	T	S	E	G	-	-	-	S	I	D	L	Y	S	G
Pelodictyon clathratiforme DSM 5477	R	T	T	S	E	G	-	-	-	S	I	D	L	Y	S	G
Pelodictyon phaeoclathratiforme BU-1	R	T	T	S	E	G	-	-	-	S	I	D	L	Y	S	G
Chlorobium tepidum TLS	R	T	T	S	E	G	-	-	-	S	I	D	P	Y	S	G
Chlorobium chlorochromatii CaD3	R	T	T	A	E	G	-	-	-	S	I	E	P	Y	A	G
Chlorobium phaeobacteroides BS1	R	T	T	A	E	G	-	-	-	S	I	E	L	Y	S	G
Candidatus Magnetobacterium bavaricum SAG4244	R	T	T	A	E	G	-	-	-	S	I	D	P	Y	K	G
Candidatus Magnetoovum chiemensis CS-04	R	T	T	A	E	G	-	-	-	S	V	N	P	Y	A	G
Thermodesulfovibrio																
Thermodesulfovibrio islandicus DSM 12570	R	L	T	P	E	G	Y	W	N	A	D	N	V	Y	A	G
Thermodesulfovibrio yellowstonii DSM 11347	R	L	T	P	E	G	Y	W	N	A	D	N	I	Y	A	G

AprA

Lineage II	153	154	155	156	157	158	159	160	161	162	163	164	165	166	167	168
Chlorobaculum thiosulfatiphilum 2322	M	D	G	S	K	P	-	-	-	-	A	P	K	L	A	E
Chlorobium limicola DSM 257	M	D	G	S	K	P	-	-	-	-	A	P	K	L	A	E
Chlorobaculum limnaeum DSM 1677	M	D	G	S	K	P	-	-	-	-	A	P	K	L	A	E
Chlorobium limicola 2361	M	D	G	S	K	P	-	-	-	-	A	P	K	L	A	E
Pelodictyon clathratiforme DSM 5477	Q	D	G	A	K	P	-	-	-	-	A	P	K	L	S	E
Pelodictyon phaeoclathratiforme BU-1	Q	D	G	A	K	P	-	-	-	-	A	P	K	L	S	E
Chlorobium tepidum TLS	M	D	G	A	K	P	-	-	-	-	A	P	K	L	T	E
Chlorobium chlorochromatii CaD3	M	D	G	S	K	P	-	-	-	-	A	P	K	L	T	E
Chlorobium phaeobacteroides BS1	Q	D	G	S	K	P	-	-	-	-	A	P	K	L	T	E
Candidatus Magnetobacterium bavaricum SAG4244	M	D	G	A	Q	P	-	-	-	-	A	K	K	L	S	E
Candidatus Magnetoovum chiemensis CS-04	M	D	G	S	Q	P	-	-	-	-	A	P	K	L	T	E
Thermodesulfovibrio																
Thermodesulfovibrio islandicus DSM 12570	L	D	G	F	Q	A	R	D	A	G	K	P	A	L	K	D
Thermodesulfovibrio yellowstonii DSM 11347	L	D	G	F	Q	A	R	D	A	G	K	P	A	L	K	D

Fig. S5. Multiple alignment of AprB and AprA sequences showing the unique deletions shared by *Ca.* M. bavaricum, *Ca*. M. multicellularis and *Chlorobi* (highlighted by gray boxes). Amino acid positions of AprB and AprA are numbered according to those of *Vulcanisaeta moutnovskia* 768-28 and *Desulfotalea psychrophila* LSv54, respectively.

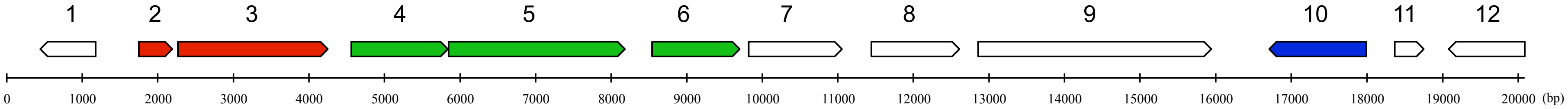

Number	Locus_tag	Annotation	Best BLASTP hit organism	BLAST score	Query cover	E value	Ident	Accession
1	JY97_RS07900	Fe-S oxidoreductase	*Desulfosarcina* sp. BuS5	275	97%	6.0E-89	52%	WP_035264408.1
**2**	**JY97_RS07905**	**adenylylsulfate reductase subunit B**	***Desulfosarcina* sp. BuS5**	**266**	**97%**	**3.0E-88**	**87%**	**WP_027353073.1**
**3**	**JY97_RS07910**	**adenylylsulfate reductase subunit A**	***Desulfatitalea tepidiphila***	**1224**	**100%**	**0**	**86%**	**WP_054029362.1**
**4**	**JY97_RS07915**	**heterodisulfide reductase subunit A**	***Desulfosarcina cetonica***	**719**	**100%**	**0**	**78%**	**WP_054689148.1**
**5**	**JY97_RS07920**	**heterodisulfide reductase subunit A**	***Desulfosarcina* sp. BuS5**	**1273**	**100%**	**0**	**77%**	**WP_027353076.1**
**6**	**JY97_RS07925**	**heterodisulfide reductase**	**delta proteobacterium PSCGC 5342**	**675**	**100%**	**0**	**82%**	**WP_027982915.1**
7	JY97_RS07930	hypothetical protein	alpha proteobacterium HIMB114	282	98%	4.0E-87	37%	WP_009360024.1
8	JY97_RS07935	RND transporter	*Syntrophobacter fumaroxidans*	354	93%	7.0E-115	51%	WP_011700706.1
9	JY97_RS07940	transporter	*Candidatus* Entotheonella sp. TSY2	1413	100%	0	66%	ETW98394.1
**10**	**JY97_RS07945**	**sulfate adenylyltransferase (Sat)**	***Desulfatitalea tepidiphila***	**772**	**99%**	**0**	**86%**	**WP_054029370.1**
11	JY97_RS07950	pilus assembly protein PilZ	*Desulfobacterium autotrophicum*	104	97%	2.0E-25	41%	WP_015903760.1
12	JY97_RS07955	hypothetical protein	gamma proteobacterium IMCC3088	379	99%	5.0E-127	56%	WP_009575154.1

Fig. S6. *aprBA*-harboring scaffold of *Spirochaeta odontotermitis* JC202. Genes are indicated by arrows, and colored arrows indicate genes involved in dissimilatory sulfate reduction. Table shows the result of blastp search of all coding sequences against non-redundant protein sequence sequence set in GenBank. Accession number of this scaffold is NZ_JRAS01000134.

Organism	Forward primer match (5'–3')	MM #	Reverse primer match (3'–5')	MM #
Allochromatium minutissimum DSM 1376	= = = = = = = = = = = = = = = = = = = =	0	= = = = = = = = = = = M = = M = =	2
Allochromatium vinosum DSM 180	= = = = = = = = = = = = = = = = = = = =	0	= = = = = = = = = = = M = = M = =	2
Allochromatium warmingii DSM 173	= = = = = = = = M = = = = = = = = = = =	1	= = = = = = = = = = = M = = M = =	2
Alpha proteobacterium HIMB5	= = = = = = = = = = = = = = = = = = = =	0	= = = = = M = = = = = = = = M = =	2
Alpha proteobacterium SCGC AAA240-E13	= = = = = = = = M = = = = = = = = = = =	1	= = = = = = = = = = = = = = M = =	1
Alpha proteobacterium SCGC AAA288-N07	= = = = = = = = M = = = = = = = = = = =	1	= = = = = = = = = = = = = = M = =	1
Alpha proteobacterium SCGC AB-629-G21	= = = = = = = = M = = = = = = = = = = =	1	= = = = = = = = = = = = = = M = =	1
Ammonifex degensii KC4	= = = = = = M = M = = = = = = = M = = =	3	= = = = = = = = = = = M = = = = =	1
Archaeoglobus fulgidus DSM 4304	= = = = = = = = M = = = = = = = = = = =	1	= = = = = = = = = = = = = = M = =	1
Archaeoglobus fulgidus DSM 8774	= = = = = = = = M = = = = = = = = = = =	1	= = = = = = = = = = = = = = M = =	1
Archaeoglobus profundus DSM 5631	= = = = = M M = = = = = = = = = = = = =	2	= = = = = = = = = = = M = = M = =	2
Archaeoglobus sulfaticallidus PM70-1	= = = = = = M = M = = = = = M = = = = =	3	= = = = = = = = = = = M = = M = =	2
Archaeoglobus veneficus SNP6	= = = = = = M = = = = = = = = = = = = =	1	= = = = = = = = = = = M = = M = =	2
Caldivirga maquilingensis IC-167	= = = = = = = = M = = = = = = = = = = =	1	= = = = = M = = = = = M = = M = =	3
Candidatus Desulforudis audaxviator MP104C	= = = = = = M = M = = = = = = = = = = =	2	= = = = = = = = = = = M = = = = =	1
Candidatus Magnetobacterium bavaricum SAG4244	= = = = = = = = = = = = = = M = = = = =	1	= = = = = = = = = = = = = = = = =	0
Candidatus Magnetoglobus multicellularis Araruama	= = = = = = = = M = = = = = = = = = = =	1	= = = = = = = = M = = = = = M = =	2
Candidatus Magnetoovum chiemensis CS-04	= = = = = M = = M = = = = = M = = = = =	3	= = = = = = = = M = = M = = = = =	2
Candidatus Pelagibacter sp. HTCC7211	= = = = = M = = = = = = = = = = = = = =	1	= = = = = M = = M = = = = = M = =	3
Candidatus Pelagibacter ubique HIMB058	= = = = = M = = = = = = = = M = = = = =	2	= = = = = = = = M = = = = = M = =	2
Candidatus Pelagibacter ubique HTCC1062	= = = = = = = = = = = = = = = = = = = =	0	= = = = = = = = = = = = = = M = =	1
Candidatus Ruthia magnifica Cm	= = = = = = = = M = = = = = = = = = = =	1	= = = = = = = = M = = = = = = = =	1
Candidatus Vesicomyosocius okutanii HA	= = = = = = = = M = = = = = = = = = = =	1	= = = = = = = = M = = = = = M = =	2
Chlorobaculum limnaeum DSM 1677	= = = = = = = = = = = = = = = = = = = =	0	= = = = = = = = = = = M = = = = =	1
Chlorobaculum thiosulfatiphilum 2322	= = = = = = = = = = = = = = = = = = = =	0	= = = = = = = = = = = M = = = = =	1
Chlorobium chlorochromatii CaD3	= = = = = M = = = = = = = = = = = = = =	1	= = = = = = = = M = = M = = M = =	3
Chlorobium limicola 2361	= = = = = = = = = = = = = = = = = = = =	0	= = = = = = = = = = = = = = = = =	0
Chlorobium limicola DSM 257	= = = = = = = = = = = = = = = = = = = =	0	= = = = = = = = = = = M = = = = =	1
Chlorobium phaeobacteroides BS1	= = = = = = = = = = = = = = = = = = = =	0	= = = = = = = = = = = = = = M = =	1
Chlorobium tepidum TLS	= = = = = = = = = = = = = = = = = = = =	0	= = = = = = = = = = = M = = = = =	1
Chromatium okenii 6210	= = = = = = = = = = = = = = = = = = = =	0	= = = = = = = = M = = M = = = = =	2
Delta proteobacterium NaphS2	= = = = = = M = = = = = = = = = = = = =	1	= = = = = = = = M = = M = = M = =	3
Desulfarculus baarsii DSM 2075	= = = = = = = = = = = = = = = = = = = =	0	= = = = = = = = = = = M = = M = =	2
Desulfatibacillum alkenivorans AK-01	= = = = = = = = = = = = = = = = = = = =	0	= = = = = = = = M = = M = = = = =	2
Desulfatibacillum sp. Pnd3	= = = = = = = = = = = = = = = = = = = =	0	= = = = = = = = M = = M = = = = =	2
Desulfatiglans anilini DSM 4660	= = = = = = M = = = = = = = = = = = = =	1	= = = = = = = = = = = M = = = = =	1
Desulfatirhabdium butyrativorans DSM 18734	= = = = = = = = = = = = = = = = = = = =	0	= = = = = = = = = = = M = = = = =	1
Desulfatitalea tepidiphila S28OL1	= = = = = = = = = = = = = = = = = = = =	0	= = = = = = = = = = = M = = M = =	2
Desulfobacca acetoxidans DSM 11109	= = = = = M M M M = = = = = = = = = = =	4	= = = = = = = = = = = = = = = = =	0
Desulfobacter curvatus DSM 3379	= = = = = = = = = = = = = = = = = = = =	0	= = = = = = = = M = = = = = M = =	2
Desulfobacter postgatei 2ac9	= = = = = = = = = = = = = = = = = = = =	0	= = = = = = = = M = = = = = M = =	2
Desulfobacter sp. DSM 2035	= = = = = = = = = = = = = = = = = = = =	0	= = = = = = = = M = = = = = M = =	2
Desulfobacter sp. DSM 2057	= = = = = = = = = = = = = = = = = = = =	0	= = = = = = = = M = = = = = M M =	3
Desulfobacterium autotrophicum DSM 3382	= = = = = = = = = = = = = = = = = = = =	0	= = = = = M = = M = = = = = M = =	3
Desulfobacterium corrodens DSM 15630	= = = = = = = = = = = = = = = = = = = =	0	= = = = = = = = M = = M = = M = =	3
Desulfobacterium indolicum DSM 3383	= = = = = M = = = = = = = = = = = = = =	1	= = = = = = = = = = = = = = = = =	0
Desulfobacterium sp. PM4	= = = = = M = = = = = = = = = = = = = =	1	= = = = = = = = M = = = = = M = =	2
Desulfobacterium zeppelinii DSM 9120	= = = = = = = = = = = = = = = = = = = =	0	= = = = = = = = M = = = = = = = =	1
Desulfobacula phenolica DSM 3384	= = = = = = = = = = = = = = = = = = = =	0	= = = = = = = = M = = = = = M = =	2
Desulfobacula toluolica Tol2	= = = = = = = = = = = = = = = = = = = =	0	= = = = = = = = M = = = = = M = =	2
Desulfobotulus sapovorans DSM 2055	= = = = = = = = = = = = = = = = = = = =	0	= = = = = = = = M = = = = = M = =	2
Desulfobulbus elongatus DSM 2908	= = = = = = = = = = = = = = = = = = = =	0	= = = = = = = = = = = = = = M = =	1
Desulfobulbus marinus DSM 2058	= = = = = = = = = = = = = = = = = = = =	0	= = = = = = = = M = = = = = M = =	2
Desulfobulbus propionicus DSM 2032	= = = = = = = = = = = = = = = = = = = =	0	= = = = = = = = = = = = = = M = =	1
Desulfobulbus sp. DSM 2033	= = = = = = = = = = = = = = = = = = = =	0	= = = = = = = = = = = = = = M = =	1
Desulfocaldus sp. Hobo	= = = = = = = = = = = = = = = = = = = =	0	= = = = = = = = M = = M = = = = =	2
Desulfocapsa sulfexigens DSM 10523	= = = = = = = = = = = = = = = = = = = =	0	= = = = = M = = M = = = = = M = =	3
Desulfocapsa thiozymogenes DSM 7269	= = = = = = = = = = = = = = = = = = = =	0	= = = = = = = = M = = = = = M = =	2
Desulfococcus multivorans DSM 2059	= = = = = = = = = = = = = = = = = = = =	0	= = = = = = = = = = = M = = M = =	2
Desulfococcus oleovorans Hxd3	= = = = = = = = = = = = = = = = = = = =	0	= = = = = = = = M = = M = = M = =	3
Desulfococcus sp. DSM 8541	= = = = = = = = = = = = = = = = = = = =	0	= = = = = = = = = = = M = = M = =	2
Desulfocurvus vexinensis DSM 17965	= = = = = = = = = = = = = = = = = = = =	0	= = = = = = = = = = = M = = = = =	1
Desulfofrigus sp. HRS-La3x	= = = = = = = = = = = = = = = = = = = =	0	= = = = = = = = M = = = = = M = =	2
Desulfofustis glycolicus DSM 9705	= = = = = = = = = = = = = = = = = = = =	0	= = = = = = = = M = = M = = M = =	3
Desulfohalobium retbaense DSM 5692	= = = = = = = = M = = = = = = = = = = =	1	= = = = = = = = = = = M = = M = =	2
Desulfomicrobium baculatum DSM 4028	= = = = = = = = = = = = = = = = = = = =	0	= = = = = = = = = = = = = = = = =	0
Desulfomicrobium escambiense DSM 10707	= = = = = = = = = = = = = = = = = = = =	0	= = = = = = = = = = = M = = = = =	1
Desulfomonile tiedjei DSM 6799	= = = = = = M = = = = = = = = = = = = =	1	= = = = = M = = = = = M = = = = =	2
Desulfonatronovibrio hydrogenovorans DSM 9292	= = = = = = = = = = = = = = = = = = = =	0	= = = = = = = = M = = = = = = = =	1
Desulfonatronovibrio magnus AHT22	= = = = = = = = = = = = = = = = = = = =	0	= = = = = = = = M = = = = = = = =	1
Desulfonatronum lacustre DSM 10312	= = = = = = = = = = = = = = = = = = = =	0	= = = = = = = = = = = M = = = = =	1
Desulfonatronum thiodismutans MLF-1	= = = = = = = = = = = = = = = = = = = =	0	= = = = = = = = = = = M = = = = =	1
Desulfonauticus autotrophicus DSM 4206	= = = = = = = = M = = = = = = = = = = =	1	= = = = = = = = M = = = = = M = =	2
Desulfonauticus sp. A7A	= = = = = = = = = = = = = = = = = = = =	0	= = = = = = = = M = = = = = M = =	2
Desulfonauticus submarinus DSM 15269	= = = = = = = = M = = = = = = = = = = =	1	= = = = = = = = M = = M = = M = =	3
Desulfonema limicola Jadebusen	= = = = = = = = = = = = = = = = = = = =	0	= = = = = = = = M = = = = = M = =	2
Desulfonema magnum Montpellier	= = = = = = = = = = = = = = = = = = = =	0	= = = = = = = = M = = M = = = = =	2
Desulfopila aestuarii MSL86	= = = = = = = = = = = = = = = = = = = =	0	= = = = = = = = M = = = = = M = =	2
Desulfopila inferna JS_SRB250Lac	= = = = = = = = = = = = = = = = = = = =	0	= = = = = = = = M = = = = = = = =	1
Desulfoplanes formicivorans Pf12B	= = = = = = = = = = = = = = = = = = = =	0	= = = = = = = = M = = = = = = = =	1
Desulforegula conservatrix DSM 13527	= = = = = = = = = = = = = = = = = = = =	0	= = = = = = = = M = = = = = M = =	2
Desulforhabdus sp. BKA11	= = = = = = = = = = = = = = = = = = = =	0	= = = = = = = = M = = M = = = = =	2
Desulforhabdus sp. DDT	= = = = = = = = = = = = = = = = = = = =	0	= = = = = = = = M = = = = = = = =	1
Desulfosarcina cetonica DSM 7267	= = = = = = = = = = = = = = = = = = = =	0	= = = = = = = = = = = M = = = = =	1
Desulfosarcina ovata oxyS1	= = = = = = = = = = = = = = = = = = = =	0	= = = = = = = = = = = M = = M = =	2
Desulfosarcina variabilis Montpellier	= = = = = = = = = = = = = = = = = = = =	0	= = = = = = = = = = = = = = M = =	1
Desulfospira joergensenii DSM 10085	= = = = = = = = = = = = = = = = = = = =	0	= = = = = M = = M = = = = = M = =	3
Desulfosporosinus acididurans M1	= = = = = M M = M = = = = = = = M = = =	4	= = = = = M = = = = = M = = M = =	3
Desulfosporosinus acididurans M1	= = = = = M M = M = = = = = = = M = = =	4	= = = = = M = = = = = M = = M = =	3
Desulfosporosinus acidiphilus SJ4	= = = = = M M = M = = = = = = = M = = =	4	= = = = = = = = M = = = = = M = =	2
Desulfosporosinus meridiei DSM 13257	= = = = = M M = M = = = = = = = M = = =	4	= = = = = = = = = = = = = = = = =	0
Desulfosporosinus orientis DSM 765	= = = = = M M = M = = = = = = = M = = =	4	= = = = = = = = M = = M = = = = =	2
Desulfosporosinus sp. I2	= = = = = M M = M = = = = = = = M = = =	4	= = = = = = = = = = = = = = M = =	1
Desulfotalea psychrophila LSv54	= = = = = = = = = = = = = = = = = = = =	0	= = = = = = = = M = = = = = = = =	1
Desulfothermus naphthae DSM 13418	= = = = = = = = = = = = = = = = = = = =	0	= = = = = = = = M = = = = = M = =	2
Desulfotignum balticum DSM 7044	= = = = = = = = = = = = = = = = = = = =	0	= = = = = = = = M = = M = = = = =	2
Desulfotignum phosphitoxidans DSM 13687	= = = = = = = = = = = = = = = = = = = =	0	= = = = = = = = M = = M = = = = =	2
Desulfotignum sp. DSM 7120	= = = = = = = = = = = = = = = = = = = =	0	= = = = = = = = M = = M = = M = =	3
Desulfotomaculum acetoxidans DSM 771	= = = = = = = = = = = = = = = = = = = =	0	= = = = = = = = M = = M = = = = =	2

Desulfotomaculum alkaliphilum DSM 12257	=	=	=	=	=	M	=	=	=	=	=	=	M	=	M	=	=	=	=	=	3	=	=	=	=	=	=	=	=	M	=	=	=	=	=	=	=	=	1
Desulfotomaculum australicum DSM 11792	=	=	=	=	=	=	=	=	=	=	=	=	=	=	=	=	=	=	=	=	0	=	=	=	=	=	=	=	=	M	=	=	M	=	=	=	=	=	2
Desulfotomaculum carboxydivorans CO-1-SRB	=	=	=	=	=	=	=	=	=	=	=	=	M	=	M	=	=	=	=	=	2	=	=	=	=	=	=	=	=	M	=	=	M	=	=	M	=	=	3
Desulfotomaculum geothermicum DSM 3669	=	=	=	=	=	=	=	=	=	=	=	=	=	=	=	=	=	=	=	=	0	=	=	=	=	=	=	=	=	=	=	=	M	=	=	=	=	=	1
Desulfotomaculum gibsoniae DSM 7213	=	=	=	=	=	=	=	=	=	=	=	=	=	=	=	=	=	=	=	=	0	=	=	=	=	=	=	=	=	=	=	=	=	=	=	M	=	=	1
Desulfotomaculum intricatum SR45	=	=	=	=	=	M	=	=	=	=	=	=	=	=	=	=	=	=	=	=	1	=	=	=	=	=	=	=	=	=	=	=	=	=	=	M	=	=	1
Desulfotomaculum kuznetsovii DSM 6115	=	=	=	=	=	=	=	=	=	=	=	=	=	=	=	=	=	=	=	=	0	=	=	=	=	=	=	=	=	=	=	=	M	=	=	M	=	=	2
Desulfotomaculum luciae DSM 12396	=	=	=	=	=	=	=	=	=	=	=	=	=	=	=	=	=	=	=	=	0	=	=	=	=	=	=	=	=	M	=	=	M	=	=	M	=	=	3
Desulfotomaculum nigrificans DSM 574	=	=	=	=	=	=	=	=	=	=	=	=	M	=	M	=	=	=	=	=	2	=	=	=	=	=	=	=	=	M	=	=	M	=	=	M	=	=	3
Desulfotomaculum reducens MI-1	=	=	=	=	=	M	=	=	=	=	=	=	M	=	M	=	=	=	=	=	3	=	=	=	=	=	=	=	=	M	=	=	M	=	=	=	=	=	2
Desulfotomaculum ruminis DSM 2154	=	=	=	=	=	=	=	=	=	=	=	=	M	=	M	=	=	=	=	=	2	=	=	=	=	=	=	=	=	M	=	=	M	=	=	=	=	=	2
Desulfotomaculum solfataricum DSM 14956	=	=	=	=	=	=	=	=	=	=	=	=	=	=	=	=	=	=	=	=	0	=	=	=	=	=	=	=	=	=	=	=	M	=	=	M	=	=	2
Desulfotomaculum sp. DSM 7440	=	=	=	=	=	=	=	=	=	=	=	=	M	=	M	=	=	=	=	=	2	=	=	=	=	=	=	=	=	M	=	=	M	=	=	M	=	=	3
Desulfotomaculum sp. DSM 7474	=	=	=	=	=	M	=	=	=	=	=	=	=	=	=	=	=	=	=	=	1	=	=	=	=	=	=	=	=	=	=	=	M	=	=	M	=	=	2
Desulfotomaculum sp. DSM 7475	=	=	=	=	=	M	=	=	=	=	=	=	=	=	=	=	=	=	=	=	1	=	=	=	=	=	=	=	=	=	=	=	M	=	=	M	=	=	2
Desulfotomaculum sp. DSM 7476	=	=	=	=	=	M	=	=	=	=	=	=	=	=	=	=	=	=	=	=	1	=	=	=	=	=	=	=	=	=	=	=	M	=	=	M	=	=	2
Desulfotomaculum sp. DSM 8775	=	=	=	=	=	=	=	=	=	=	=	=	=	=	=	=	=	=	=	=	0	=	=	=	=	=	=	=	=	M	=	=	M	=	=	=	=	=	2
Desulfotomaculum thermoacetoxidans DSM 5813	=	=	=	=	=	M	=	=	=	=	=	=	=	=	=	=	=	=	=	=	1	=	=	=	=	=	=	=	=	=	=	=	M	=	=	M	=	=	2
Desulfotomaculum thermobenzoicum subsp. thermobenzoicum DSM 619	=	=	=	=	=	M	=	=	=	=	=	=	=	=	=	=	=	=	=	=	1	=	=	=	=	=	=	=	=	=	=	=	M	=	=	M	=	=	2
Desulfotomaculum thermobenzoicum subsp. thermosyntrophicum DSM 1	=	=	=	=	=	M	=	=	=	=	=	=	=	=	=	=	=	=	=	=	1	=	=	=	=	=	=	=	=	=	=	=	M	=	=	M	=	=	2
Desulfotomaculum thermocisternum DSM 10259	=	=	=	=	=	=	=	=	=	=	=	=	=	=	=	=	=	=	=	=	0	=	=	=	=	=	=	=	=	M	=	=	M	=	=	=	=	=	2
Desulfovibrio acrylicus DSM 10141	=	=	=	=	=	=	=	=	M	=	=	=	=	=	=	=	=	=	=	=	1	=	=	=	=	=	=	=	=	=	=	=	=	=	=	=	=	=	0
Desulfovibrio aespoeensis Aspo-2	=	=	=	=	=	=	=	=	=	=	=	=	=	=	=	=	=	=	=	=	0	=	=	=	=	=	=	=	=	=	=	=	M	=	=	=	=	=	1
Desulfovibrio africanus PCS	=	=	=	=	=	=	=	=	=	=	=	=	=	=	=	=	=	=	=	=	0	=	=	=	=	=	M	=	=	=	=	=	M	=	=	=	=	=	2
Desulfovibrio africanus subsp. africanus DSM 2603	=	=	=	=	=	=	=	=	=	=	=	=	=	=	=	=	=	=	=	=	0	=	=	=	=	=	M	=	=	=	=	=	=	=	=	=	=	=	1
Desulfovibrio africanus Walvis Bay	=	=	=	=	=	=	=	=	=	=	=	=	=	=	=	=	=	=	=	=	0	=	=	=	=	=	M	=	=	=	=	=	=	=	=	=	=	=	1
Desulfovibrio alaskensis DSM 16109	=	=	=	=	=	=	=	=	M	=	=	=	=	=	=	=	=	=	=	=	1	=	=	=	=	=	=	=	=	=	=	=	=	=	=	=	=	=	0
Desulfovibrio alaskensis G20	=	=	=	=	=	=	=	=	M	=	=	=	=	=	=	=	=	=	=	=	1	=	=	=	=	=	=	=	=	=	=	=	=	=	=	=	=	=	0
Desulfovibrio alcoholivorans DSM 5433	=	=	=	=	=	=	=	=	=	=	=	=	=	=	=	=	=	=	=	=	0	=	=	=	=	=	=	=	=	=	=	=	M	=	=	=	=	=	1
Desulfovibrio alkalitolerans DSM 16529	=	=	=	=	=	=	=	=	=	=	=	=	=	=	=	=	=	=	=	=	0	=	=	=	=	=	=	=	=	=	=	=	M	=	=	=	=	=	1
Desulfovibrio aminophilus DSM 12254	=	=	=	=	=	=	=	=	M	=	=	=	=	=	=	=	=	=	=	=	1	=	=	=	=	=	=	=	=	=	=	=	M	=	=	=	=	=	1
Desulfovibrio bastinii DSM 16055	=	=	=	=	=	=	=	=	M	=	=	=	=	=	=	=	=	=	=	=	1	=	=	=	=	=	M	=	=	M	=	=	=	=	=	M	=	=	3
Desulfovibrio cuneatus DSM 11391	=	=	=	=	=	=	=	=	=	=	=	=	=	=	=	=	=	=	=	=	0	=	=	=	=	=	=	=	=	M	=	=	M	=	=	=	=	=	2
Desulfovibrio desulfuricans ND132	=	=	=	=	=	=	=	=	=	=	=	=	=	=	=	=	=	=	=	=	0	=	=	=	=	=	=	=	=	=	=	=	M	=	=	=	=	=	1
Desulfovibrio desulfuricans subsp. aestuarii DSM 17919	=	=	=	=	=	=	=	=	M	=	=	=	=	=	=	=	=	=	=	=	1	=	=	=	=	=	=	=	=	=	=	=	=	=	=	=	=	=	0
Desulfovibrio desulfuricans subsp. desulfuricans ATCC 27774	=	=	=	=	=	=	=	=	=	=	=	=	=	=	=	=	=	=	=	=	0	=	=	=	=	=	=	=	=	M	=	=	M	=	=	=	=	=	2
Desulfovibrio desulfuricans subsp. desulfuricans DSM 642	=	=	=	=	=	=	=	=	=	=	=	=	=	=	=	=	=	=	=	=	0	=	=	=	=	=	=	=	=	M	=	=	M	=	=	M	=	=	3
Desulfovibrio ferrophilus DSM 15579	=	=	=	=	=	=	=	=	=	=	=	=	=	=	=	=	=	=	=	=	0	=	=	=	=	=	=	=	=	=	=	=	=	=	=	M	=	=	1
Desulfovibrio frigidus DSM 17176	=	=	=	=	=	=	=	=	M	=	=	=	=	=	=	=	=	=	=	=	1	=	=	=	=	=	M	=	=	=	=	=	=	=	=	=	=	=	1
Desulfovibrio fructosivorans JJ	=	=	=	=	=	=	=	=	=	=	=	=	=	=	=	=	=	=	=	=	0	=	=	=	=	=	=	=	=	=	=	=	M	=	=	=	=	=	1
Desulfovibrio gigas ATCC 19364	=	=	=	=	=	=	=	=	=	=	=	=	=	=	=	=	=	=	=	=	0	=	=	=	=	=	=	=	=	=	=	=	M	=	=	=	=	=	1
Desulfovibrio gigas DSM 1382	=	=	=	=	=	=	=	=	=	=	=	=	=	=	=	=	=	=	=	=	0	=	=	=	=	=	=	=	=	=	=	=	M	=	=	=	=	=	1
Desulfovibrio hydrothermalis AM13	=	=	=	=	=	=	=	=	M	=	=	=	=	=	=	=	=	=	=	=	1	=	=	=	=	=	=	=	=	M	=	=	=	=	=	=	=	=	1
Desulfovibrio inopinatus DSM 10711	=	=	=	=	=	=	=	=	=	=	=	=	=	=	=	=	=	=	=	=	0	=	=	=	=	=	=	=	=	=	=	=	M	=	=	=	=	=	1
Desulfovibrio longus DSM 6739	=	=	=	=	=	=	=	=	M	=	=	=	=	=	=	=	=	=	=	=	1	=	=	=	=	=	=	=	=	=	=	=	M	=	=	=	=	=	1
Desulfovibrio magneticus IFRC170	=	=	=	=	=	=	=	=	=	=	=	=	=	=	=	=	=	=	=	=	0	=	=	=	=	=	=	=	=	=	=	=	M	=	=	=	=	=	1
Desulfovibrio magneticus Maddingley MBC34	=	=	=	=	=	=	=	=	=	=	=	=	=	=	=	=	=	=	=	=	0	=	=	=	=	=	=	=	=	=	=	=	M	=	=	=	=	=	1
Desulfovibrio magneticus RS-1	=	=	=	=	=	=	=	=	=	=	=	=	=	=	=	=	=	=	=	=	0	=	=	=	=	=	=	=	=	=	=	=	M	=	=	=	=	=	1
Desulfovibrio oxyclinae DSM 11498	=	=	=	=	=	=	=	=	M	=	=	=	=	=	=	=	=	=	=	=	1	=	=	=	=	=	M	=	=	=	=	=	M	=	=	=	=	=	2
Desulfovibrio piezophilus C1TLV30	=	=	=	=	=	=	=	=	=	=	=	=	=	=	=	=	=	=	=	=	0	=	=	=	=	=	=	=	=	=	=	=	M	=	=	M	=	=	2
Desulfovibrio piger ATCC 29098	=	=	=	=	=	=	=	=	=	=	=	=	=	=	=	=	=	=	=	=	0	=	=	=	=	=	=	=	=	M	=	=	M	=	=	=	=	=	2
Desulfovibrio putealis DSM 16056	=	=	=	=	=	=	=	=	=	=	=	=	=	=	=	=	=	=	=	=	0	=	=	=	=	=	M	=	=	=	=	=	M	=	=	=	=	=	2
Desulfovibrio salexigens DSM 2638	=	=	=	=	=	=	=	=	M	=	=	=	=	=	=	=	=	=	=	=	1	=	=	=	=	=	=	=	=	=	=	=	=	=	=	M	=	=	1
Desulfovibrio sp. A2	=	=	=	=	=	=	=	=	=	=	=	=	=	=	=	=	=	=	=	=	0	=	=	=	=	=	=	=	=	=	=	=	M	=	=	=	=	=	1
Desulfovibrio sp. DSM 9953	=	=	=	=	=	=	=	=	M	=	=	=	=	=	=	=	=	=	=	=	1	=	=	=	=	=	=	=	=	=	=	=	=	=	=	=	=	=	0
Desulfovibrio sp. Dsv1	=	=	=	=	=	=	=	=	=	=	=	=	=	=	=	=	=	=	=	=	0	=	=	=	=	=	=	=	=	=	=	=	M	=	=	M	=	=	2
Desulfovibrio sp. FW1012B	=	=	=	=	=	=	=	=	=	=	=	=	=	=	=	=	=	=	=	=	0	=	=	=	=	=	=	=	=	=	=	=	M	=	=	=	=	=	1
Desulfovibrio sp. JD160	=	=	=	=	=	=	=	=	=	=	=	=	=	=	=	=	=	=	=	=	0	=	=	=	=	=	=	=	=	=	=	=	M	=	=	=	=	=	1
Desulfovibrio sp. L21-Syr-AB	=	=	=	=	=	=	=	=	M	=	=	=	=	=	=	=	=	=	=	=	1	=	=	=	=	=	=	=	=	=	=	=	M	=	=	=	=	=	1
Desulfovibrio sp. TomC	=	=	=	=	=	=	=	=	=	=	=	=	=	=	=	=	=	=	=	=	0	=	=	=	=	=	=	=	=	=	=	=	M	=	=	=	=	=	1
Desulfovibrio sp. U5L	=	=	=	=	=	=	=	=	=	=	=	=	=	=	=	=	=	=	=	=	0	=	=	=	=	=	=	=	=	=	=	=	M	=	=	=	=	=	1
Desulfovibrio sp. X	=	=	=	=	=	=	=	=	M	=	=	=	=	=	=	=	=	=	=	=	1	=	=	=	=	=	=	=	=	=	=	=	=	=	=	M	=	=	1
Desulfovibrio sp. X2	=	=	=	=	=	=	=	=	=	=	=	=	=	=	=	=	=	=	=	=	0	=	=	=	=	=	=	=	=	=	=	=	M	=	=	=	=	=	1
Desulfovibrio sulfodismutans DSM 3969	=	=	=	=	=	=	=	=	=	=	=	=	=	=	=	=	=	=	=	=	0	=	=	=	=	=	=	=	=	=	=	=	M	=	=	=	=	=	1
Desulfovibrio termitidis HI1	=	=	=	=	=	=	=	=	=	=	=	=	=	=	=	=	=	=	=	=	0	=	=	=	=	=	=	=	=	=	=	=	M	=	=	=	=	=	1
Desulfovibrio vulgaris 'Miyazaki F'	=	=	=	=	=	=	=	=	=	=	=	=	=	=	=	=	=	=	=	=	0	=	=	=	=	=	=	=	=	=	=	=	M	=	=	=	=	=	1
Desulfovibrio vulgaris DP4	=	=	=	=	=	=	=	=	=	=	=	=	=	=	=	=	=	=	=	=	0	=	=	=	=	=	=	=	=	=	=	=	M	=	=	=	=	=	1
Desulfovibrio vulgaris Hildenborough	=	=	=	=	=	=	=	=	=	=	=	=	=	=	=	=	=	=	=	=	0	=	=	=	=	=	=	=	=	=	=	=	=	=	=	=	=	=	0
Desulfovibrio vulgaris RCH1	=	=	=	=	=	=	=	=	=	=	=	=	=	=	=	=	=	=	=	=	0	=	=	=	=	=	=	=	=	=	=	=	=	=	=	=	=	=	0
Desulfovibrio vulgaris subsp. vulgaris Hildenborough	=	=	=	=	=	=	=	=	=	=	=	=	=	=	=	=	=	=	=	=	0	=	=	=	=	=	=	=	=	=	=	=	=	=	=	=	=	=	0
Desulfovibrio zosterae DSM 11974	=	=	=	=	=	=	=	=	M	=	=	=	=	=	=	=	=	=	=	=	1	=	=	=	=	=	=	=	=	M	=	=	=	=	=	=	=	=	1
Desulfovirgula thermocuniculi DSM 16036	=	=	=	=	=	=	=	=	=	=	=	=	=	=	=	=	M	=	=	=	1	=	=	=	=	=	=	=	=	=	=	=	=	=	=	=	=	=	0
Desulfurivibrio alkaliphilus AHT2	=	=	=	=	=	=	=	=	=	=	=	=	=	=	=	=	=	=	=	=	0	=	=	=	=	=	=	=	=	=	=	=	M	=	=	=	=	=	1
Endosymbiont of Inanidrilus leukodermatus	=	=	=	=	=	=	=	=	=	=	=	=	=	=	=	=	=	=	=	=	0	=	=	=	=	=	=	=	=	=	=	=	M	=	=	=	=	=	1
Endosymbiont of Inanidrilus makropetalos	=	=	=	=	=	=	=	=	=	=	=	=	=	=	=	=	=	=	=	=	0	=	=	=	=	=	=	=	=	=	=	=	M	=	=	=	=	=	1
Endosymbiont of Tevnia jerichonana (vent Tica) TevJSymag	=	=	=	=	=	=	=	=	=	=	=	=	=	=	=	=	=	=	=	=	0	=	=	=	=	=	=	=	=	M	=	=	=	=	=	=	=	=	1
Endosymbiont of unidentified scaly snail isolate Monju	=	=	=	=	=	=	=	=	=	=	=	=	=	=	=	=	=	=	=	=	0	=	=	=	=	=	=	=	=	=	=	=	M	=	=	M	=	=	2
Gamma proteobacterium SCGC AAA001-B15	=	=	=	=	=	=	=	=	M	=	=	=	=	=	=	=	=	=	=	=	1	=	=	=	=	=	M	=	=	=	=	=	=	=	=	M	=	=	2
Gamma proteobacterium SCGC AAA007-O20	=	=	=	=	=	M	=	=	M	=	=	=	=	=	=	=	=	=	=	=	2	=	=	=	=	=	M	=	=	=	=	=	=	=	=	M	=	=	2
Gamma proteobacterium SCGC AAA240-I17	=	=	=	=	=	M	=	=	M	=	=	=	=	=	=	=	=	=	=	=	2	=	=	=	=	=	M	=	=	=	=	=	=	=	=	M	=	=	2
Halochromatium glycolicum DSM 11080	=	=	=	=	=	=	=	=	=	=	=	=	=	=	=	=	=	=	=	=	0	=	=	=	=	=	=	=	=	=	=	=	M	=	=	M	=	=	2
Halochromatium salexigens DSM 4395	=	=	=	=	=	=	=	=	=	=	=	=	=	=	=	=	=	=	=	=	0	=	=	=	=	=	=	=	=	M	=	=	M	=	=	M	=	=	3
Lamprocystis purpurea DSM 4197	=	=	=	=	=	=	=	=	=	=	=	=	=	=	=	=	=	=	=	=	0	=	=	=	=	=	=	=	=	=	=	=	M	=	=	=	=	=	1
Lamprocystis roseopersicina 4550	=	=	=	=	=	=	=	=	=	=	=	=	=	=	=	=	=	=	=	=	0	=	=	=	=	=	=	=	=	=	=	=	M	=	=	M	=	=	2
Magnetospira sp. QH-2	=	=	=	=	=	=	=	=	=	=	=	=	=	=	=	=	=	=	=	=	0	=	=	=	=	=	=	=	=	=	=	=	=	=	=	=	=	=	0
Oceanospirillaceae bacterium ASP10-02a	=	=	=	=	=	=	=	=	M	=	=	=	=	=	=	=	=	=	=	=	1	=	M	=	=	=	=	=	=	M	=	=	M	=	=	M	=	=	4
Pelodictyon clathratiforme DSM 5477	=	=	=	=	=	=	=	=	M	=	=	=	=	=	=	=	=	=	=	=	1	=	=	=	=	=	=	=	=	=	=	=	=	=	=	=	=	=	0
Pelodictyon phaeoclathratiforme BU-1	=	=	=	=	=	=	=	=	M	=	=	=	=	=	=	=	=	=	=	=	1	=	=	=	=	=	M	=	=	=	=	=	=	=	=	M	=	=	2
Peptococcaceae bacterium BRH_c4a	=	=	=	=	=	=	M	=	=	=	=	=	=	=	=	=	=	=	=	=	1	=	=	=	=	=	=	=	=	=	=	=	M	=	=	M	=	=	2
Peptococcaceae bacterium BRH_c4b	=	=	=	=	=	=	M	=	M	=	=	=	=	=	=	=	=	=	=	=	2	=	=	=	=	=	=	=	=	=	=	=	=	=	=	M	=	=	1
Peptococcaceae bacterium BRH_c8a	=	=	=	=	=	M	=	=	=	=	=	=	M	=	M	=	=	=	=	=	3	=	=	=	=	=	=	=	=	=	=	=	=	=	=	M	=	=	1
Pyrobaculum arsenaticum DSM 13514	=	=	=	=	=	M	M	M	=	M	M	M	=	=	M	=	=	=	=	=	7	=	=	=	=	=	M	=	=	=	=	=	M	=	=	=	M	M	4
Pyrobaculum calidifontis JCM 11548	=	=	=	=	=	=	M	M	=	M	M	M	=	=	=	=	=	=	=	=	5	=	=	=	=	=	=	=	=	M	=	=	M	=	=	=	M	M	4
Pyrobaculum oguniense TE7	=	=	=	=	=	M	M	M	=	M	M	M	=	=	M	=	=	=	=	=	7	=	=	=	=	=	M	=	=	=	=	=	M	=	=	=	M	M	4
Pyrobaculum sp. 1860	=	=	=	=	=	=	M	M	=	M	M	M	=	=	M	=	=	=	=	=	6	=	=	=	=	=	M	=	=	=	=	=	M	=	=	=	M	M	4
Rhabdochromatium marinum DSM 5261	=	=	=	=	=	=	=	=	=	=	=	=	=	=	=	=	=	=	=	=	0	=	=	=	=	=	=	=	=	=	=	=	=	=	=	M	=	=	1
Sedimenticola selenatireducens DSM 17993	=	=	=	=	=	=	=	=	=	=	=	=	=	=	=	=	=	=	=	=	0	=	=	=	=	=	=	=	=	=	=	=	M	=	=	M	=	=	2
Sedimenticola sp. SIP-G1	=	=	=	=	=	=	=	=	=	=	=	=	=	=	=	=	=	=	=	=	0	=	=	=	=	=	=	=	=	=	=	=	M	=	=	M	=	=	2
Sideroxydans lithotrophicus ES-1	=	=	=	=	=	=	=	=	=	=	=	=	=	=	=	=	=	=	=	=	0	=	=	=	=	=	=	=	=	M	=	=	=	=	=	=	=	=	1
Solemya velum gill symbiont strain WH	=	=	=	=	=	=	=	=	=	=	=	=	=	=	=	=	=	=	=	=	0	=	=	=	=	=	=	=	=	M	=	=	=	=	=	M	=	=	2

Spirochaeta odontotermitis JC202	=	=	=	=	=	=	=	=	=	=	=	=	=	=	=	=	=	=	=	=	0	=	=	=	=	=	=	=	=	=	=	=	M	=	=	M	=	=	2
Sulfate-reducing bacterium DSM 12567	=	=	=	=	=	=	M	=	=	=	=	=	=	=	=	=	=	=	=	=	1	=	=	=	=	=	=	=	=	=	=	=	M	=	=	=	=	=	1
Sulfate-reducing bacterium DSM 14454	=	=	=	=	=	=	M	=	=	=	=	=	=	=	=	=	=	=	=	=	1	=	=	=	=	=	=	=	=	M	=	=	M	=	=	M	=	=	3
Sulfate-reducing bacterium DSM 15769	=	=	=	=	=	=	M	=	=	=	=	=	=	=	=	=	=	=	=	=	1	=	=	=	=	=	=	=	=	M	=	=	M	=	=	M	=	=	3
Sulfate-reducing bacterium JHA1	=	=	=	=	=	=	=	=	=	=	=	=	=	=	=	=	=	=	=	=	0	=	=	=	=	=	=	=	=	M	=	=	=	=	=	=	=	=	1
Sulfate-reducing bacterium QLNR1	=	=	=	=	=	=	=	=	=	=	=	=	=	=	=	=	=	=	=	=	0	=	=	=	=	=	=	=	=	M	=	=	M	=	=	M	=	=	3
Sulfur-oxidizing bacterium AII2	=	=	=	=	=	=	=	=	=	=	=	=	=	=	=	=	=	=	=	=	0	=	=	=	=	=	=	=	=	M	M	=	M	=	=	M	=	=	4
Sulfur-oxidizing bacterium DIII5	=	=	=	=	=	=	=	=	=	=	=	=	=	=	=	=	=	=	=	=	0	=	=	=	=	=	=	=	=	M	=	=	M	=	=	M	=	=	3
Sulfuricaulis limicola HA5	=	=	=	=	=	=	=	=	=	=	=	=	=	=	=	=	=	=	=	=	0	=	=	=	=	=	=	=	=	=	=	=	M	=	=	=	=	=	1
Sulfuricaulis limicola HA5	=	=	=	=	=	=	=	=	=	=	=	=	=	=	=	=	=	=	=	=	0	=	=	=	=	=	=	=	=	=	=	=	M	=	=	M	=	=	2
Sulfuricella denitrificans skB26	=	=	=	=	=	=	=	=	=	=	=	=	=	=	=	=	=	=	=	=	0	=	=	=	=	=	=	=	=	=	=	=	=	=	=	=	=	=	0
Sulfuriferula multivorans TTN	=	=	=	=	=	=	=	=	=	=	=	=	=	=	=	=	=	=	=	=	0	=	=	=	=	=	=	=	=	=	=	=	M	=	=	=	=	=	1
Sulfuriferula plumbophilus DSM 6690	=	=	=	=	=	=	=	=	=	=	=	=	=	=	=	=	=	=	=	=	0	=	=	=	=	=	=	=	=	=	=	=	M	=	=	=	=	=	1
Sulfurifustis variabilis skN76	=	=	=	=	=	=	=	=	=	=	=	=	=	=	=	=	=	=	=	=	0	=	=	=	=	=	=	=	=	=	=	=	M	=	=	=	=	=	1
Sulfurifustis variabilis skN76	=	=	=	=	=	=	=	=	=	=	=	=	=	=	=	=	=	=	=	=	0	=	=	=	=	=	=	=	=	=	=	=	M	=	=	=	=	=	1
Sulfurirhabdus autotrophica BiS0	=	=	=	=	=	M	=	=	=	=	=	=	=	=	=	=	=	=	=	=	1	=	=	=	=	=	=	=	=	=	=	=	=	=	=	M	=	=	1
Sulfurisoma sediminicola BSN1	=	=	=	=	=	=	=	=	=	=	=	=	=	=	=	=	=	=	=	=	0	=	=	=	=	=	=	=	=	=	=	=	M	=	=	M	=	=	2
Sulfuritalea hydrogenivorans sk43H	=	=	=	=	=	=	=	=	=	=	=	=	=	=	=	=	=	=	=	=	0	=	=	=	=	=	=	=	=	=	=	=	M	=	=	M	=	=	2
Symbiont of Bathymodiolus azoricus	=	=	=	=	=	=	=	=	M	=	=	=	=	=	=	=	=	=	=	=	1	=	=	=	=	=	=	=	=	=	=	=	M	=	=	M	=	=	2
Symbiont of Bathymodiolus brevior	=	=	=	=	=	=	=	=	M	=	=	=	=	=	=	=	=	=	=	=	1	=	=	=	=	=	=	=	=	=	=	=	=	=	=	M	=	=	1
Symbiont of Bathymodiolus thermophilus	=	=	=	=	=	M	=	=	M	=	=	=	=	=	=	=	=	=	=	=	2	=	=	=	=	=	=	=	=	=	=	=	=	=	=	=	=	=	0
Syntrophobacter fumaroxidans MPOB	=	=	=	=	=	=	=	=	=	=	=	=	=	=	=	=	=	=	=	=	0	=	=	=	=	=	=	=	=	=	=	=	M	=	=	=	=	=	1
Thermochromatium tepidum DSM 3771	=	=	=	=	=	=	=	=	=	=	=	=	=	=	=	=	=	=	=	=	0	=	=	=	=	=	=	=	=	=	=	=	M	=	=	M	=	=	2
Thermodesulfatator atlanticus DSM 21156	=	=	=	=	=	=	=	=	=	=	=	=	=	=	=	=	=	=	=	=	0	=	=	=	=	=	=	=	=	=	=	=	=	=	=	M	=	=	1
Thermodesulfatator indicus DSM 15286	=	=	=	=	=	=	=	=	=	=	=	=	=	=	=	=	=	=	=	=	0	=	=	=	=	=	=	=	=	=	=	=	=	=	=	M	=	=	1
Thermodesulfobacteraceae bacterium strain TF1	=	=	=	=	=	=	=	=	=	=	=	=	=	=	=	=	=	=	=	=	0	=	=	=	=	=	M	=	=	=	=	=	=	=	=	M	=	=	2
Thermodesulfobacterium commune DSM 2178	=	=	=	=	=	=	=	=	=	=	=	=	=	=	=	=	=	=	=	=	0	=	=	=	=	=	=	=	=	M	=	=	=	=	=	M	=	=	2
Thermodesulfobacterium geofontis OPF15	=	=	=	=	=	=	=	=	M	=	=	=	=	=	=	=	=	=	=	=	1	=	=	=	=	=	=	=	=	=	=	=	=	=	=	M	=	=	1
Thermodesulfobacterium hveragerdense DSM 12571	=	=	=	=	=	=	=	=	=	=	=	=	=	=	=	=	=	=	=	=	0	=	=	=	=	=	=	=	=	M	=	=	=	=	=	M	=	=	2
Thermodesulfobacterium thermophilum DSM 1276	=	=	=	=	=	=	=	=	=	=	=	=	=	=	=	=	=	=	=	=	0	=	=	=	=	=	=	=	=	M	=	=	M	=	=	M	=	=	3
Thermodesulfobium narugense DSM 14796	=	=	=	=	=	=	M	=	M	M	M	M	=	=	M	M	M	=	=	=	8	=	=	=	=	=	M	=	=	M	=	=	M	=	=	M	M	=	5
Thermodesulforhabdus norvegica DSM 9990	=	=	=	=	=	=	=	=	=	=	=	=	=	=	=	=	=	=	=	=	0	=	=	=	=	=	=	=	=	M	=	=	M	=	=	M	=	=	3
Thermodesulfovibrio islandicus DSM 12570	=	=	=	=	=	=	=	=	M	=	=	=	=	=	=	=	=	=	=	=	1	=	=	=	=	=	=	=	=	M	=	=	=	=	=	M	=	=	2
Thermodesulfovibrio yellowstonii DSM 11347	=	=	=	=	=	=	=	=	M	=	=	=	=	=	=	=	=	=	=	=	1	=	=	=	=	=	=	=	=	M	=	=	=	=	=	M	=	=	2
Thermopetrobacter sp. TC1	=	=	=	=	=	=	=	=	=	=	=	=	=	=	=	=	=	=	=	=	0	=	=	=	=	=	=	=	=	=	=	=	M	=	=	M	=	=	2
Thermoproteus sp. AZ2, contig 102	=	=	=	=	=	M	=	=	M	M	M	=	M	=	=	=	=	=	=	=	5	=	=	=	=	=	=	=	=	M	=	=	M	=	=	=	M	M	4
Thermoproteus tenax Kra 1	=	=	=	=	=	M	M	M	M	M	M	=	=	=	M	=	=	=	=	=	7	=	=	=	=	=	=	=	=	M	=	=	M	=	=	M	M	M	5
Thermoproteus uzoniensis 768-20	=	=	=	=	=	M	M	M	=	M	M	=	M	=	=	=	=	=	=	=	6	=	=	=	=	=	=	=	=	M	=	=	M	=	=	M	M	M	5
Thioalkalivibrio nitratireducens DSM 14787	=	=	=	=	=	=	=	=	=	=	=	=	=	=	=	=	=	=	=	=	0	=	=	=	=	=	=	=	=	=	=	=	M	=	=	=	=	=	1
Thioalkalivibrio paradoxus ARh 1	=	=	=	=	=	=	=	=	=	=	=	=	=	=	=	=	=	=	=	=	0	=	=	=	=	=	=	=	=	=	=	=	M	=	=	=	=	=	1
Thioalkalivibrio sp. AKL11	=	=	=	=	=	=	=	=	=	=	=	=	=	=	=	=	=	=	=	=	0	=	=	=	=	=	=	=	=	=	=	=	M	=	=	M	=	=	2
Thioalkalivibrio sp. AKL19	=	=	=	=	=	=	=	=	=	=	=	=	=	=	=	=	=	=	=	=	0	=	=	=	=	=	=	=	=	=	=	=	M	=	=	M	=	=	2
Thioalkalivibrio sp. AL21	=	=	=	=	=	=	=	=	=	=	=	=	=	=	=	=	=	=	=	=	0	=	=	=	=	=	=	=	=	=	=	=	M	=	=	M	=	=	2
Thioalkalivibrio sp. AL5	=	=	=	=	=	=	=	=	=	=	=	=	=	=	=	=	=	=	=	=	0	=	=	=	=	=	=	=	=	=	=	=	M	=	=	M	=	=	2
Thioalkalivibrio sp. ALD1	=	=	=	=	=	=	=	=	=	=	=	=	=	=	=	=	=	=	=	=	0	=	=	=	=	=	=	=	=	=	=	=	M	=	=	M	=	=	2
Thioalkalivibrio sp. ALE12	=	=	=	=	=	=	=	=	=	=	=	=	=	=	=	=	=	=	=	=	0	=	=	=	=	=	=	=	=	=	=	=	M	=	=	M	=	=	2
Thioalkalivibrio sp. ALE14	=	=	=	=	=	=	=	=	=	=	=	=	=	=	=	=	=	=	=	=	0	=	=	=	=	=	=	=	=	=	=	=	M	=	=	M	=	=	2
Thioalkalivibrio sp. ALE16	=	=	=	=	=	=	=	=	=	=	=	=	=	=	=	=	=	=	=	=	0	=	=	=	=	=	=	=	=	=	=	=	M	=	=	M	=	=	2
Thioalkalivibrio sp. ALE17	=	=	=	=	=	=	=	=	=	=	=	=	=	=	=	=	=	=	=	=	0	=	=	=	=	=	=	=	=	=	=	=	M	=	=	M	=	=	2
Thioalkalivibrio sp. ALE22	=	=	=	=	=	=	=	=	=	=	=	=	=	=	=	=	=	=	=	=	0	=	=	=	=	=	=	=	=	=	=	=	M	=	=	M	=	=	2
Thioalkalivibrio sp. ALE25	=	=	=	=	=	=	=	=	=	=	=	=	=	=	=	=	=	=	=	=	0	=	=	=	=	=	=	=	=	=	=	=	M	=	=	M	=	=	2
Thioalkalivibrio sp. ALE27	=	=	=	=	=	=	=	=	=	=	=	=	=	=	=	=	=	=	=	=	0	=	=	=	=	=	=	=	=	=	=	=	M	=	=	M	=	=	2
Thioalkalivibrio sp. ALE28	=	=	=	=	=	=	=	=	=	=	=	=	=	=	=	=	=	=	=	=	0	=	=	=	=	=	=	=	=	=	=	=	M	=	=	M	=	=	2
Thioalkalivibrio sp. ALE30	=	=	=	=	=	=	=	=	=	=	=	=	=	=	=	=	=	=	=	=	0	=	=	=	=	=	=	=	=	=	=	=	M	=	=	M	=	=	2
Thioalkalivibrio sp. ALE31	=	=	=	=	=	=	=	=	=	=	=	=	=	=	=	=	=	=	=	=	0	=	=	=	=	=	=	=	=	=	=	=	M	=	=	M	=	=	2
Thioalkalivibrio sp. ALE6	=	=	=	=	=	=	=	=	=	=	=	=	=	=	=	=	=	=	=	=	0	=	=	=	=	=	=	=	=	=	=	=	M	=	=	M	=	=	2
Thioalkalivibrio sp. ALE9	=	=	=	=	=	=	=	=	=	=	=	=	=	=	=	=	=	=	=	=	0	=	=	=	=	=	=	=	=	=	=	=	M	=	=	M	=	=	2
Thioalkalivibrio sp. ALgr1	=	=	=	=	=	=	=	=	=	=	=	=	=	=	=	=	=	=	=	=	0	=	=	=	=	=	=	=	=	=	=	=	M	=	=	M	=	=	2
Thioalkalivibrio sp. ALgr5	=	=	=	=	=	=	=	=	=	=	=	=	=	=	=	=	=	=	=	=	0	=	=	=	=	=	=	=	=	=	=	=	M	=	=	M	=	=	2
Thioalkalivibrio sp. ALJ10	=	=	=	=	=	=	=	=	=	=	=	=	=	=	=	=	=	=	=	=	0	=	=	=	=	=	=	=	=	=	=	=	M	=	=	M	=	=	2
Thioalkalivibrio sp. ALJ11	=	=	=	=	=	=	=	=	=	=	=	=	=	=	=	=	=	=	=	=	0	=	=	=	=	=	=	=	=	=	=	=	M	=	=	M	=	=	2
Thioalkalivibrio sp. ALJ12	=	=	=	=	=	=	=	=	=	=	=	=	=	=	=	=	=	=	=	=	0	=	=	=	=	=	=	=	=	=	=	=	M	=	=	M	=	=	2
Thioalkalivibrio sp. ALJ15	=	=	=	=	=	=	=	=	=	=	=	=	=	=	=	=	=	=	=	=	0	=	=	=	=	=	=	=	=	=	=	=	M	=	=	M	=	=	2
Thioalkalivibrio sp. ALJ2	=	=	=	=	=	=	=	=	=	=	=	=	=	=	=	=	=	=	=	=	0	=	=	=	=	=	=	=	=	=	=	=	M	=	=	M	=	=	2
Thioalkalivibrio sp. ALJ20	=	=	=	=	=	=	=	=	=	=	=	=	=	=	=	=	=	=	=	=	0	=	=	=	=	=	=	=	=	=	=	=	M	=	=	M	=	=	2
Thioalkalivibrio sp. ALJ21	=	=	=	=	=	=	=	=	=	=	=	=	=	=	=	=	=	=	=	=	0	=	=	=	=	=	=	=	=	=	=	=	M	=	=	M	=	=	2
Thioalkalivibrio sp. ALJ3	=	=	=	=	=	=	=	=	=	=	=	=	=	=	=	=	=	=	=	=	0	=	=	=	=	=	=	=	=	=	=	=	M	=	=	M	=	=	2
Thioalkalivibrio sp. ALJ4	=	=	=	=	=	=	=	=	=	=	=	=	=	=	=	=	=	=	=	=	0	=	=	=	=	=	=	=	=	=	=	=	M	=	=	M	=	=	2
Thioalkalivibrio sp. ALJ5	=	=	=	=	=	=	=	=	=	=	=	=	=	=	=	=	=	=	=	=	0	=	=	=	=	=	=	=	=	=	=	=	M	=	=	M	=	=	2
Thioalkalivibrio sp. ALJ6	=	=	=	=	=	=	=	=	=	=	=	=	=	=	=	=	=	=	=	=	0	=	=	=	=	=	=	=	=	=	=	=	M	=	=	M	=	=	2
Thioalkalivibrio sp. ALJ7	=	=	=	=	=	=	=	=	=	=	=	=	=	=	=	=	=	=	=	=	0	=	=	=	=	=	=	=	=	=	=	=	M	=	=	M	=	=	2
Thioalkalivibrio sp. ALJ8	=	=	=	=	=	=	=	=	=	=	=	=	=	=	=	=	=	=	=	=	0	=	=	=	=	=	=	=	=	=	=	=	M	=	=	M	=	=	2
Thioalkalivibrio sp. ALJ9	=	=	=	=	=	=	=	=	=	=	=	=	=	=	=	=	=	=	=	=	0	=	=	=	=	=	=	=	=	=	=	=	M	=	=	M	=	=	2
Thioalkalivibrio sp. ALRh	=	=	=	=	=	=	=	=	=	=	=	=	=	=	=	=	=	=	=	=	0	=	=	=	=	=	=	=	=	=	=	=	M	=	=	M	=	=	2
Thioalkalivibrio sp. ARh3	=	=	=	=	=	=	=	=	=	=	=	=	=	=	=	=	=	=	=	=	0	=	=	=	=	=	=	=	=	M	=	=	M	=	=	=	=	=	2
Thioalkalivibrio sp. ARh5	=	=	=	=	=	=	=	=	=	=	=	=	=	=	=	=	=	=	=	=	0	=	=	=	=	=	=	=	=	M	=	=	M	=	=	=	=	=	2
Thioalkalivibrio sp. HK1	=	=	=	=	=	M	=	=	=	=	=	=	=	=	=	=	=	=	=	=	1	=	=	=	=	=	M	=	=	=	=	=	M	=	=	M	=	=	3
Thioalkalivibrio sp. HL-Eb18	=	=	=	=	=	=	=	=	=	=	=	=	=	=	=	=	=	=	=	=	0	=	=	=	=	=	=	=	=	=	=	=	M	=	=	M	=	=	2
Thioalkalivibrio sp. K90mix	=	=	=	=	=	=	=	=	=	=	=	=	=	=	=	=	=	=	=	=	0	=	=	=	=	=	=	=	=	=	=	=	M	=	=	M	=	=	2
Thioalkalivibrio sulfidophilus HL-EbGr7	=	=	=	=	=	=	=	=	=	=	=	=	=	=	=	=	=	=	=	=	0	=	=	=	=	=	=	=	=	=	=	=	M	=	=	M	=	=	2
Thioalkalivibrio thiocyanodenitrificans ARhD 1	=	=	=	=	=	=	=	=	=	=	=	=	=	=	=	=	=	=	=	=	0	=	=	=	=	=	=	=	=	=	=	=	M	=	=	=	=	=	1
Thioalkalivibrio thiocyanoxidans ARh2	=	=	=	=	=	=	=	=	=	=	=	=	=	=	=	=	=	=	=	=	0	=	=	=	=	=	=	=	=	M	=	=	M	=	=	=	=	=	2
Thiobacillus aquaesulis strain DSM 4255	=	=	=	=	=	=	=	=	=	=	=	=	=	=	=	=	=	=	=	=	0	=	=	=	=	=	=	=	=	=	=	=	M	=	=	=	=	=	1
Thiobacillus denitrificans ATCC 25259	=	=	=	=	=	=	=	=	=	=	=	=	=	=	=	=	=	=	=	=	0	=	=	=	=	=	=	=	=	=	=	=	M	=	=	=	=	=	1
Thiobacillus denitrificans ATCC 25259	=	=	=	=	=	=	=	=	=	=	=	=	=	=	=	=	=	=	=	=	0	=	=	=	=	=	=	=	=	=	=	=	M	=	=	=	=	=	1
Thiobacillus denitrificans DSM 12475	=	=	=	=	=	=	=	=	=	=	=	=	=	=	=	=	=	=	=	=	0	=	=	=	=	=	=	=	=	=	=	=	M	=	=	=	=	=	1
Thiobacillus denitrificans DSM 12475	=	=	=	=	=	=	=	=	=	=	=	=	=	=	=	=	=	=	=	=	0	=	=	=	=	=	=	=	=	=	=	=	M	=	=	M	=	=	2
Thiobacillus denitrificans DSM 739	=	=	=	=	=	=	=	=	=	=	=	=	=	=	=	=	=	=	=	=	0	=	=	=	=	=	=	=	=	=	=	=	M	=	=	=	=	=	1
Thiobacillus denitrificans DSM 807	=	=	=	=	=	=	=	=	=	=	=	=	=	=	=	=	=	=	=	=	0	=	=	=	=	=	=	=	=	=	=	=	M	=	=	=	=	=	1
Thiobacillus thioparus DSM 505	=	=	=	=	=	=	=	=	=	=	=	=	=	=	=	=	=	=	=	=	0	=	=	=	=	=	=	=	=	=	=	=	M	=	=	=	=	=	1
Thiobacillus thioparus DSM 505	=	=	=	=	=	=	=	=	=	=	=	=	=	=	=	=	=	=	=	=	0	=	=	=	=	=	=	=	=	=	=	=	M	=	=	M	=	=	2
Thiocapsa marina 5811	=	=	=	=	=	=	=	=	=	=	=	=	=	=	=	=	=	=	=	=	0	=	=	=	=	=	=	=	=	=	=	=	M	=	=	M	=	=	2
Thiocapsa roseopersicina 4210	=	=	=	=	=	M	=	=	=	=	=	=	=	=	=	=	=	=	=	=	1	=	=	=	=	=	=	=	=	=	=	=	M	=	=	=	=	=	1
Thiocapsa roseopersicina DSM 217	=	=	=	=	=	=	=	=	=	=	=	=	=	=	=	=	=	=	=	=	0	=	=	=	=	=	=	=	=	=	=	=	M	=	=	=	=	=	1
Thiococcus pfennigii DSM 226	=	=	=	=	=	=	=	=	=	=	=	=	=	=	=	=	=	=	=	=	0	=	=	=	=	=	=	=	=	=	=	=	=	=	=	=	=	=	0
Thiococcus pfennigii DSM 227	=	=	=	=	=	=	=	=	=	=	=	=	=	=	=	=	=	=	=	=	0	=	=	=	=	=	=	=	=	=	=	=	=	=	=	=	=	=	0
Thiococcus pfennigii DSM 228	=	=	=	=	=	=	=	=	=	=	=	=	=	=	=	=	=	=	=	=	0	=	=	=	=	=	=	=	=	=	=	=	=	=	=	=	=	=	0
Thiocystis gelatinosa DSM 215	=	=	=	=	=	M	=	=	=	=	=	=	=	=	=	=	=	=	=	=	1	=	=	=	=	=	=	=	=	=	=	=	M	=	=	=	=	=	1
Thiocystis violacea DSM 207	=	=	=	=	=	=	=	=	=	=	=	=	=	=	=	=	=	=	=	=	0	=	=	=	=	=	=	=	=	=	=	=	=	=	=	=	=	=	0
Thiocystis violacea DSM 214	=	=	=	=	=	M	=	=	=	=	=	=	=	=	=	=	=	=	=	=	1	=	=	=	=	=	=	=	=	=	=	=	M	=	=	=	=	=	1

Thiocystis violascens DSM 198	=	=	=	=	=	=	=	=	=	=	=	=	=	=	=	=	=	=	=	=	0	=	=	=	=	=	=	=	=	=	=	=	M	=	=	=	=	=	1
Thiodictyon bacillosum DSM 234	=	=	=	=	=	=	=	=	=	=	=	=	=	=	=	=	=	=	=	=	0	=	=	=	=	=	=	=	=	=	=	=	M	=	=	=	=	=	1
Thiodictyon sp. f4	=	=	=	=	=	=	=	=	=	=	=	=	=	=	=	=	=	=	=	=	0	=	=	=	=	=	=	=	=	=	=	=	M	=	=	=	=	=	1
Thioflavicoccus mobilis 8321	=	=	=	=	=	=	=	=	=	=	=	=	=	=	=	=	=	=	=	=	0	=	=	=	=	=	=	=	=	=	=	=	=	=	=	=	=	=	0
Thiohalocapsa halophila DSM 6210	=	=	=	=	=	=	=	=	=	=	=	=	=	=	=	=	=	=	=	=	0	=	=	=	=	=	=	=	=	=	=	=	M	=	=	=	=	=	1
Thiolamprovum pedioforme DSM 3802	=	=	=	=	=	=	=	=	=	=	=	=	=	=	=	=	=	=	=	=	0	=	=	=	=	=	=	=	=	=	=	=	M	=	=	M	=	=	2
Thiolapillus brandeum Hiromi 1	=	=	=	=	=	=	=	=	=	=	=	=	=	=	=	=	=	=	=	=	0	=	=	=	=	=	=	=	=	M	=	=	=	=	=	=	=	=	1
Thioploca ingrica	=	=	=	=	=	M	=	=	M	=	=	=	=	=	=	=	=	=	=	=	2	=	=	=	=	=	=	=	=	=	=	=	=	=	=	M	=	=	1
Thiorhodococcus drewsii AZ1	=	=	=	=	=	=	=	=	=	=	=	=	=	=	=	=	=	=	=	=	0	=	=	=	=	=	=	=	=	=	=	=	M	=	=	=	=	=	1
Thiorhodococcus sp. AK35	=	=	=	=	=	=	=	=	=	=	=	=	=	=	=	=	=	=	=	=	0	=	=	=	=	=	=	=	=	=	=	=	M	=	=	=	=	=	1
Thiorhodovibrio sp. 970	=	=	=	=	=	=	=	=	=	=	=	=	=	=	=	=	=	=	=	=	0	=	=	=	=	=	=	=	=	=	=	=	M	=	=	M	=	=	2
Thiorhodovibrio winogradskyi DSM 6702	=	=	=	=	=	=	=	=	=	=	=	=	=	=	=	=	=	=	=	=	0	=	=	=	=	=	=	=	=	=	=	=	M	=	=	M	=	=	2
Thiothrix nivea DSM 5205	=	=	=	=	=	=	=	=	=	=	=	=	=	=	=	=	=	=	=	=	0	=	=	=	=	=	=	=	=	M	=	=	M	=	=	=	=	=	2
Thiothrix sp. 12730	=	=	=	=	=	=	=	=	=	=	=	=	=	=	=	=	=	=	=	=	0	=	=	=	=	=	=	=	=	=	=	=	=	=	=	=	=	=	0
Uncultured Desulfofustis sp. PB-SRB1	=	=	=	=	=	=	=	=	=	=	=	=	=	=	=	=	=	=	=	=	0	=	=	=	=	=	=	=	=	=	=	=	M	=	=	=	=	=	1
Uncultured marine group II/III euryarchaeote KM3_67_G08	=	=	=	=	=	=	=	=	M	M	M	=	=	=	=	=	=	=	=	=	3	=	=	=	=	=	M	=	=	=	=	=	M	=	=	=	M	M	4
uncultured sulfate-reducing bacterium, fosws39f7	=	=	=	=	=	=	M	=	=	=	=	=	=	=	=	=	=	=	=	=	1	=	=	=	=	=	M	=	=	=	=	=	M	=	=	=	=	=	2
uncultured sulfate-reducing bacterium, fosws7f8	=	=	=	=	=	=	M	=	M	=	=	=	=	=	=	=	=	=	=	=	2	=	=	=	=	=	=	=	=	=	=	=	M	=	=	=	=	=	1
Uncultured Thiohalocapsa sp. PB-PSB1	=	=	=	=	=	=	=	=	=	=	=	=	=	=	=	=	=	=	=	=	0	=	=	=	=	=	=	=	=	=	=	=	M	=	=	M	=	=	2
Vulcanisaeta distributa DSM 14429	=	=	=	=	=	=	=	=	M	=	=	=	=	=	=	=	=	=	=	=	1	=	=	=	=	=	M	=	=	M	=	=	=	=	=	M	=	=	3
Vulcanisaeta moutnovskia 768-28	=	=	=	=	=	M	=	=	M	=	=	=	=	=	=	=	=	=	=	=	2	=	=	=	=	=	M	=	=	=	=	=	M	=	=	M	=	=	3

Fig. S7. Species coverage of a primer set AprA-1-FW/AprA-5-RV searched for the core sequence set. M, miss match; =, perfect match; MM#, number of mismatch. Intensity of red color indicates the degree of mismatch.

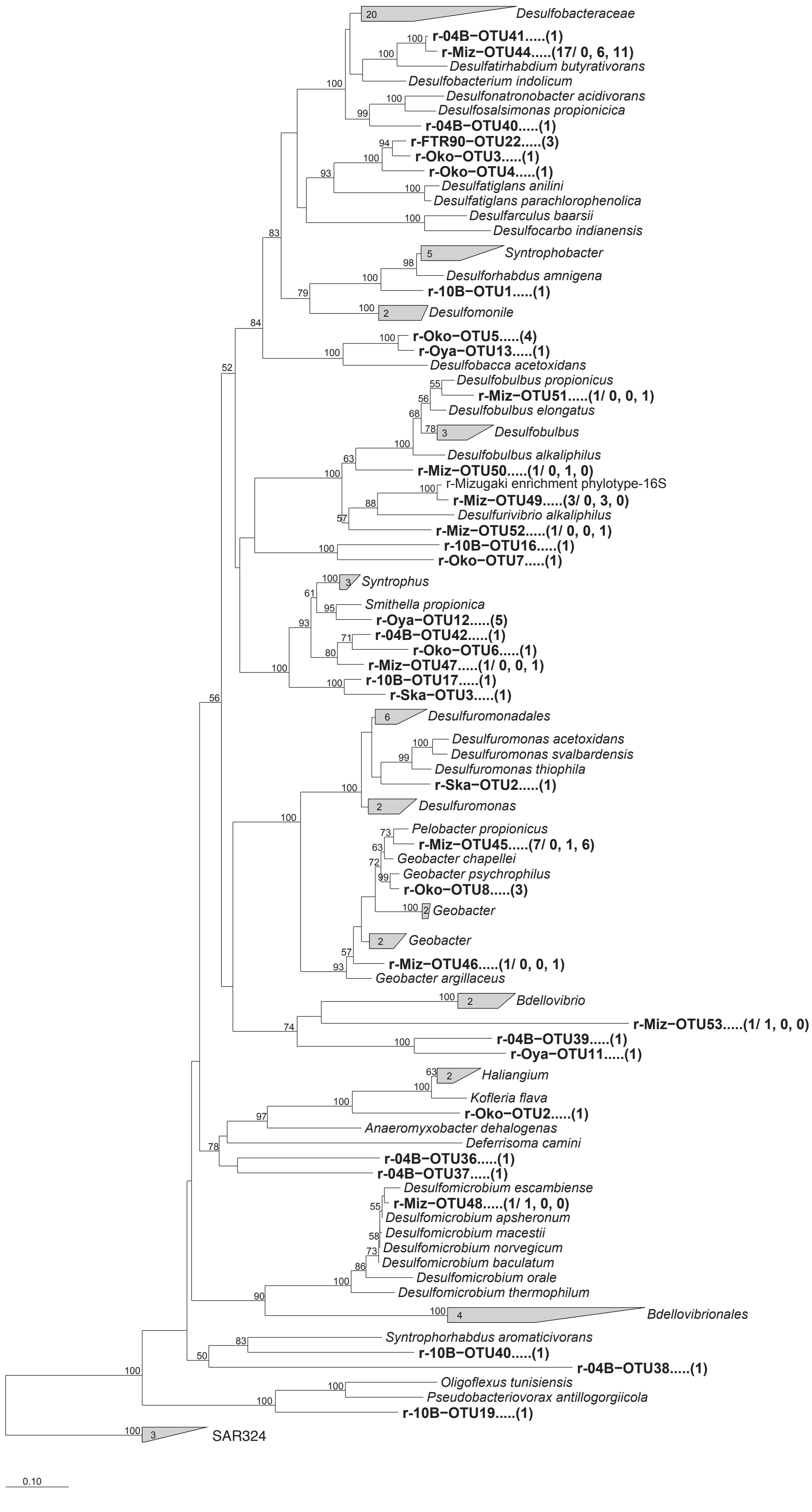

Desulfobacteraceae
r-04B−OTU41.....(1)
r-Miz−OTU44.....(17/ 0, 6, 11)
Desulfatirhabdium butyrativorans
Desulfobacterium indolicum
Desulfonatronobacter acidivorans
Desulfosalsimonas propionicica
r-04B−OTU40.....(1)
r-FTR90−OTU22.....(3)
r-Oko−OTU3.....(1)
r-Oko−OTU4.....(1)
Desulfatiglans anilini
Desulfatiglans parachlorophenolica
Desulfarculus baarsii
Desulfocarbo indianensis
Syntrophobacter
Desulforhabdus amnigena
r-10B−OTU1.....(1)
Desulfomonile
r-Oko−OTU5.....(4)
r-Oya−OTU13.....(1)
Desulfobacca acetoxidans
Desulfobulbus propionicus
r-Miz−OTU51.....(1/ 0, 0, 1)
Desulfobulbus elongatus
Desulfobulbus
Desulfobulbus alkaliphilus
r-Miz−OTU50.....(1/ 0, 1, 0)
r-Mizugaki enrichment phylotype-16S
r-Miz−OTU49.....(3/ 0, 3, 0)
Desulfurivibrio alkaliphilus
r-Miz−OTU52.....(1/ 0, 0, 1)
r-10B−OTU16.....(1)
r-Oko−OTU7.....(1)
Syntrophus
Smithella propionica
r-Oya−OTU12.....(5)
r-04B−OTU42.....(1)
r-Oko−OTU6.....(1)
r-Miz−OTU47.....(1/ 0, 0, 1)
r-10B−OTU17.....(1)
r-Ska−OTU3.....(1)
Desulfuromonadales
Desulfuromonas acetoxidans
Desulfuromonas svalbardensis
Desulfuromonas thiophila
r-Ska−OTU2.....(1)
Desulfuromonas
Pelobacter propionicus
r-Miz−OTU45.....(7/ 0, 1, 6)
Geobacter chapellei
Geobacter psychrophilus
r-Oko−OTU8.....(3)
Geobacter
Geobacter
r-Miz−OTU46.....(1/ 0, 0, 1)
Geobacter argillaceus
Bdellovibrio
r-Miz−OTU53.....(1/ 1, 0, 0)
r-04B−OTU39.....(1)
r-Oya−OTU11.....(1)
Haliangium
Kofleria flava
r-Oko−OTU2.....(1)
Anaeromyxobacter dehalogenas
Deferrisoma camini
r-04B−OTU36.....(1)
r-04B−OTU37.....(1)
Desulfomicrobium escambiense
r-Miz−OTU48.....(1/ 1, 0, 0)
Desulfomicrobium apsheronum
Desulfomicrobium macestii
Desulfomicrobium norvegicum
Desulfomicrobium baculatum
Desulfomicrobium orale
Desulfomicrobium thermophilum
Bdellovibrionales
Syntrophorhabdus aromaticivorans
r-10B−OTU40.....(1)
r-04B−OTU38.....(1)
Oligoflexus tunisiensis
Pseudobacteriovorax antillogorgiicola
r-10B−OTU19.....(1)
SAR324
0.10

Fig. S8a

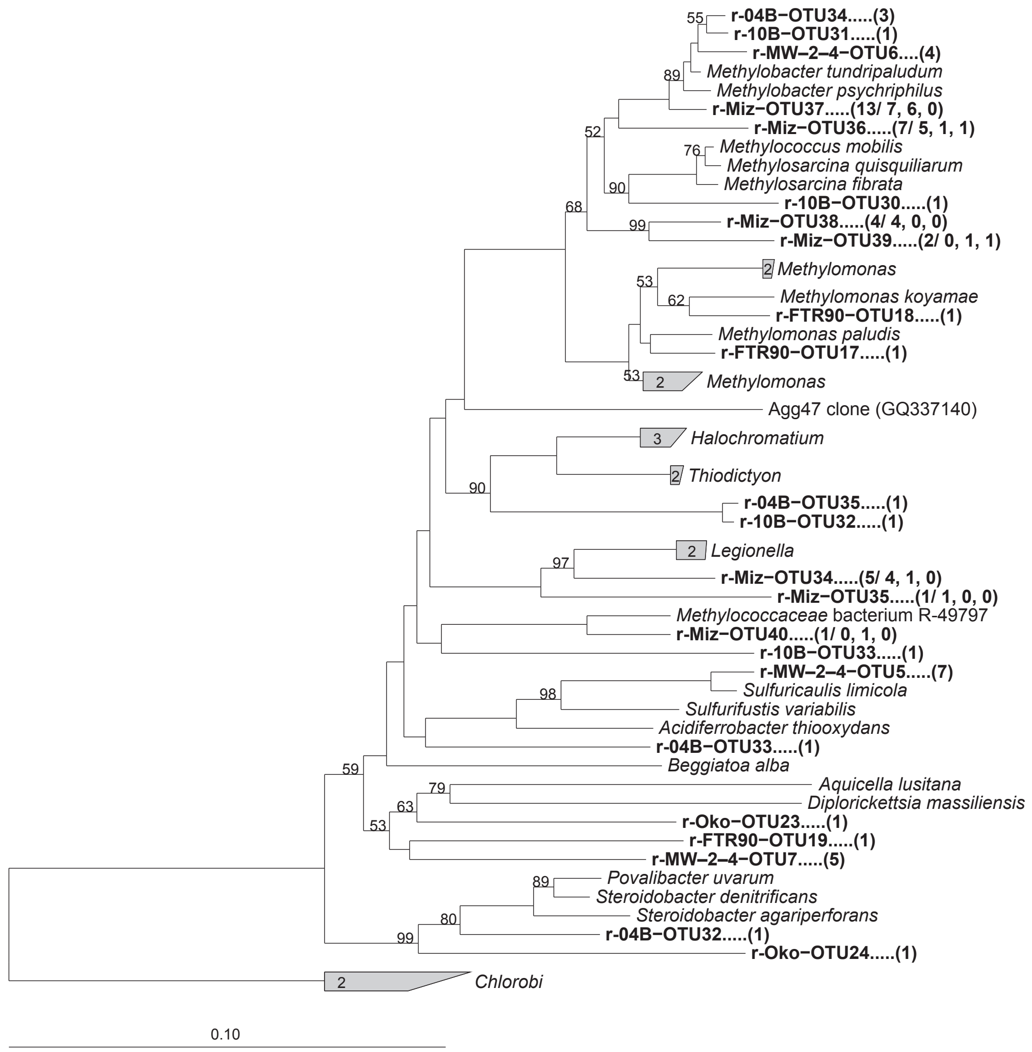

r-04B–OTU34.....(3)
r-10B–OTU31.....(1)
r-MW–2–4–OTU6....(4)
Methylobacter tundripaludum
Methylobacter psychriphilus
r-Miz–OTU37.....(13/ 7, 6, 0)
r-Miz–OTU36.....(7/ 5, 1, 1)
Methylococcus mobilis
Methylosarcina quisquiliarum
Methylosarcina fibrata
r-10B–OTU30.....(1)
r-Miz–OTU38.....(4/ 4, 0, 0)
r-Miz–OTU39.....(2/ 0, 1, 1)
Methylomonas
Methylomonas koyamae
r-FTR90–OTU18.....(1)
Methylomonas paludis
r-FTR90–OTU17.....(1)
Methylomonas
Agg47 clone (GQ337140)
Halochromatium
Thiodictyon
r-04B–OTU35.....(1)
r-10B–OTU32.....(1)
Legionella
r-Miz–OTU34.....(5/ 4, 1, 0)
r-Miz–OTU35.....(1/ 1, 0, 0)
Methylococcaceae bacterium R-49797
r-Miz–OTU40.....(1/ 0, 1, 0)
r-10B–OTU33.....(1)
r-MW–2–4–OTU5.....(7)
Sulfuricaulis limicola
Sulfurifustis variabilis
Acidiferrobacter thiooxydans
r-04B–OTU33.....(1)
Beggiatoa alba
Aquicella lusitana
Diplorickettsia massiliensis
r-Oko–OTU23.....(1)
r-FTR90–OTU19.....(1)
r-MW–2–4–OTU7.....(5)
Povalibacter uvarum
Steroidobacter denitrificans
Steroidobacter agariperforans
r-04B–OTU32.....(1)
r-Oko–OTU24.....(1)
Chlorobi
0.10

Fig. S8b

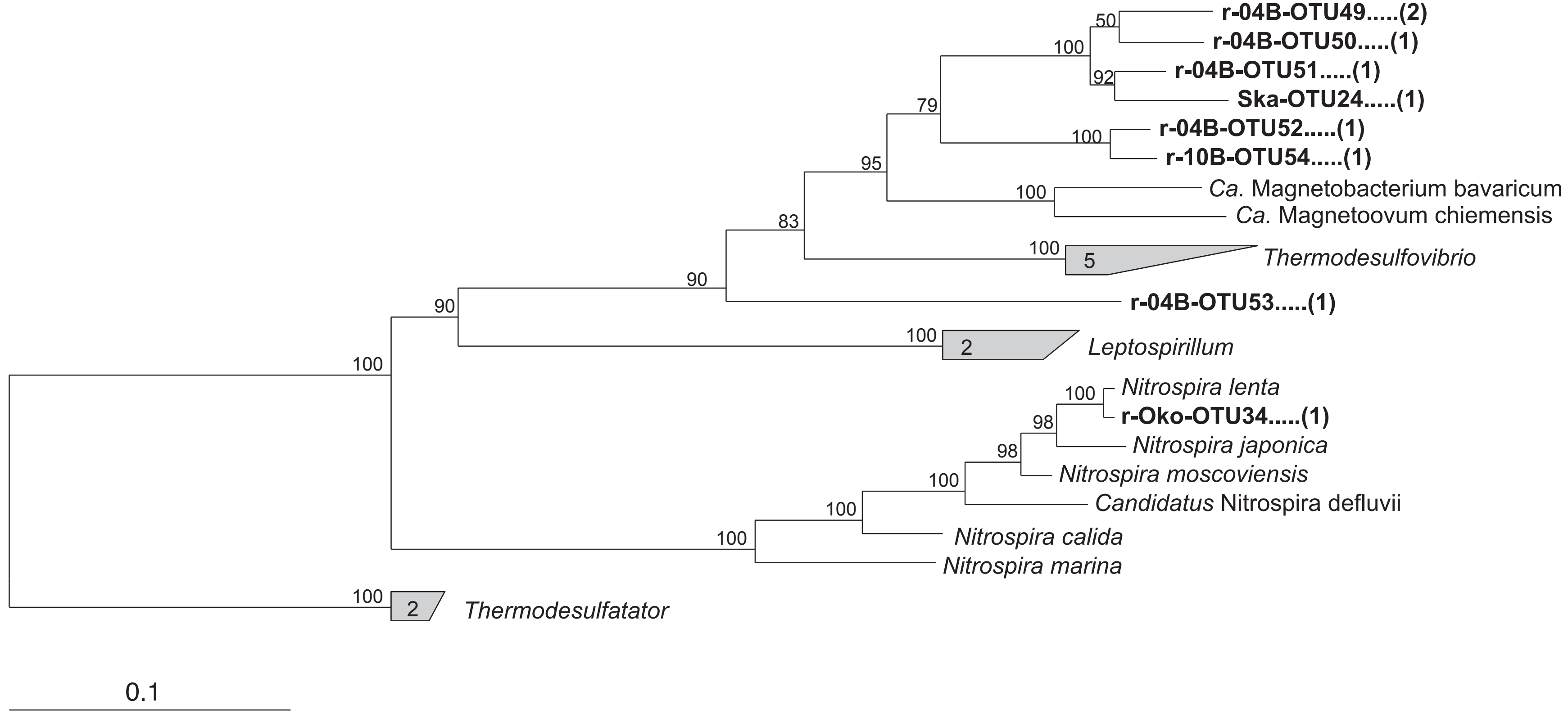
r-04B-OTU49.....(2)
r-04B-OTU50.....(1)
r-04B-OTU51.....(1)
Ska-OTU24.....(1)
r-04B-OTU52.....(1)
r-10B-OTU54.....(1)
Ca. Magnetobacterium bavaricum
Ca. Magnetoovum chiemensis
Thermodesulfovibrio
r-04B-OTU53.....(1)
Leptospirillum
Nitrospira lenta
r-Oko-OTU34.....(1)
Nitrospira japonica
Nitrospira moscoviensis
Candidatus Nitrospira defluvii
Nitrospira calida
Nitrospira marina
Thermodesulfatator
50
100
92
79
100
95
100
83
100
90
90
100
100
100
98
98
100
100
100
100
5
2
2
0.1

Fig. S8c

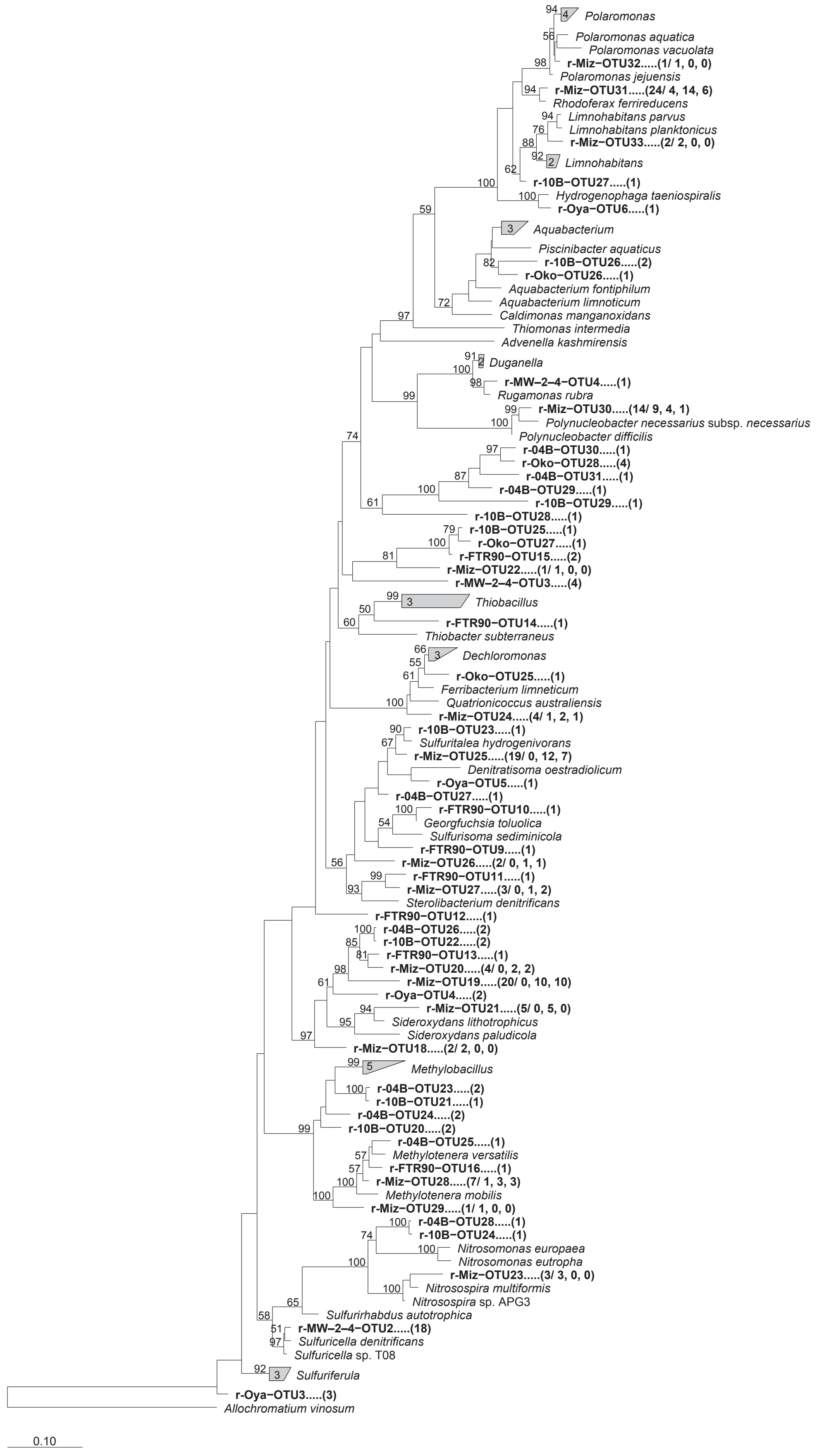

Polaromonas
Polaromonas aquatica
Polaromonas vacuolata
r-Miz−OTU32.....(1/ 1, 0, 0)
Polaromonas jejuensis
r-Miz−OTU31.....(24/ 4, 14, 6)
Rhodoferax ferrireducens
Limnohabitans parvus
Limnohabitans planktonicus
r-Miz−OTU33.....(2/ 2, 0, 0)
Limnohabitans
r-10B−OTU27.....(1)
Hydrogenophaga taeniospiralis
r-Oya−OTU6.....(1)
Aquabacterium
Piscinibacter aquaticus
r-10B−OTU26.....(2)
r-Oko−OTU26.....(1)
Aquabacterium fontiphilum
Aquabacterium limnoticum
Caldimonas manganoxidans
Thiomonas intermedia
Advenella kashmirensis
Duganella
r-MW−2−4−OTU4.....(1)
Rugamonas rubra
r-Miz−OTU30.....(14/ 9, 4, 1)
Polynucleobacter necessarius subsp. necessarius
Polynucleobacter difficilis
r-04B−OTU30.....(1)
r-Oko−OTU28.....(4)
r-04B−OTU31.....(1)
r-04B−OTU29.....(1)
r-10B−OTU29.....(1)
r-10B−OTU28.....(1)
r-10B−OTU25.....(1)
r-Oko−OTU27.....(1)
r-FTR90−OTU15.....(2)
r-Miz−OTU22.....(1/ 1, 0, 0)
r-MW−2−4−OTU3.....(4)
Thiobacillus
r-FTR90−OTU14.....(1)
Thiobacter subterraneus
Dechloromonas
r-Oko−OTU25.....(1)
Ferribacterium limneticum
Quatrionicoccus australiensis
r-Miz−OTU24.....(4/ 1, 2, 1)
r-10B−OTU23.....(1)
Sulfuritalea hydrogenivorans
r-Miz−OTU25.....(19/ 0, 12, 7)
Denitratisoma oestradiolicum
r-Oya−OTU5.....(1)
r-04B−OTU27.....(1)
r-FTR90−OTU10.....(1)
Georgfuchsia toluolica
Sulfurisoma sediminicola
r-FTR90−OTU9.....(1)
r-Miz−OTU26.....(2/ 0, 1, 1)
r-FTR90−OTU11.....(1)
r-Miz−OTU27.....(3/ 0, 1, 2)
Sterolibacterium denitrificans
r-FTR90−OTU12.....(1)
r-04B−OTU26.....(2)
r-10B−OTU22.....(2)
r-FTR90−OTU13.....(1)
r-Miz−OTU20.....(4/ 0, 2, 2)
r-Miz−OTU19.....(20/ 0, 10, 10)
r-Oya−OTU4.....(2)
r-Miz−OTU21.....(5/ 0, 5, 0)
Sideroxydans lithotrophicus
Sideroxydans paludicola
r-Miz−OTU18.....(2/ 2, 0, 0)
Methylobacillus
r-04B−OTU23.....(2)
r-10B−OTU21.....(1)
r-04B−OTU24.....(2)
r-10B−OTU20.....(2)
r-04B−OTU25.....(1)
Methylotenera versatilis
r-FTR90−OTU16.....(1)
r-Miz−OTU28.....(7/ 1, 3, 3)
Methylotenera mobilis
r-Miz−OTU29.....(1/ 1, 0, 0)
r-04B−OTU28.....(1)
r-10B−OTU24.....(1)
Nitrosomonas europaea
Nitrosomonas eutropha
r-Miz−OTU23.....(3/ 3, 0, 0)
Nitrosospira multiformis
Nitrosospira sp. APG3
Sulfurirhabdus autotrophica
r-MW−2−4−OTU2.....(18)
Sulfuricella denitrificans
Sulfuricella sp. T08
Sulfuriferula
r-Oya−OTU3.....(3)
Allochromatium vinosum
0.10

Fig. S8d

Fig. S8. Phylogenetic positions of 16S rRNA OTUs obtained in this study within the *Deltaproteobacteria* (a), *Gammaproteobacteria* (b), *Nitrospiraceae* (c) and *Betaproteobacteria* (d). All trees were constructed by ML method with 100 bootstrap analysis (bootstrap values ≥ 50% are shown at the nodes). OTUs are highlighted in bold type. The numbers in parentheses are the number of clones belonging to each OTU. The clone numbers of Miz are shown with respect to each water depth (total clone number/ 25m/ 35m/ 43m).

References

1. Widdel, F. & Bak, F. Gram-negative mesophilicsulfate-reducing bacteria. In *The Prokaryotes*. (eds Balows, A.,Trüper, H.G., Dworkin, M. & Harder, W.) 3352–3378 (Springer, 1992).
2. Lovley, D. R. Dissimilatory Fe(III)- and Mn(IV)-reducing prokaryotes. In *The Prokaryotes*. (eds Rosenberg, E., DeLong, F., Lory, S., Stackebrandt, E. & Thompson, F. 287–308 (Springer, 2013).
3. Kojima, H. *et al.* Distribution of putative denitrifying methane oxidizing bacteria in sediment of a freshwater lake, Lake Biwa. *Syst. Appl. Microbiol.* 35, 233–238 (2012).
4. Kojima, H., Watanabe, T., Iwata, T. & Fukui, M. Identification of major planktonic sulfur oxidizers in stratified freshwater lake. *PLoS ONE* 9, e93877 (2014).
5. Kojima, H. *et al.* Community structure of planktonic methane-oxidizing bacteria in a subtropical reservoir characterized by dominance of phylotype closely related to nitrite reducer. *Sci Rep* 4, 5728 (2014).
